# Regioselective Iodination of Arenes Using Iron- or Silver-Catalyzed Activation of *N*‑Iodosaccharin

**DOI:** 10.1021/acs.joc.5c03183

**Published:** 2026-01-27

**Authors:** Pankaj K. Majhi, Catherine M. Fleming, Andrew Sutherland

**Affiliations:** School of Chemistry, 3526 University of Glasgow, The Joseph Black Building, Glasgow G12 8QQ, U.K.

## Abstract

Aryl iodides are privileged intermediates in organic synthesis, underpinning cross-coupling chemistry, late-stage functionalization, and radiolabeling in medicinal chemistry. However, regioselective arene iodination remains a challenge, as traditional electrophilic iodination methods often require harsh conditions, exhibit poor selectivity, or suffer from limited functional group tolerance and substrate scope. We report a rapid and regioselective arene iodination enabled by Lewis acid activation of *N*-iodosaccharin. Iron­(III) chloride and silver­(I) triflimide were found to catalyze the efficient iodination of a broad range of electron-rich arenes at room temperature. The method displayed broad functional group tolerance and was applicable to complex substrates, including natural products and pharmaceuticals. Furthermore, the iodination was found to be compatible with cross-coupling reactions, allowing one-pot halogenation and arylation sequences for direct access to biaryl compounds.

## Graphical abstract

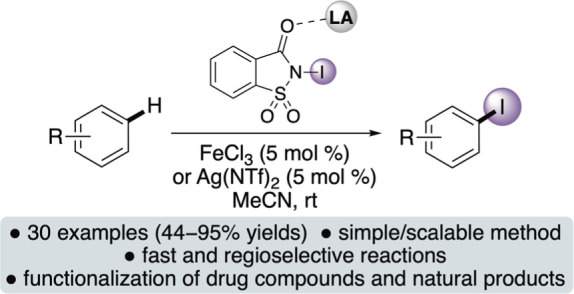

## Introduction

Aryl iodides occupy a prominent position in modern organic and medicinal chemistry due to the low C–I bond dissociation energy and the subsequent high reactivity.^1^ These properties enable superior leaving-group behavior under milder conditions than aryl bromides or chlorides, making aryl iodides particularly effective in Pd- or Ni-catalyzed cross-couplings such as Suzuki–Miyaura, Sonogashira, Heck, and Buchwald–Hartwig reactions for the construction of diverse carbon–carbon and carbon–heteroatom scaffolds.^2–4^ In medicinal chemistry, they perform dual roles: first, as key precursors to complex bioactive molecules;^5^ second, as target compounds in radiochemistry applications.^6^ For example, biologically active arenes labeled with iodine-123 and iodine-125 are widely used for the single photon emission computed tomography imaging of diseases, such as cancer and neurodegenerative disorders.^7^

The importance of iodoarenes has resulted in the development of many synthetic strategies for the preparation of this compound class. Although a wide range of modern methods have been developed, such as iodination of arene functional handles such as bromides,^8^ boronates,^9^ or diazonium salts^10^ and processes including directed metal-catalyzed C–H iodination,^11^ the use of electrophilic aromatic iodination remains a key approach. Traditional methods for electrophilic aromatic substitution that use iodine in the presence of oxidants such as nitric acid or iodic acid suffer from harsh conditions, moderate yields, and poor regioselectivity. To overcome these limitations, methods involving the activation of reagents such as *N*-iodosuccinimide (NIS) have been developed. Olah and co-workers used BF_3_ in water to activate NIS for the iodination of deactivated arenes,^12^ while indium,^13^ gold,^14^ iron,^15^ and silver-based Lewis acid activation of NIS was used for the regioselective iodination of electron-rich arenes (Figure 1a).^16^ More recently, Du and co-workers reported a dual catalytic method involving triflic acid as a Lewis acid and thianthrene as a Lewis base for the NIS-activated halogenation of arenes (Figure 1b).^17^ Other mild methods include the use of thiourea organocatalysts to activate 1,3-diiodo-5,5-dimethylhydantoin^18^ and the Ritter group’s application of a sulfonyl-based hypoiodite, formed from iodine and silver salts such as silver mesylate (Figure 1c).^19^ These methods typically allow mild and efficient iodination with high regioselectivity, which is directed by the electronics of the substituents.

**Figure 1:**
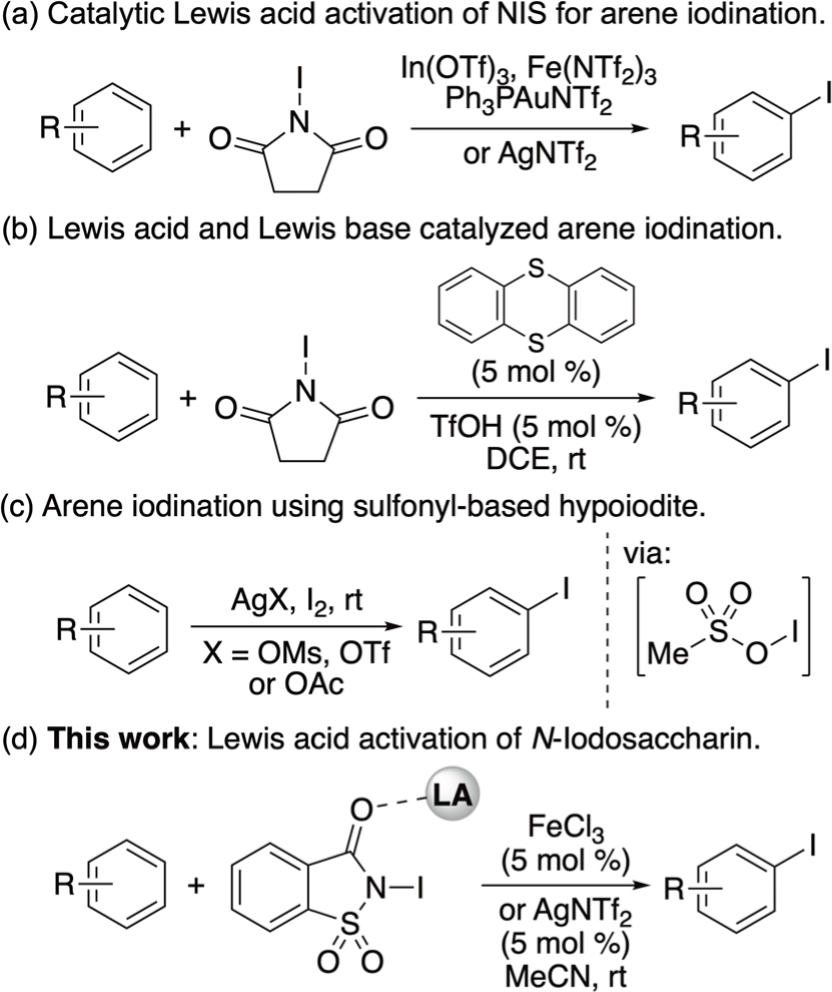
Methods for the regioselective iodination of activated arenes.

Despite recent advances, electrophilic aromatic iodination methods still have limitations. Many of the approaches generate regioisomeric impurities that are difficult to separate.^13,14,16,19^ Others require prolonged reaction times, particularly when applied to less activated arenes.^13,14^ For example, our previously reported iron­(III) triflimide-catalyzed activation of NIS gave a 93:7 mixture of *p*/*o*-iodinated anisoles.^15^ In the case of substrates bearing electron-withdrawing groups, elevated temperatures (50 °C) or extended reaction times of up to 24 h were necessary.^15^ To address these limitations, we sought to employ a more reactive iodination reagent. In 2000, Dolenc reported the use of *N*-iodosaccharin as a reagent for the noncatalyzed iodination of six activated arenes.^20^ Although promising, this method required reaction times of up to 12 h and gave regioisomeric mixtures for some substrates. Building on prior success using Lewis acids to enhance the reactivity of NIS for iodination, we proposed that the combination of a Lewis acid and *N*-iodosaccharin would generate a bulkier, yet more reactive intermediate, thereby enabling fast and regioselective iodination of arenes (Figure 1d). Here, we report the Lewis acid-catalyzed activation of *N*-iodosaccharin for the rapid, efficient, and highly regioselective room temperature iodination of arenes. We also demonstrate the synthetic utility of this method through the late-stage functionalization of natural products and drug derivatives, as well as for the one-pot, two-step synthesis of biaryl compounds.

## Results and Discussion

Based on the known reactivity of metal triflimides as super Lewis acids,^21^ initial studies examined iron­(III) triflimide as a catalyst for the *N*-iodosaccharin-mediated iodination of anisole (**1a**) (Table 1). Prepared in situ from iron­(III) chloride and the readily available ionic liquid [BMIM]­NTf_2_,^15^ a 10 mol % catalyst loading gave *p*-iodoanisole (**3a**) in 95% yield following a reaction time of 0.5 h (entry 1). Notably, analysis of the crude reaction mixture using ^1^H NMR spectroscopy revealed excellent regioselectivity with a >99:1 ratio of the *para*-isomer. Similar results were obtained using a lower catalyst loading (5 mol %, entry 2) and the softer Lewis acid, silver triflimide (entry 3).^16^ Due to the rapid and efficient nature of these reactions, more readily available metal chloride Lewis acids were next evaluated (entries 4–6). These results demonstrated that a noncoordinating triflimide ligand was not necessary and that the use of AlCl_3_, AuCl_3_, or FeCl_3_ could effectively activate *N*-iodosaccharin (**2**) for similarly fast, efficient, and regioselective reactions. Using FeCl_3_ as the preferred catalyst, further optimization studies were undertaken. Although a reaction with lower catalyst loading (2.5 mol %) was complete after 0.5 h, the isolated yield dropped to 84% (entry 7). Thus, an additional screen to identify a nonchlorinated solvent was performed using 5 mol % loading (entries 8–10). This revealed acetonitrile as the optimal solvent, which gave *p*-iodoanisole in 95% yield and >99:1 regioselectivity after 0.5 h (entry 10).

**Table 1:**
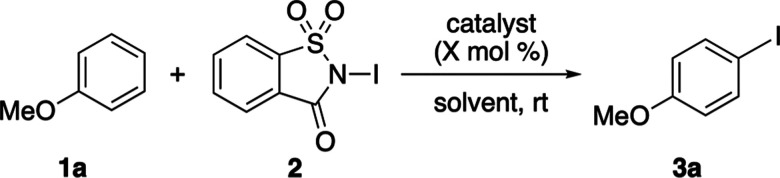
Optimization Studies for the Lewis Acid-Catalyzed Iodination of Anisole (**1a**)­^a^

entry	catalyst	cat. loading (mol %)	solvent	time (h)	yield (%)^b^
1	Fe(NTf_2_)_3_	10	CHCl_3_	0.5	95
2	Fe(NTf_2_)_3_	5	CHCl_3_	0.5	94
3	AgNTf_2_	5	CHCl_3_	0.5	89
4	AlCl_3_	5	CHCl_3_	0.5	93
5	AuCl_3_	5	CHCl_3_	0.5	95
6	FeCl_3_	5	CHCl_3_	0.5	90
7	FeCl_3_	2.5	CHCl_3_	0.5	84
8	FeCl_3_	5	THF	1	87
9	FeCl_3_	5	EtOAc	1.5	72
10	FeCl_3_	5	MeCN	0.5	95

a Reactions were done using *N*-iodosaccharin (1.3 equiv) and solvent (0.5 M).

b Isolated yield.

Following the optimization of the iodination reaction, the substrate scope was explored (Scheme 1). A variety of activated arenes underwent efficient iodination, typically reaching completion within 0.5 h to afford single regioisomers in high yields. The method proved scalable, delivering gram quantities of **3a** in quantitative yields after 1 h. Among the substrate classes examined, only simple aryl alcohols, such as phenol (**1b**) and 2-naphthol (**1w**) (vide infra), proved to be problematic. In these cases, colored reaction solutions and complex product mixtures were observed, likely arising via the formation of iron aryloxide species.^22^ For phenol, rapid and efficient iodination with *N*-iodosaccharin (**2**) proceeded in the absence of a catalyst, affording **3b** in an 87% isolated yield after 1 h. In contrast, no analogous iron­(III) byproduct formation was observed with aniline substrates under the optimized conditions.^23^ All anilines delivered the corresponding iodinated products in 62–90% yields. To distinguish the role of iron­(III) activation from potential Brønsted acid catalysis arising from HCl generated upon reaction of aryl amines with FeCl_3_, the iodination of aniline (**1c**) using *N*-iodosaccharin was conducted in the presence of HCl (5 mol %). Under these conditions, the reaction required 5.6 h to reach completion, compared to 0.5 h when catalyzed by FeCl_3_ (5 mol %). This >10-fold rate enhancement using FeCl_3_ emphasizes the critical role of iron­(III) activation, rather than Brønsted acid catalysis in promoting aryl amine iodination.

**Scheme 1:**
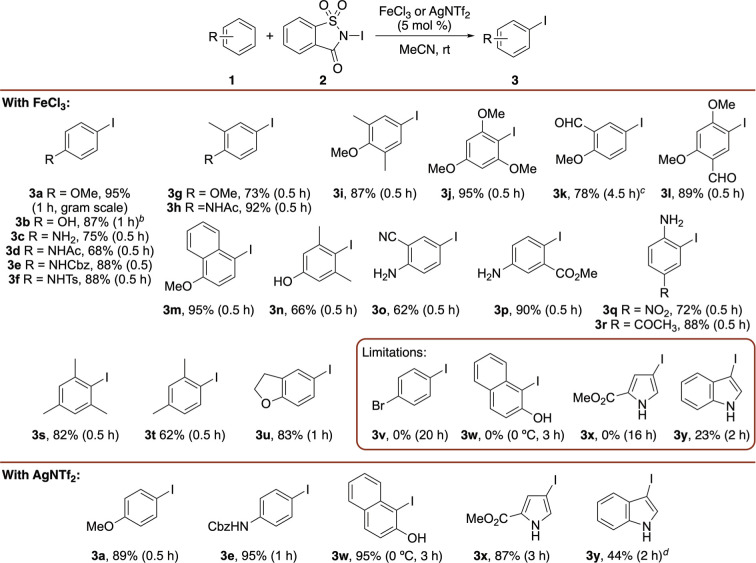
Scope of Iron- and Silver-Catalyzed Arene Iodination^a^

a Reactions were done using *N*-iodosaccharin (1.3 equiv) and MeCN (0.5 M).
b No catalyst.
c Using Fe­(NTf_2_)_3_ (15 mol %).
d Using AgNTf_2_ (10 mol %).

The important role of Lewis acid activation of *N*-iodosaccharin was further evidenced with less activated examples such as 2-methoxybenzaldehyde (**1k**), which required the use of Fe­(NTf_2_)_3_, resulting in a 78% yield of **3k** after 4.5 h. In contrast, the uncatalyzed reaction showed only 7% conversion after 1.5 h and 32% after 20 h. The general FeCl_3_ reaction was effective with other substrates bearing strongly deactivating groups (**3o–3r**) and more hindered di-*ortho* substitution (**3j**). Less activated substrates such as mesitylene (**1s**) and *m*-xylene (**1t**) also produced the corresponding iodinated products in good yields (62–82%) within 0.5 h.

Despite the overall success of these reactions, certain limitations were encountered. For example, attempts to iodinate deactivated arenes, such as bromobenzene (**1v**), resulted in no conversion even after prolonged reaction times (20 h). As discussed, iodination of compounds such as 2-naphthol (**1w**) and heterocycles such as pyrrole (**1x**) and indole (**1y**) produced darkened reaction mixtures and either no product or low yields. These observations suggested that the hard iron­(III) Lewis acid was not compatible with the hydroxyl and amine coordinating groups associated with this type of substrate. To address this issue, iodination of these compounds was explored by using the softer silver­(I) Lewis acid catalyst, AgNTf_2_. When applied to standard activated arenes such as anisole (**1a**) and *N*-Cbz protected aniline **1e**, these produced yields comparable to those obtained with FeCl_3_. Notable improvements were observed for previously problematic substrates. Silver­(I)-catalyzed iodination of 2-naphthol and pyrrole proceeded as clear, homogeneous reaction mixtures, affording **3w** and **3x** in excellent yields after 3 h. The reaction of indole **1y** was also improved, delivering **3y** in a 44% yield.

To assess the applicability of this reaction for selective and efficient iodination of more complex compounds, it was investigated using various small molecule drugs and natural products (Scheme 2). The esters of gemfibrozil, a preventative heart disease drug, and the painkillers diclofenac and naproxen were all rapidly iodinated using FeCl_3_ catalysis (5 mol %), giving the products (**5a–5c**) as single regioisomers in excellent yields (90–94%). In the case of diclofenac, the reaction was completely selective for the more electron-rich aromatic ring. Similar results were obtained for iron­(III)-catalyzed iodination of the muscle relaxant, metaxalone, which gave **5d** in 87% yield after 15 min. Iodination of gemfibrozil derivative **4a** was also performed using silver­(I) triflimide, which as expected, gave **5a** in high yield but with a slightly longer reaction time (1 h). As with simpler arenes, attempted iron-catalyzed iodination of highly electron-rich compounds such as the natural product and psoriasis drug methoxsalen (**4e**) led to complex mixtures with the desired products isolated in low yields. Instead, the reaction with *N*-iodosaccharin in the absence of a Lewis acid catalyst was investigated and gave the iodinated product **5e** after 1 h in 65% yield. The importance of catalyst use was demonstrated with the regioselective iodination of β-estradiol dimethyl ether (**4f**). Electrophilic aromatic substitution reactions of β-estradiol and derivatives such as **4f** often result in mixtures of 2- and 4-regioisomers.^24^ For example, halogenation using various reagents has previously given the two regioisomers in ratios ranging from 1:1 to 4:1 in favor of the 2-isomer.^25^ An improved protocol was reported using In­(III)-catalyzed iodination with NIS, which gave the 2-isomer in 80% yield but also formed the 2,4-di-iodinated product in 7% yield.^13^ In comparison, iron­(III)-catalyzed iodination of β-estradiol dimethyl ether (**4f**) using *N*-iodosaccharin gave 2-isomer **5f** as the sole product in quantitative yield after 0.5 h. This result highlights the effectiveness of this method and the beneficial combination of the iron­(III) Lewis acid and *N*-iodosaccharin for achieving the selective and efficient iodination of complex substrates under mild conditions.

**Scheme 2:**
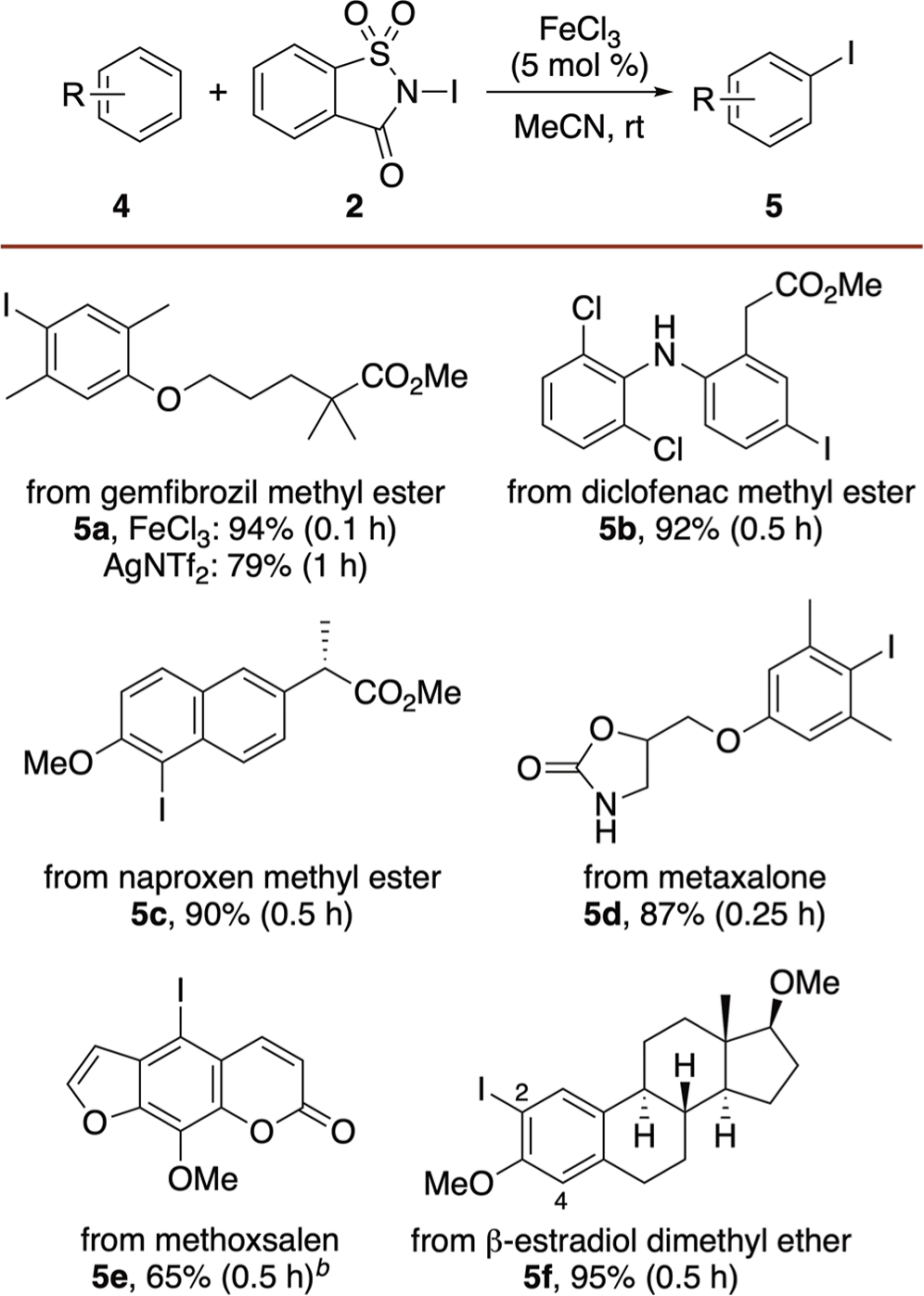
Late-Stage Functionalization of Bioactive Compounds^a^

a Reactions were done using *N*-iodosaccharin (1.3 equiv) and MeCN (0.5 M).
b No catalyst.

Based on the success of Lewis acid-catalyzed iodination using *N*-iodosaccharin, subsequent applications of this process were investigated. Traditionally, biaryl compounds have been prepared via cross-coupling reactions using prefunctionalized arenes or alternative strategies such as metal-catalyzed C–H bond arylation.^26^ Given that the iron­(III)-catalyzed iodination method using *N*-iodosaccharin proceeded under mild conditions and afforded clean products, it was proposed that a one-pot sequence combining this method with a cross-coupling reaction would access the biaryl compounds directly. Such an approach would enable aryl C–C bond formation directly from aryl C–H bonds. As a proof-of-concept, one-pot iodination-arylation of anisole (**1a**) was investigated (Scheme 3). Following iodination using *N*-iodosaccharin (**2**) and FeCl_3_ (5 mol %), subsequent addition of the Buchwald precatalyst,^27^ XPhos Pd G2, and phenyl boronic acid produced aryl analogue **6a**. A 5 mol % loading of the palladium precatalyst provided the highest conversion, resulting in a 76% overall yield. The use of naphthyl- and 4-cyanophenyl boronic acid also allowed the one-pot synthesis of **6b** and **6c** in 68% and 56% yield, respectively. To demonstrate the application of this one-pot process with a more complex substrate, it was used for the regioselective arylation of naproxen methyl ester **4c**. Iron-catalyzed iodination, followed by a Suzuki–Miyaura cross-coupling reaction with phenylboronic acid, gave arylated product **6d** in 80% yield. Chiral HPLC of **6d** showed an enantiomeric ratio of 99.6:0.4 (see Supporting Information), confirming that both the iron-catalyzed iodination and Suzuki cross-coupling reaction are compatible with compounds containing sensitive stereogenic centers. Although demonstrated with just a few examples, these results highlight the potential of iron-catalyzed iodination with *N*-iodosaccharin for the one-pot, late-stage functionalization of arenes.

**Scheme 3:**
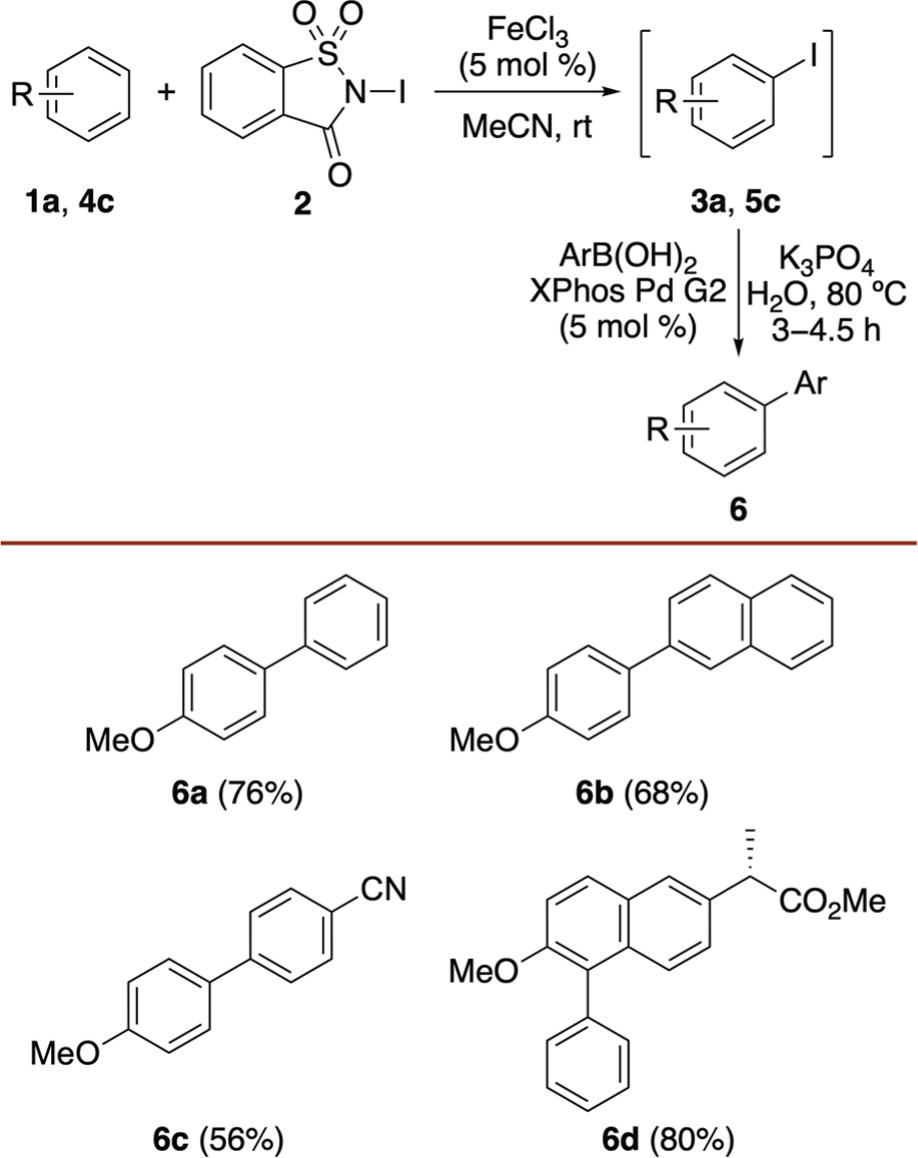
One-Pot Iodination and Arylation Reaction^a^ ^,^ ^b^

a Iodination reactions were done using *N*-iodosaccharin (1.3 equiv) and MeCN (0.5 M).
b Suzuki–Miyaura reactions were done using aryl boronic acid (1.5 equiv) and 0.17 M reaction solution.

## Conclusions

In summary, a regioselective iodination of electron-rich arenes via Lewis acid-catalyzed activation of the underutilized *N*-iodosaccharin reagent has been developed. Using iron­(III) chloride or silver­(I) triflimide, the reaction proceeds rapidly at room temperature, displays broad functional group tolerance, and is applicable to structurally complex substrates, including natural products and pharmaceuticals. Its compatibility with cross-coupling reactions was demonstrated with one-pot halogenation-arylation sequences, highlighting its potential utility for synthetic and medicinal chemistry applications. Current work is underway to fully explore the compatibility of this halogenation reaction with other arene cross-coupling processes.

## Experimental Section

All reagents and starting materials were obtained from commercial sources and used as received. Reactions were performed in air unless otherwise mentioned. All reactions performed at elevated temperatures were heated by using an oil bath. Brine refers to a saturated aqueous solution of sodium chloride. Flash column chromatography was performed using silica gel 60 (40–63 μm). Aluminum-backed plates precoated with silica gel 60F_254_ were used for thin layer chromatography and were visualized with a UV lamp or by staining with potassium permanganate, vanillin, or ninhydrin. ^1^H NMR spectra were recorded on a NMR spectrometer at either 400 or 500 MHz, and data are reported as follows: chemical shift in ppm relative to the solvent as internal standard (CHCl_3_, δ 7.26 ppm; CH_3_OH, δ 3.31 ppm; DMSO_,_ δ 2.50), multiplicity (s = singlet, d = doublet, t = triplet, q = quartet, m = multiplet or overlap of nonequivalent resonances, integration). ^13^C NMR spectra were recorded on an NMR spectrometer at either 101 or 126 MHz, and data are reported as follows: chemical shift in ppm relative to tetramethylsilane or the solvent as an internal standard (CDCl_3_, δ 77.2 ppm; CD_3_OD, δ 49.0 ppm; DMSO-*d*_6,_ δ 39.5). Infrared spectra were recorded on an FTIR spectrometer; wavenumbers are indicated in cm^–1^. Mass spectra were recorded using electrospray techniques. HRMS spectra were recorded using quadrupole time-of-flight (Q-TOF) mass spectrometers. Melting points are uncorrected. Optical rotations were determined as solutions irradiating with the sodium D line (λ = 589 nm) using a polarimeter. [α]_D_ values are given in units of 10^–1^ deg cm^–1^ g^–1^. Chiral HPLC methods were calibrated with the corresponding racemic mixtures.

### Synthesis of *N*-Iodosaccharin (**2**)^20^

*N*-Iodosaccharin was synthesized by following the reported procedure over two steps: Step 1: silver nitrate (8.5 g, 50 mmol) was dissolved in water (50 mL), heated to 80 °C, and a solution of sodium saccharin monohydrate (11.4 g, 51 mmol) in water (50 mL) was added dropwise with stirring. The white precipitate was filtered, washed with water and acetone, and dried in air, and the silver salt of saccharin (13.0 g, 89%) was obtained as a white solid. Step 2: the silver salt of *N*-iodosaccharin (10.0 g, 34.4 mmol) and iodine (8.9 g, 35.1 mmol) were stirred in acetone (100 mL) at room temperature in the dark. After 6 h, precipitated silver iodide was filtered off, and the filtrate was evaporated under reduced pressure. The resulting solid was washed with hexane and purified by recrystallization using a THF/hexane ratio (1:1). Pale-yellow crystals of *N*-iodosaccharin (**2**) (10.2 g, 95%) were obtained. mp 207–208 °C (lit.^20^ 206–208 °C); ^1^H NMR [400 MHz, (CD_3_)_2_CO]: δ 8.11 (ddd, *J* = 7.6, 1.3, 0.7 Hz, 1H), 8.05 (ddd, *J* = 7.3, 1.5, 0.7 Hz, 1H), 8.01–7.90 (m, 2H); ^13^C­{^1^H} NMR [101 MHz, (CD_3_)_2_CO)]: δ 162.4, 140.1, 135.6, 135.2, 128.3, 126.0, 122.2; MS (APCI) *m*/*z* 308 (M^+^, 100).

### Iron­(III)-Catalyzed General Procedure of Iodination

To a small, dry vial fitted with a magnetic stirrer were added *N*-iodosaccharin (**2**) (201 mg, 1.30 equiv), iron trichloride (5 mol %), and acetonitrile (1 mL) under an atmosphere of air. Substrate (1.00 equiv) was added. The mixture was stirred at room temperature. The reactions were stopped once all substrate was consumed. Reaction was monitored by TLC or NMR spectroscopy. When the reaction was completed, the reaction mixture was filtered through Celite with dichloromethane or ethyl acetate. The resulting filtrate was evaporated in vacuo and purified by silica column chromatography.

### 1-Iodo-4-methoxybenzene (**3a**)^28^

The reaction was performed as described in the general procedure using anisole (54.3 μL, 0.500 mmol, 1 equiv), *N*-iodosaccharin (**2**) (201 mg, 0.650 mmol, 1.3 equiv), iron trichloride (4.10 mg, 0.0250 mmol, 0.05 equiv), and acetonitrile (1.0 mL) for 0.5 h. 4-Iodoanisole (**3a**) (111 mg, 95%) was obtained as a white solid after purification by column chromatography with 10% ethyl acetate in hexane. mp 40–43 °C (lit.^28^ 43–45 °C); ^1^H NMR (400 MHz, CDCl_3_): δ 7.58–7.54 (m, 2H), 6.70–6.65 (m, 2H), 3.78 (s, 3H); ^13^C­{^1^H} NMR (101 MHz, CDCl_3_): δ 159.5, 138.2, 116.4, 82.7, 55.3; MS (EI) *m*/*z* 234 (M^+^, 96), 219 (55), 191 (12), 84 (100), 49 (66).

### 1-Iodo-4-methoxybenzene (**3a**): Gram Scale Reaction

The reaction was performed as described in the general procedure using anisole (0.54 mL, 5.0 mmol, 1 equiv), *N*-iodosaccharin (2.0 g, 6.5 mmol, 1.3 equiv), iron trichloride (41 mg, 0.25 mmol, 0.05 equiv), and acetonitrile (5 mL) for 1 h. 4-Iodoanisole (1.2 g, 95%) was obtained as a white solid after the purification by column chromatography with 10% ethyl acetate in hexane. Spectroscopic data as reported above.

### 4-Iodophenol (**3b**)^29^

The reaction was performed as described in the general procedure using phenol (47.0 mg, 0.500 mmol, 1 equiv), *N*-iodosaccharin (**2**) (201 mg, 0.650 mmol, 1.3 equiv), and acetonitrile (1.0 mL) for 1 h. 4-Iodophenol (**3b**) (96.0 mg, 87%) was obtained as a white solid after purification by column chromatography with 10% ethyl acetate in hexane. The data were consistent with the literature.^29^ mp 89–91 °C; ^1^H NMR (400 MHz, CDCl_3_): δ 7.52 (d, *J* = 8.8 Hz, 2H), 6.63 (d, *J* = 8.8 Hz, 2H), 4.83–4.69 (m, 1H); ^13^C­{^1^H} NMR (101 MHz, CDCl_3_): δ 155.3, 138.5, 117.8, 82.8; MS (EI) *m*/*z* 220 (M^+^, 100), 191 (3), 127 (6), 110 (5), 93 (37), 65 (20).

### 4-Iodoaniline (**3c**)^30^

The reaction was performed as described in the general procedure using aniline (46.0 μL, 0.500 mmol, 1 equiv), *N*-iodosaccharin (**2**) (201 mg, 0.650 mmol, 1.3 equiv), iron trichloride (4.10 mg, 0.0250 mmol, 0.05 equiv), and acetonitrile (1.0 mL) for 0.5 h. 4-Iodoaniline (**3c**) (82.0 mg, 75%) was obtained as an off-white solid after purification by column chromatography with 30% ethyl acetate in hexane. mp 53–55 °C (lit.^30^ 55–56.5 °C); ^1^H NMR (400 MHz, CDCl_3_): δ 7.43–7.38 (m, 2H), 6.49–6.44 (m, 2H), 3.67 (s, 2H); ^13^C {^1^H} NMR (101 MHz, CDCl_3_): δ 146.1, 137.9, 117.3, 79.4; MS (EI) *m*/*z* 219 (M^+^, 100), 191 (3), 127 (12), 92 (43), 65 (31).

### *N*-(4-Iodophenyl)­acetamide (**3d**)^8b^

The reaction was performed as described in the general procedure using *N*-phenylacetamide (68.0 mg, 0.500 mmol, 1 equiv), *N*-iodosaccharin (**2**) (201 mg, 0.650 mmol, 1.3 equiv), iron trichloride (4.10 mg, 0.0250 mmol, 0.05 equiv), and acetonitrile (1.0 mL) for 0.5 h. *N*-(4-Iodophenyl)­acetamide (**3d**) (89.5 mg, 68%) was obtained as a white solid after purification by column chromatography with 50% ethyl acetate in hexane. mp 174–176 °C (lit.^8b^ 178–180 °C); ^1^H NMR (400 MHz, CD_3_OD): δ 7.60 (d, *J* = 8.0 Hz, 2H), 7.35 (d, *J* = 8.0 Hz, 2H), 2.09 (s, 3H); ^13^C­{^1^H} NMR (101 MHz, CD_3_OD): δ 170.3, 138.5, 137.5, 121.5, 86.2, 22.5; MS (EI) *m*/*z* 261 (M^+^, 100), 219 (93), 92 (26), 65 (12).

### Benzyl (4-Iodophenyl)­carbamate (**3e**)^31^

The reaction was performed as described in the general procedure using benzyl phenylcarbamate (114 mg, 0.500 mmol, 1 equiv), *N*-iodosaccharin (**2**) (201 mg, 0.650 mmol, 1.3 equiv), iron trichloride (4.10 mg, 0.0250 mmol, 0.05 equiv), and acetonitrile (1.0 mL) for 0.5 h. Benzyl (4-iodophenyl)­carbamate (**3e**) (156 mg, 88%) was obtained as an off-white solid after purification by column chromatography with 20% ethyl acetate in hexane. The data were consistent with the literature.^31^ mp 128–130 °C; ^1^H NMR (400 MHz, CDCl_3_): δ 7.59 (d, *J* = 8.4 Hz, 2H), 7.43–7.33 (m, 5H), 7.17 (d, *J* = 8.4 Hz, 2H), 6.65 (br s, 1H), 5.19 (s, 2H); ^13^C­{^1^H} NMR (101 MHz, CDCl_3_): δ 153.2, 138.1, 137.7, 135.9, 128.8, 128.6, 128.5, 120.7, 86.5, 67.4; MS (APCI) *m*/*z* 354 [(M + H)^+^, 100].

### *N*-(4-Iodophenyl)-4-methylbenzenesulfonamide (**3f**)^32^

The reaction was performed as described in the general procedure using 4-methyl-*N*-phenylbenzenesulfonamide (124 mg, 0.500 mmol, 1 equiv), *N*-iodosaccharin (**2**) (201 mg, 0.650 mmol, 1.3 equiv), iron trichloride (4.10 mg, 0.0250 mmol, 0.05 equiv), and acetonitrile (1.0 mL) for 0.5 h. *N*-(4-Iodophenyl)-4-methylbenzenesulfonamide (**3f**) (171 mg, 88%) was obtained as an off-white solid after purification by column chromatography with 20% ethyl acetate in hexane. mp 97–98 °C (lit.^32^ 96–98 °C); ^1^H NMR (400 MHz, CDCl_3_): δ 7.70–7.62 (m, 2H), 7.55–7.49 (m, 2H), 7.24 (d, *J* = 7.7 Hz, 2H), 6.85 (dt, *J* = 7.0, 2.1 Hz, 2H), 2.38 (s, 3H); ^13^C­{^1^H} NMR (101 MHz, CDCl_3_): δ 144.4, 138.4, 136.7, 135.7, 130.0, 127.4, 123.1, 89.2, 21.7; MS (APCI) *m*/*z* 374 [(M + H)^+^, 100].

### 4-Iodo-1-methoxy-2-methylbenzene (**3g**)^33^

The reaction was performed as described in the general procedure using 1-methoxy-2-methylbenzene (62.0 μL, 0.500 mmol, 1 equiv), *N*-iodosaccharin (**2**) (201 mg, 0.650 mmol, 1.3 equiv), iron trichloride (4.10 mg, 0.0250 mmol, 0.05 equiv), and acetonitrile (1.0 mL) for 0.5 h. 4-Iodo-1-methoxy-2-methylbenzene (**3g**) (90.0 mg, 73%) was obtained as a white solid after purification by column chromatography with 10% ethyl acetate in hexane. mp 78–80 °C (lit.^33^ 75–76 °C); ^1^H NMR (400 MHz, CDCl_3_): δ 7.47–7.41 (m, 2H), 6.58 (d, *J* = 8.2 Hz, 1H), 3.80 (s, 3H), 2.17 (s, 3H); ^13^C­{^1^H} NMR (101 MHz, CDCl_3_): δ 157.8, 139.1, 135.6, 129.6, 112.3, 82.6, 55.5, 16.0; MS (APCI) *m*/*z* 248 (M^+^, 100).

### *N*-(4-Iodo-2-methylphenyl)­acetamide (**3h**)^34^

The reaction was performed as described in the general procedure using *N*-(*o*-tolyl)­acetamide (75.0 mg, 0.500 mmol, 1 equiv), *N*-iodosaccharin (**2**) (201 mg, 0.650 mmol, 1.3 equiv), iron trichloride (4.10 mg, 0.0250 mmol, 0.05 equiv), and acetonitrile (1.0 mL) for 0.5 h. *N*-(4-Iodo-2-methylphenyl)­acetamide (**3h**) (127 mg, 92%) was obtained as an off-white solid after purification by column chromatography with 50% ethyl acetate in hexane. The data were consistent with the literature.^34^ mp 164–167 °C; ^1^H NMR (400 MHz, CD_3_OD): δ 7.62–7.58 (m, 1H), 7.50 (dd, *J* = 8.3, 2.1 Hz, 1H), 7.15 (d, *J* = 8.3 Hz, 1H), 2.21 (s, 3H), 2.14 (s, 3H); ^13^C­{^1^H} NMR (101 MHz, CD_3_OD): δ 172.1, 140.4, 137.0, 136.6, 136.4, 128.6, 91.1, 23.1, 17.7; MS (APCI) *m*/*z* 276 [(M + H)^+^, 100].

### 5-Iodo-2-methoxy-1,3-dimethylbenzene (**3i**)^35^

The reaction was performed as described in the general procedure using 2-methoxy-1,3-dimethylbenzene (71.0 μL, 0.500 mmol, 1 equiv), *N*-iodosaccharin (**2**) (201 mg, 0.650 mmol, 1.3 equiv), iron trichloride (4.10 mg, 0.0250 mmol, 0.05 equiv), and acetonitrile (1.0 mL) for 0.5 h. 5-Iodo-2-methoxy-1,3-dimethylbenzene (**3i**) (114 mg, 87%) was obtained as a colorless liquid after purification by column chromatography with 10% ethyl acetate in hexane. The data were consistent with the literature.^35^ ^1^H NMR (400 MHz, CDCl_3_): δ 7.35 (s, 2H), 3.70 (s, 3H), 2.24 (s, 6H); ^13^C­{^1^H} NMR (101 MHz, CDCl_3_): δ 157.1, 137.6, 133.6, 87.7, 59.8, 15.8; MS (APCI) *m*/*z* 262 (M^+^, 100).

### 2-Iodo-1,3,5-trimethoxybenzene (**3j**)^36^

The reaction was performed as described in the general procedure using 1,3,5-trimethoxybenzene (84.1 mg, 0.500 mmol, 1 equiv), *N*-iodosaccharin (**2**) (201 mg, 0.650 mmol, 1.3 equiv), iron trichloride (4.10 mg, 0.0250 mmol, 0.05 equiv), and acetonitrile (1.0 mL) for 0.5 h. The reaction mixture was stirred at room temperature for 0.5 h. 2-Iodo-1,3,5-trimethoxybenzene (**3j**) (147 mg, 95%) was obtained as a light-yellow oil after purification by column chromatography with 5% ethyl acetate in hexane. The data were consistent with the literature.^36^ ^1^H NMR (400 MHz, CDCl_3_): δ 6.14 (s, 2H), 3.86 (s, 6H), 3.82 (s, 3H); ^13^C­{^1^H} NMR (101 MHz, CDCl_3_): δ 162.3, 160.0, 91.4, 66.8, 56.6, 55.7; MS (APCI) *m*/*z* 295 [(M + H)^+^, 100].

### 5-Iodo-2-methoxybenzaldehyde (**3k**)^37^

The reaction was performed as described in the general procedure using 2-methoxybenzaldehyde (68.0 mg, 0.500 mmol, 1 equiv), *N*-iodosaccharin (**2**) (201 mg, 0.650 mmol, 1.3 equiv), iron triflimide (22.0 μL, 0.0750 mmol, 0.15 equiv), and acetonitrile (1.0 mL) for 4.5 h. 5-Iodo-2,3-dimethoxybenzaldehyde (**3k**) (102 mg, 78%) was obtained as a white solid after purification by column chromatography with 10% ethyl acetate in hexane. mp 141–142 °C (lit.^37^ 142–143 °C); ^1^H NMR (400 MHz, CDCl_3_): δ 10.33 (s, 1H), 8.08 (d, *J* = 2.4 Hz, 1H), 7.80 (dd, *J* = 8.8, 2.4 Hz, 1H), 6.78 (d, *J* = 8.8 Hz, 1H), 3.91 (s, 3H); ^13^C­{^1^H} NMR (101 MHz, CDCl_3_): δ 188.3, 161.5, 144.1, 137.1, 126.6, 114.2, 83.0, 55.9; MS (APCI) *m*/*z* 263 [(M + H)^+^, 100].

### 2,4-Dimethoxy-5-iodobenzaldehyde (**3l**)^38^

The reaction was performed as described in the general procedure using 2,4-dimethoxybenzaldehyde (83.0 mg, 0.500 mmol, 1 equiv), *N*-iodosaccharin (**2**) (201 mg, 0.650 mmol, 1.3 equiv), iron trichloride (4.10 mg, 0.0250 mmol, 0.05 equiv), and acetonitrile (1.0 mL) for 0.5 h. 2,4-Dimethoxy-5-iodobenzaldehyde (**3L**) (130 mg, 89%) was obtained as a white solid after purification by column chromatography with 50% ethyl acetate in hexane. mp 171–172 °C (lit.^38^ 170–172 °C); ^1^H NMR (400 MHz, CDCl_3_): δ 10.18 (s, 1H), 8.20 (s, 1H), 6.38 (s, 1H), 3.96 (s, 3H), 3.94 (s, 3H); ^13^C­{^1^H} NMR (101 MHz, CDCl_3_): δ 187.0, 164.1, 163.8, 139.3, 120.4, 94.8, 75.6, 56.7, 55.8; MS (EI) *m*/*z* 292 (M^+^, 100), 246 (15), 148 (13), 84 (29), 49 (27).

### 1-Iodo-4-methoxynaphthalene (**3m**)^18^

The reaction was performed as described in the general procedure using 1-methoxynaphthalene (73.0 μL, 0.500 mmol, 1 equiv), *N*-iodosaccharin (**2**) (201 mg, 0.650 mmol, 1.3 equiv), iron trichloride (4.10 mg, 0.0250 mmol, 0.05 equiv), and acetonitrile (1.0 mL) for 0.5 h. 1-Iodo-4-methoxynaphthalene (**3m**) (135 mg, 95%) was obtained as a colorless liquid after purification by column chromatography with 5% ethyl acetate in hexane. The data were consistent with the literature.^18^ ^1^H NMR (400 MHz, CDCl_3_): δ 8.24 (ddd, *J* = 8.3, 1.3, 0.6 Hz, 1H), 8.04 (ddd, *J* = 8.4, 1.1, 0.6, 1H), 7.95 (d, *J* = 8.1 Hz, 1H), 7.59 (ddd, *J* = 8.4, 6.8, 1.3 Hz, 1H), 7.53 (ddd, *J* = 8.3, 6.8, 1.1 Hz, 1H), 6.59 (d, *J* = 8.1 Hz, 1H), 3.98 (s, 3H); ^13^C­{^1^H} NMR (101 MHz, CDCl_3_): δ 156.3, 136.9, 134.7, 131.8, 128.2, 126.7, 126.0, 122.5, 105.6, 88.2, 55.7; MS (EI) *m*/*z* 284 (M^+^, 100), 269 (35), 241 (31), 114 (30).

### 4-Iodo-3,5-dimethylphenol (**3n**)^39^

The reaction was performed as described in the general procedure using 3,5-dimethylphenol (61.0 mg, 0.500 mmol, 1 equiv), *N*-iodosaccharin (**2**) (201 mg, 0.650 mmol, 1.3 equiv), iron trichloride (4.10 mg, 0.0250 mmol, 0.05 equiv), and acetonitrile (1.0 mL). The reaction mixture was stirred at room temperature for 0.5 h. 4-Iodo-3,5-dimethylphenol (**3n**) (82.0 mg, 66%) was obtained as a white solid after purification by column chromatography with 10% ethyl acetate in hexane. mp 129–131 °C (lit.^39^ 130–132 °C); ^1^H NMR (400 MHz, CDCl_3_): δ 6.61 (s, 2H), 4.59 (s, 1H), 2.42 (s, 6H); ^13^C­{^1^H} NMR (101 MHz, CDCl_3_): δ 155.2, 143.4, 114.4, 97.2, 29.7; MS (APCI) *m*/*z* 248 (M^+^, 100).

### 2-Amino-5-iodobenzonitrile (**3o**)^40^

The reaction was performed as described in the general procedure using 2-aminobenzonitrile (59.0 mg, 0.500 mmol, 1 equiv), *N*-iodosaccharin (**2**) (201 mg, 0.650 mmol, 1.3 equiv), iron trichloride (4.10 mg, 0.0250 mmol, 0.05 equiv), and acetonitrile (1.0 mL) for 0.5 h. 2-Amino-5-iodobenzonitrile (**3o**) (76.0 mg, 62%) was obtained as an off-white solid after purification by column chromatography with 40% ethyl acetate in hexane. mp 84–86 °C (lit.^40^ 86 °C); ^1^H NMR (400 MHz, CDCl_3_): δ 7.65 (d, *J* = 1.9 Hz, 1H), 7.56 (dt, *J* = 8.7, 1.9 Hz, 1H), 6.53 (d, *J* = 8.7 Hz, 1H), 4.45 (br s, 2H); ^13^C­{^1^H} NMR (101 MHz, CDCl_3_): δ 149.2, 142.7, 140.1, 117.2, 116.2, 98.5, 77.4; MS (APCI) *m*/*z* 245 (M + H^+^, 100).

### Methyl 3-Amino-6-iodobenzoate (**3p**)^41^

The reaction was performed as described in the general procedure using methyl 3-aminobenzoate (68.0 mg, 0.500 mmol, 1 equiv), *N*-iodosaccharin (**2**) (201 mg, 0.650 mmol, 1.3 equiv), iron trichloride (4.10 mg, 0.0250 mmol, 0.05 equiv), and acetonitrile (1.0 mL) for 0.5 h. Methyl 3-amino-6-iodobenzoate (**3p**) (125 mg, 90%) was obtained as a light brown oil after purification by column chromatography with 25% ethyl acetate in hexane. The data were consistent with the literature.^41^ ^1^H NMR (400 MHz, CDCl_3_): δ 7.67 (d, *J* = 8.4 Hz, 1H), 7.14 (d, *J* = 2.7 Hz, 1H), 6.51 (dd, *J* = 8.4, 2.7 Hz, 1H), 3.90 (s, 3H), 3.79 (br s, 2H); ^13^C­{^1^H} NMR (101 MHz, CDCl_3_): δ 167.1, 146.4, 141.6, 135.6, 119.6, 117.5, 78.7, 52.4; MS (EI) *m*/*z* 277 (M^+^, 100), 246 (33), 218 (16), 133 (19), 91 (13), 77 (9).

### 2-Iodo-4-nitroaniline (**3q**)^42^

The reaction was performed as described in the general procedure using 4-nitroaniline (68.0 mg, 0.500 mmol, 1 equiv), *N*-iodosaccharin (**2**) (201 mg, 0.650 mmol, 1.3 equiv), iron trichloride (4.10 mg, 0.0250 mmol, 0.05 equiv), and acetonitrile (1.0 mL) for 0.5 h. 2-Iodo-4-nitroaniline (**3q**) (95.0 mg, 72%) was obtained as a yellow solid after purification by column chromatography with 30% ethyl acetate in hexane. mp 99–100 °C (lit.^42^ 103–104 °C); ^1^H NMR (400 MHz, CDCl_3_): δ 8.57 (d, *J* = 2.5 Hz, 1H), 8.06 (dd, *J* = 9.0, 2.5 Hz, 1H), 6.70 (d, *J* = 9.0 Hz, 1H), 4.83 (br s, 2H); ^13^C­{^1^H} NMR (101 MHz, CDCl_3_): δ 152.3, 139.3, 135.5, 125.7, 112.3, 80.6; MS (EI) *m*/*z* 264 (M^+^, 100), 234 (38), 218 (11), 127 (5), 91 (31).

### 4-Amino-3-iodoacetophenone (**3r**)^43^

The reaction was performed as described in the general procedure using 4-aminoacetophenone (68.0 mg, 0.500 mmol, 1 equiv), *N*-iodosaccharin (**2**) (201 mg, 0.650 mmol, 1.3 equiv), iron trichloride (4.10 mg, 0.0250 mmol, 0.05 equiv), and acetonitrile (1.0 mL) for 0.5 h. 4-Amino-3-iodoacetophenone (**3r**) (115 mg, 88%) was obtained as a yellow oil after the purification by column chromatography with 20% ethyl acetate in hexane. The data were consistent with the literature.^43^ ^1^H NMR (400 MHz, CDCl_3_): δ 8.27 (d, *J* = 2.0 Hz, 1H), 7.76 (dd, *J* = 8.4, 2.0 Hz, 1H), 6.71 (d, *J* = 8.4 Hz, 1H), 4.61 (br s, 2H), 2.49 (s, 3H); ^13^C {^1^H} NMR (101 MHz, CDCl_3_): δ 195.2, 150.9, 140.3, 130.3, 129.3, 113.1, 82.6, 26.0; MS (EI) *m*/*z* 262 (M + H^+^, 100), 136 (48), 85 (10), 69 (18).

### 2-Iodo-1,3,5-trimethylbenzene (**3s**)^44^

The reaction was performed as described in the general procedure using 1,3,5-trimethylbenzene (60.0 mg, 0.500 mmol, 1 equiv), *N*-iodosaccharin (**2**) (201 mg, 0.650 mmol, 1.3 equiv), iron trichloride (4.10 mg, 0.0250 mmol, 0.05 equiv), and acetonitrile (1.0 mL) for 0.5 h. 2-Iodo-1,3,5-trimethylbenzene (**3s**) (100 mg, 82%) was obtained as a colorless liquid after purification by column chromatography with 10% ethyl acetate in hexane. The data were consistent with the literature.^44^ ^1^H NMR (400 MHz, CDCl_3_): δ 6.89 (s, 2H), 2.44 (s, 6H), 2.24 (s, 3H); ^13^C­{^1^H} NMR (101 MHz, CDCl_3_): δ 141.9, 137.5, 128.1, 104.4, 29.6, 20.8; MS (APCI) *m*/*z* 247 [(M + H)^+^, 100].

### 2,4-Dimethyl-1-iodobenzene (**3t**)^45^

The reaction was performed as described in the general procedure using *m*-xylene (53.0 mg, 0.500 mmol, 1 equiv), *N*-iodosaccharin (**2**) (201 mg, 0.650 mmol, 1.3 equiv), iron trichloride (4.10 mg, 0.0250 mmol, 0.05 equiv), and acetonitrile (1.0 mL) for 0.5 h. 2,4-Dimethyl-1-iodobenzene (**3t**) (72.0 mg, 62%) was obtained as a colorless liquid after purification by column chromatography with 10% ethyl acetate in hexane. The data were consistent with the literature.^45^ ^1^H NMR (400 MHz, CDCl_3_): δ 7.66 (d, *J* = 8.0 Hz, 1H), 7.07 (d, *J* = 2.3 Hz, 1H), 6.70 (dd, *J* = 8.0, 2.3 Hz, 1H), 2.39 (s, 3H), 2.27 (s, 3H); ^13^C­{^1^H} NMR (101 MHz, CDCl_3_): δ 141.2, 138.8, 138.2, 130.9, 128.5, 97.1, 28.1, 21.0; MS (APCI) *m*/*z* 232 (M^+^, 100).

### 5-Iodo-2,3-dihydrobenzofuran (**3u**)^46^

The reaction was performed as described in the general procedure using 2,3-dihydrobenzofuran (56.0 μL mg, 0.500 mmol, 1 equiv), *N*-iodosaccharin (**2**) (201 mg, 0.650 mmol, 1.3 equiv), iron trichloride (4.10 mg, 0.0250 mmol, 0.05 equiv), and acetonitrile (1.0 mL) for 1 h. 5-Iodo-2,3-dihydrobenzofuran (**3u**) (102 mg, 83%) was obtained as a white solid after purification by column chromatography with 10% ethyl acetate in hexane. mp 61–63 °C (lit.^46^ 64–65 °C); ^1^H NMR (400 MHz, CDCl_3_): δ 7.47 (dt, *J* = 1.9, 1.1 Hz, 1H), 7.38 (ddt, *J* = 8.4, 1.9, 0.7 Hz, 1H), 6.57 (d, *J* = 8.4 Hz, 1H), 4.56 (t, *J* = 8.7 Hz, 2H), 3.20 (br t, *J* = 8.7 Hz, 2H); ^13^C­{^1^H} NMR (101 MHz, CDCl_3_): δ 160.1, 136.7, 133.7, 130.1, 111.7, 81.6, 71.5, 29.5; MS (EI) *m*/*z* 246 (M^+^, 89), 232 (10), 117 (20), 91 (41), 84 (39), 44 (100).

### 1-Iodonaphthalen-2-ol (**3w**)^36^

The reaction was performed as described in the general procedure using naphthalen-2-ol (63.0 mg, 0.500 mmol, 1 equiv), *N*-iodosaccharin (**2**) (201 mg, 0.650 mmol, 1.3 equiv), silver bis­(trifluoromethanesulfonyl)­imide (9.70 mg, 0.0250 mmol, 0.05 equiv), and acetonitrile (1.0 mL). The reaction mixture was stirred at 0 °C for 3 h. 1-Iodonaphthalen-2-ol (**3w**) (129 mg, 95%) was isolated as a white solid after purification by column chromatography with 10% ethyl acetate in hexane. The data were consistent with the literature.^36^ mp 87–89 °C; ^1^H NMR (400 MHz, CDCl_3_): δ 7.93 (d, *J* = 8.5 Hz, 1H), 7.77–7.71 (m, 2H), 7.55 (ddd, *J* = 8.4, 6.9, 1.3 Hz, 1H), 7.39 (ddd, *J* = 8.4, 6.9, 1.3 Hz, 1H), 7.26 (d, *J* = 8.5 Hz, 1H), 5.78 (br s, 1H); ^13^C NMR (101 MHz, CDCl_3_): δ 153.9, 134.9, 130.8, 130.4, 129.8, 128.4, 128.4, 124.3, 116.6, 86.4; MS (APCI) *m*/*z* 271 [(M + H)^+^, 100].

### Methyl 4-Iodo-1*H*-pyrrole-2-carboxylate (**3x**)^16^

The reaction was performed as described in the general procedure using methyl 1*H*-pyrrole-2-carboxylate (63.0 mg, 0.500 mmol, 1 equiv), *N*-iodosaccharin (**2**) (201 mg, 0.650 mmol, 1.3 equiv), silver bis­(trifluoromethanesulfonyl)­imide (9.70 mg, 0.0250 mmol, 0.05 equiv), and acetonitrile (1.0 mL) for 3 h. Methyl 4-iodo-1*H*-pyrrole-2-carboxylate (**3x**) (110 mg, 87%) was isolated as a white solid. mp 87–89 °C (lit.^16^ 87–90 °C); ^1^H NMR (400 MHz, CDCl_3_): δ 9.68 (br s, 1H), 7.01 (dd, *J* = 2.7, 1.5 Hz, 1H), 6.98 (dd, *J* = 2.7, 1.5 Hz, 1H), 3.86 (s, 3H); ^13^C­{^1^H} ^13^C NMR (101 MHz, CDCl_3_): δ 160.8, 127.9, 124.3, 121.9, 61.7, 51.9; MS (EI) *m*/*z* 251 (M^+^, 100), 219 (70), 192 (11), 124 (5), 93 (6), 65 (8), 44 (10).

### 3-Iodo-1*H*-indole (**3y**)^47^

The reaction was performed as described in the general procedure using indole (59 mg, 0.500 mmol, 1 equiv), *N*-iodosaccharin (**2**) (201 mg, 0.65 mmol, 1.3 equiv), silver bis­(trifluoromethanesulfonyl)­imide (9.7 mg, 0.025 mmol, 0.05 equiv), and acetonitrile (1.0 mL) for 2 h. 3-Iodo-1*H*-indole (**3y**) (53.0 mg, 44%) was isolated as a white solid. mp 67–68 °C (lit.^47^ 66–68 °C); ^1^H NMR (400 MHz, CDCl_3_): δ 8.27 (br s, 1H), 7.48–7.44 (m, 1H), 7.37–7.32 (m, 1H), 7.28–7.16 (m, 3H); ^13^C­{^1^H} ^13^C NMR (101 MHz, CDCl_3_): δ 135.7, 129.9, 128.5, 123.3, 121.2, 121.0, 111.4, 57.7; MS (APCI) *m*/*z* 243 (M^+^, 100).

### Methyl 5-(2′,5′-Dimethylphenoxy)-2,2-dimethylpentanoate (**4a**, Gemfibrozil-methyl Ester)^48^

Gemfibrozil (501 mg, 2.00 mmol, 1 equiv) was dissolved in methanol (10 mL) and cooled to 0 °C. Thionyl chloride (0.250 mL, 3.40 mmol, 1.7 equiv) was added to the flask dropwise at 0 °C and stirred for 0.25 h. The reaction mixture was allowed to warm to room temperature, and the reaction mixture was then heated under reflux for 2.5 h. The reaction mixture was concentrated in vacuo. The reaction mixture was redissolved in ethyl acetate (20 mL) and washed with a saturated sodium carbonate solution (20 mL). The organic layer was dried (MgSO_4_) and concentrated in vacuo to give methyl 5-(2′,5′-dimethylphenoxy)-2,2-dimethylpentanoate (**4a**) (486 mg, 92%) as a colorless liquid. The data were consistent with the literature.^48^ ^1^H NMR (400 MHz, CDCl_3_): δ 7.00 (d, *J* = 7.4 Hz, 1H), 6.66 (d, *J* = 7.4 Hz, 1H), 6.60 (s, 1H), 3.95–3.88 (m, 2H), 3.67 (s, 3H), 2.31 (s, 3H), 2.18 (s, 3H), 1.78–1.67 (m, 4H), 1.22 (s, 6H); ^13^C­{^1^H} NMR (101 MHz, CDCl_3_): δ 178.5, 157.1, 136.6, 130.4, 123.7, 120.8, 112.1, 68.0, 51.9, 42.3, 37.3, 25.34, 25.33, 21.6, 15.9; MS (APCI) *m*/*z* 265 [(M + H)^+^, 100].

### Methyl 5-(4′-Iodo-2′,5′-dimethylphenoxy)-2,2-dimethylpentanoate (**5a**)^49^

The reaction was performed as described in the general procedure using methyl 5-(2′,5′-dimethylphenoxy)-2,2-dimethylpentanoate (132 mg, 0.500 mmol, 1 equiv), *N*-iodosaccharin (**2**) (201 mg, 0.650 mmol, 1.3 equiv), iron trichloride (4.10 mg, 0.0250 mmol, 0.05 equiv), and acetonitrile (1.0 mL) for 0.1 h. Methyl 5-(4′-iodo-2′,5′-dimethylphenoxy)-2,2-dimethylpentanoate (**5a**) (184 mg, 94%) was obtained as a colorless liquid after purification by column chromatography with 20% ethyl acetate in hexane. The data were consistent with the literature.^49^ ^1^H NMR (400 MHz, CDCl_3_): δ 7.51 (s, 1H), 6.67 (s, 1H), 3.89 (t, *J* = 5.5 Hz, 2H), 3.66 (s, 3H), 2.37 (s, 3H), 2.13 (s, 3H), 1.74–1.69 (m, 4H), 1.22 (s, 6H); ^13^C­{^1^H} NMR (101 MHz, CDCl_3_): δ 178.4, 157.5, 140.1, 139.5, 126.7, 112.9, 89.1, 68.2, 51.9, 42.2, 37.2, 28.1, 25.3, 25.2, 15.4; MS (APCI) *m*/*z* 391 [(M + H)^+^, 100].

### Methyl 2-{2-[(2′,6′-Dichlorophenyl)­amino]­phenyl}­acetate (4b, Diclofenac Methyl Ester)^50^

To a solution of diclofenac (0.50 g, 1.7 mmol) in methanol (10 mL), a few drops of concentrated sulfuric acid were added. The mixture was stirred at reflux for 18 h. The reaction mixture was concentrated under reduced pressure and redissolved in dichloromethane (20 mL). The organic layer was washed with a saturated solution of sodium carbonate (20 mL) and brine (20 mL), dried over MgSO_4_, and concentrated in vacuo. Purification by silica gel column chromatography using 30% ethyl acetate in hexane gave methyl 2-{2-[(2′,6′-dichlorophenyl)­amino]­phenyl}­acetate (**4b**) (0.31 g, 59%) as a white solid. The data were consistent with the literature.^50^ mp 97–100 °C; ^1^H NMR (400 MHz, CDCl_3_): δ 7.35 (d, *J* = 8.0 Hz, 2H), 7.23 (dd, *J* = 7.5, 1.6 Hz, 1H), 7.14 (td, *J* = 7.7, 1.6 Hz, 1H), 7.02–6.92 (m, 3H), 6.55 (d, *J* = 7.7 Hz, 1H), 3.82 (s, 2H), 3.75 (s, 3H); ^13^C­{^1^H} NMR (101 MHz, CDCl_3_): δ 172.6, 142.7, 137.8, 130.9, 129.5, 128.9, 128.0, 124.09, 124.06, 122.0, 118.1, 52.5, 38.5; MS (APCI) *m*/*z* 310 [(M + H)^+^, 100].

### Methyl 2-{2-[(2′,6′-Dichlorophenyl)­amino]-5-iodophenyl}­acetate (**5b**)^17^

The reaction was performed as described in the general procedure using methyl 2-{2-[(2′,6′-dichlorophenyl)­amino]­phenyl}­acetate (155 mg, 0.500 mmol, 1 equiv), *N*-iodosaccharin (**2**) (201 mg, 0.650 mmol, 1.3 equiv), iron trichloride (4.10 mg, 0.0250 mmol, 0.05 equiv), and acetonitrile (1.0 mL) for 0.5 h. Methyl 2-{2-[(2′,6′-dichlorophenyl)­amino]-5-iodophenyl}­acetate (**5b**) (201 mg, 92%) was obtained as a white solid after purification by column chromatography with 10% ethyl acetate in hexane. mp 117–119 °C (lit.^17^ 118–120 °C); ^1^H NMR (400 MHz, CDCl_3_): δ 7.54 (d, *J* = 2.1 Hz, 1H), 7.39 (dd, *J* = 8.5, 2.1 Hz, 1H), 7.35 (d, *J* = 8.1 Hz, 2H), 7.01 (t, *J* = 8.1 Hz, 1H), 6.95 (br s, 1H), 6.28 (d, *J* = 8.5 Hz, 1H), 3.76 (s, 3H), 3.74 (s, 2H); ^13^C­{^1^H} NMR (101 MHz, CDCl_3_): δ 172.3, 142.8, 139.4, 137.2, 136.9, 129.9, 129.1, 126.3, 124.8, 120.0, 84.1, 52.8, 38.2; MS (APCI) *m*/*z* 436 [(M + H)^+^, 100].

### Methyl (2*S*)-2′-(6′-Methoxynaphthalen-2′-yl)-2-methylethanoate (**4c**)^48^

Naproxen (461 mg, 2.00 mmol, 1 equiv) was dissolved in methanol (10 mL) and cooled to 0 °C. Thionyl chloride (0.250 mL, 3.40 mmol, 1.7 equiv) was added, and the mixture was stirred for 0.25 h. The reaction mixture was warmed to room temperature and then heated under reflux for 2.5 h. The reaction mixture was concentrated in vacuo. The resulting mixture was dissolved in ethyl acetate (20 mL) and washed with a saturated sodium carbonate solution (20 mL). The organic layer was dried (MgSO_4_) and concentrated in vacuo to give methyl (2*S*)-2′-(6′-methoxynaphthalen-2′-yl)-2-methylethanoate (**4c**) (455 mg, 93%) as a white solid. The data were consistent with the literature.^48^ mp 88–90 °C; [α]_D_^18^ +74.0 (*c* 0.1, CHCl_3_); ^1^H NMR (400 MHz, CDCl_3_): δ 7.71 (d, *J* = 8.5 Hz, 2H), 7.68–7.65 (m, 1H), 7.40 (dd, *J* = 8.5, 1.8 Hz, 1H), 7.19–7.08 (m, 2H), 3.91 (s, 3H), 3.86 (q, *J* = 7.2 Hz, 1H), 3.67 (s, 3H), 1.58 (d, *J* = 7.2 Hz, 3H); ^13^C­{^1^H} NMR (101 MHz, CDCl_3_): δ 175.3, 157.8, 135.8, 133.8, 129.4, 129.1, 127.3, 126.3, 126.1, 119.1, 105.7, 55.4, 52.2, 45.5, 18.7; MS (APCI) *m*/*z* 245 [(M + H)^+^, 100].

### Methyl (2*S*)-2′-(5′-Iodo-6′-methoxynaphthalen-2′-yl)-2-methylethanoate (**5c**)^51^

The reaction was performed as described in the general procedure using methyl (2*S*)-2′-(6′-methoxynaphthalen-2′-yl)-2-methylethanoate (122 mg, 0.500 mmol, 1 equiv), *N*-iodosaccharin (**2**) (201 mg, 0.65 mmol, 1.3 equiv), iron trichloride (4.1 mg, 0.025 mmol, 0.05 equiv), and acetonitrile (1.0 mL) for 0.5 h. Methyl (2*S*)-2′-(5′-iodo-6′-methoxynaphthalen-2′-yl)-2-methylethanoate (**5c**) (166 mg, 90%) was obtained as a white solid after purification by column chromatography with 20% ethyl acetate in hexane. The data were consistent with the literature.^51^ mp 59–61 °C; [α]_D_^17^ +47.2 (*c* 0.1, CHCl_3_); ^1^H NMR (400 MHz, CDCl_3_): δ 8.11 (d, *J* = 8.9 Hz, 1H), 7.79 (dd, *J* = 8.9, 3.6 Hz, 1H), 7.66–7.63 (m, 1H), 7.49 (dd, *J* = 8.9, 1.8 Hz, 1H), 7.20 (dd, *J* = 8.9, 5.1 Hz, 1H), 4.01 (d, *J* = 5.1 Hz, 3H), 3.89 (q, *J* = 7.2 Hz, 1H), 3.67 (s, 3H), 1.59 (d, *J* = 7.2 Hz, 3H); ^13^C­{^1^H} NMR (101 MHz, CDCl_3_): δ 175.0, 156.8, 136.6, 135.0, 131.8, 130.4, 130.0, 128.1, 126.5, 113.3, 87.5, 57.4, 52.3, 45.2, 18.6; MS (APCI) *m*/*z* 370 (M^+^, 100).

### 5-[(4′-Iodo-3′,5′-dimethylphenoxy]­methyl)­oxazolidin-2-one (**5d**)^17^

The reaction was performed as described in the general procedure using metaxalone (111 mg, 0.500 mmol, 1 equiv), *N*-iodosaccharin (**2**) (201 mg, 0.650 mmol, 1.3 equiv), iron trichloride (4.10 mg, 0.0250 mmol, 0.05 equiv), and acetonitrile (1.0 mL) for 0.25 h. 5-[(4′-Iodo-3′,5′-dimethylphenoxy)­methyl]­oxazolidin-2-one (151 mg, 87%) was obtained as a white solid after purification by column chromatography with 5% methanol in dichloromethane. mp 148–150 °C (lit.^17^ 149–151 °C); ^1^H NMR (400 MHz, DMSO-*d*_6_): δ 7.58 (br s, 1H), 6.83 (s, 2H), 4.92–4.83 (m, 1H), 4.14 (dd, *J* = 11.2, 3.6 Hz, 1H), 4.07 (dd, *J* = 11.2, 6.0 Hz, 1H), 3.60 (t, *J* = 8.9 Hz, 1H), 3.29 (dd, *J* = 8.9, 6.8 Hz, 1H), 2.37 (s, 6H); ^13^C­{^1^H} NMR (101 MHz, DMSO-*d*_6_): δ 159.1, 158.3, 142.9, 114.3, 97.9, 73.9, 69.1, 41.9, 29.5; MS (APCI) *m*/*z* 348 [(M + H)^+^, 100].

### 4-Iodo-9-methoxy-7*H*-furo­[3,2-*g*]­chromen-7-one (**5e**)^17^

The reaction was performed as described in the general procedure using methoxsalen (108 mg, 0.500 mmol, 1 equiv), *N*-iodosaccharin (**2**) (201 mg, 0.650 mmol, 1.3 equiv), and acetonitrile (1.0 mL) for 0.5 h. 4-Iodo-9-methoxy-7H-furo­[3,2-*g*]­chromen-7-one (111 mg, 65%) was obtained as a white solid after purification by column chromatography with 40% ethyl acetate in hexane. mp 189–191 °C (lit.^17^ 188–190 °C); ^1^H NMR (400 MHz, CDCl_3_): δ 8.01 (d, *J* = 9.8 Hz, 1H), 7.74 (d, *J* = 2.3 Hz, 1H), 6.79 (d, *J* = 2.3 Hz, 1H), 6.42 (d, *J* = 9.8 Hz, 1H), 4.29 (s, 3H); ^13^C­{^1^H} NMR (101 MHz, CDCl_3_): δ 160.0, 147.1, 146.6, 145.5, 143.4, 133.2, 132.5, 118.5, 116.2, 110.7, 79.9, 61.4; MS (APCI) *m*/*z* 343 [(M + H)^+^, 100].

### (8*R*,9*S*,13*S*,14*S*,17*S*)-3,17-Dimethoxy-13-methyl-7,8,9,11,12,13,14,15,16,17-decahydro-6*H*-cyclopenta­[*a*]­phenanthrene (**4f**)^25c^

β-Estradiol (0.750 g, 2.75 mmol, 1 equiv) was dissolved in THF (15 mL) and cooled to 0 °C. Sodium hydride (1.07 g, 11.0 mmol, 4 equiv) was added, and the reaction mixture was stirred for 0.3 h. Iodomethane (1.00 mL, 16.5 mmol, 6 equiv) was added dropwise. The reaction mixture was warmed to room temperature and stirred for 18 h. The reaction was quenched with the addition of a saturated ammonium chloride solution (20 mL). The reaction mixture was extracted with ethyl acetate (3 × 10 mL). The combined organic layers were washed with an aqueous sodium hydrogen carbonate solution (20 mL) and dried (MgSO_4_). The solvent was concentrated to give (8*R*,9*S*,13*S*,14*S*,17*S*)-3,17-dimethoxy-13-methyl-7,8,9,11,12,13,14,15,16,17-decahydro-6*H*-cyclopenta­[*a*]­phenanthrene (**4f**) (0.800 g, 97%) as an off-white solid. The data were consistent with the literature.^25c^ mp 157–160 °C; [α]_D_^18^ +86.0 (*c* 0.1, CHCl_3_); ^1^H NMR (400 MHz, CDCl_3_): δ 7.21 (d, *J* = 8.6 Hz, 1H), 6.71 (dd, *J* = 8.6, 2.8 Hz, 1H), 6.63 (d, *J* = 2.8 Hz, 1H), 3.78 (s, 3H), 3.38 (s, 3H), 3.32 (t, *J* = 8.3 Hz, 1H), 2.90–2.80 (m, 2H), 2.32–2.26 (m, 1H), 2.12–2.02 (m, 1H), 2.14–1.99 (m, 2H), 1.99–1.85 (m, 1H), 1.73–1.65 (m, 1H), 1.57–1.17 (m, 7H), 0.79 (s, 3H); ^13^C­{^1^H} NMR (101 MHz, CDCl_3_): δ 157.4, 138.0, 132.7, 126.3, 113.8, 111.5, 90.8, 57.9, 55.2, 50.3, 43.9, 43.2, 38.6, 38.1, 29.8, 27.8, 27.2, 26.5, 23.1, 11.6; MS (APCI) *m*/*z* 301 [(M + H)^+^, 100].

### (8*R*,9*S*,13*S*,14*S*,17*S*)-2-Iodo-3,17-dimethoxy-13-methyl-7,8,9,11,12,13,14,15,16,17-decahydro-6*H*-cyclopenta­[*a*]­phenanthrene (**5f**)^52^

The reaction was performed as described in the general procedure using (8*R*,9*S*,13*S*,14*S*,17*S*)-3,17-dimethoxy-13-methyl-7,8,9,11,12,13,14,15,16,17-decahydro-6H-cyclopenta­[*a*]­phenanthrene (150 mg, 0.500 mmol, 1 equiv), *N*-iodosaccharin (**2**) (201 mg, 0.650 mmol, 1.3 equiv), iron trichloride (4.10 mg, 0.0250 mmol, 0.05 equiv), and acetonitrile (1.0 mL) for 0.5 h. (8*R*,9*S*,13*S*,14*S*,17*S*)-2-Iodo-3,17-dimethoxy-13-methyl-7,8,9,11,12,13,14,15,16,17-decahydro-6*H*-cyclopenta­[*a*]­phenanthrene (203 mg, 95%) was obtained as a white solid after purification by column chromatography with 5% ethyl acetate in hexane. The data were consistent with the literature.^52^ mp 127–129 °C; [α]_D_^19^ +85.0 (*c* 0.1, CHCl_3_); ^1^H NMR (400 MHz, CDCl_3_): δ 7.64 (s, 1H), 6.54 (s, 1H), 3.83 (s, 3H), 3.38 (s, 3H), 3.31 (dd, *J* = 8.8, 7.8 Hz, 1H), 2.85–2.78 (m, 2H), 2.30–2.11 (m, 2H), 2.10–1.98 (m, 2H), 1.93–1.83 (m, 1H), 1.68 (dddd, *J* = 12.3, 9.7, 6.9, 3.2 Hz, 1H), 1.58–1.44 (m, 2H), 1.43–1.25 (m, 4H), 1.19 (ddd, *J* = 12.3, 10.6, 7.1 Hz, 1H), 0.78 (s, 3H); ^13^C­{^1^H} NMR (101 MHz, CDCl_3_): δ 155.9, 138.4, 136.4, 135.1, 111.4, 90.7, 82.6, 57.9, 56.3, 50.2, 43.6, 43.2, 38.4, 37.9, 29.7, 27.8, 27.0, 26.4, 23.0, 11.5; MS (APCI) *m*/*z* 426 (M^+^, 100).

### General Procedure for One-Pot Iodination–Arylation Reaction

To an oven-dried microwave tube fitted with a magnetic stirrer was added *N*-iodosaccharin (**2**) (201 mg, 0.650 mmol, 1.30 equiv) and iron trichloride (4.10 mg, 0.0250 mmol, 5 mol %) under an atmosphere of argon. Degassed anhydrous acetonitrile (1 mL) was then added followed by substrate (0.5 mmol, 1 equiv), and the mixture was stirred at room temperature for 0.5 h. To the reaction mixture was added water (2 mL), arylboronic acid (0.750 mmol, 1.50 equiv), and potassium phosphate tribasic (0.700 mmol, 1.4 equiv). The reaction mixture was degassed under argon for 0.2 h. To this solution was added XPhos Pd G2 (19.7 mg, 0.0250 mmol, 5 mol %), and the reaction mixture was stirred at 80 °C. Reactions were monitored by TLC or NMR spectroscopy. After completion of the reaction, the mixture was allowed to cool to room temperature, filtered through a short Celite pad, and washed with ethyl acetate (20 mL). The filtrate was diluted with water (20 mL) and extracted with ethyl acetate (3 × 10 mL). The combined organic layers were washed with brine (50 mL), dried (MgSO_4_), filtered, concentrated in vacuo, and purified by flash column chromatography.

### 4-Methoxybiphenyl (**6a**)^53^

The reaction was performed as described in the general procedure using anisole (**1a**) (54.3 μL, 0.500 mmol, 1.00 equiv) and phenyl boronic acid (91.4 mg, 0.750 mmol, 1.50 equiv). The Suzuki–Miyaura reaction required a reaction time of 4.5 h. 4-Methoxybiphenyl (**6a**) (70.0 mg, 76%) was obtained as a white solid after purification by column chromatography with 5% ethyl acetate in hexane. mp 91–93 °C (lit.^53^ 90–91 °C); ^1^H NMR (400 MHz, CDCl_3_): δ 7.58–7.51 (m, 4H), 7.42 (t, *J* = 7.7 Hz, 2H), 7.31 (t, *J* = 7.7 Hz, 1H), 6.99 (d, *J* = 8.9 Hz, 2H), 3.86 (s, 3H); ^13^C­{^1^H} NMR (101 MHz, CDCl_3_): δ 159.2, 140.9, 133.8, 128.7, 128.2, 126.8, 126.7, 114.2, 55.4; MS (APCI) *m*/*z* 184 (M^+^, 100).

### 2-(4-Methoxyphenyl)­naphthalene (**6b**)^54^

The reaction was performed as described in the general procedure using anisole (54.3 μL, 0.500 mmol, 1.00 equiv) and 2-naphthyl boronic acid (129 mg, 0.750 mmol, 1.50 equiv). The Suzuki–Miyaura reaction required a reaction time of 4 h. 2-(4-Methoxyphenyl)­naphthalene (**6b**) (80.0 mg, 68%) was obtained as a white solid after purification by column chromatography with 25% dichloromethane in hexane. The data were consistent with the literature.^54^ mp 128–130 °C; ^1^H NMR (400 MHz, CDCl_3_): δ 8.00 (s, 1H), 7.94–7.84 (m, 3H), 7.73 (dd, *J* = 8.5, 1.8 Hz, 1H), 7.68 (d, *J* = 8.9 Hz, 2H), 7.55–7.42 (m, 2H), 7.04 (d, *J* = 8.9 Hz, 2H), 3.88 (s, 3H); ^13^C­{^1^H} NMR (101 MHz, CDCl_3_): δ 159.3, 138.2, 133.8, 133.7, 132.3, 128.5, 128.4, 128.1, 127.7, 126.3, 125.7, 125.5, 125.1, 114.4, 55.4; MS (APCI) *m*/*z* 235 [(M + H)^+^, 100].

### 4′-Methoxybiphenyl-4-carbonitrile (**6c**)^55^

The reaction was performed as described in the general procedure using anisole (54.3 μL, 0.500 mmol, 1.00 equiv) and 4-cyanophenylboronic acid (110 mg, 0.750 mmol, 1.50 equiv). The Suzuki–Miyaura reaction required a reaction time of 4 h. 4′-Methoxybiphenyl-4-carbonitrile (**6c**) (58.0 mg, 56%) was obtained as a white solid after purification by column chromatography with 5% ethyl acetate in hexane. The data were consistent with the literature.^55^ mp 101–103 °C; ^1^H NMR (400 MHz, CDCl_3_): δ 7.72–7.68 (m, 2H), 7.65–7.63 (m, 2H), 7.57–7.51 (m, 2H), 7.04–6.98 (m, 2H), 3.87 (s, 3H); ^13^C­{^1^H} NMR (101 MHz, CDCl_3_): δ 160.2, 145.2, 132.6, 131.5, 128.4, 127.1, 119.1, 114.6, 110.1, 55.4; MS (APCI) *m*/*z* 208 [(M – H)^−^, 100].

### Methyl (2S)-2′-(6′-Methoxy-5′-phenylnaphthalen-2′-yl)-2-methylethanoate (**6d**)

The reaction was performed as described in the general procedure using anisole (2*S*)-2′-(6′-methoxynaphthalen-2′-yl)-2-methylethanoate (122 mg, 0.500 mmol, 1.00 equiv) and phenylboronic acid (91.4 mg, 0.750 mmol, 1.50 equiv). The Suzuki–Miyaura reaction required a reaction time of 3 h. Methyl (2*S*)-2′-(6′-methoxy-5′-phenylnaphthalen-2′-yl)-2-methylethanoate (**6d**) was obtained as a colorless oil (130 mg, 80%) after purification by column chromatography with 4% ethyl acetate in hexane. [α]_D_^17^ +47.0 (*c* 0.1, CHCl_3_); IR (neat) 2949, 2838, 1732, 1596, 1456, 1254, 1161, 1062 cm^–1^; ^1^H NMR (400 MHz, CDCl_3_): δ 7.86 (d, *J* = 8.8 Hz, 1H), 7.73 (d, *J* = 1.9 Hz, 1H), 7.55–7.34 (m, 7H), 7.32 (dd, *J* = 8.8, 1.9 Hz, 1H), 3.87 (q, *J* = 7.2 Hz, 1H), 3.84 (s, 3H), 3.67 (s, 3H), 1.58 (d, *J* = 7.2 Hz, 3H); ^13^C­{^1^H} NMR (101 MHz, CDCl_3_): δ 175.1, 153.8, 136.3, 135.6, 132.8, 130.9, 129.03, 128.98, 128.2, 127.1, 126.2, 126.0, 125.9, 125.4, 114.2, 56.8, 52.1, 45.3, 18.6; HRMS (ESI) *m*/*z*: [M + H]^+^ calcd for C_21_H_20_O_3_H 321.1485; found, 321.1488.

## Supplementary Material



## Data Availability

The data underlying this study are available in the published article and its online Supporting Information.

## Notes

^1^H and ^13^C NMR spectra of all compounds (PDF)
