# Supplementary material for: Regioselective Iodination of Arenes Using Iron- or Silver-Catalyzed Activation of N‑Iodosaccharin
Source: J Org Chem. 2026 Jan 27;91(5):2257–67. doi: 10.1021/acs.joc.5c03183 (PMC12887992; doi:10.1021/acs.joc.5c03183)

Supporting Information for:

Regioselective Iodination of Arenes using Iron- or Silver-Catalyzed Activation of *N*-Iodosaccharin

*Pankaj K. Majhi, Catherine M. Fleming and Andrew Sutherland**

School of Chemistry, The Joseph Black Building, University of Glasgow, Glasgow G12 8QQ, United Kingdom.

Table of Contents

1. Racemic and Chiral HPLC Traces for Compound **6d** S2–S3

2. ^{1}H and ^{13}C NMR Spectra for all Compounds S4–S81

1. Racemic and Chiral HPLC Traces for Compound 6d

The chiral HPLC method was calibrated with the corresponding racemic mixture and performed using an AD-H column, 5% isopropanol in hexane and a 1 mL min^{-1} flow rate.

Racemate **6d**:

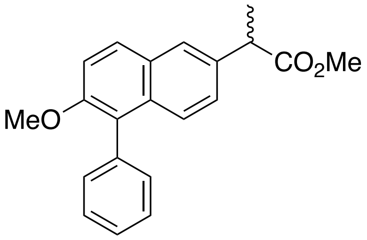

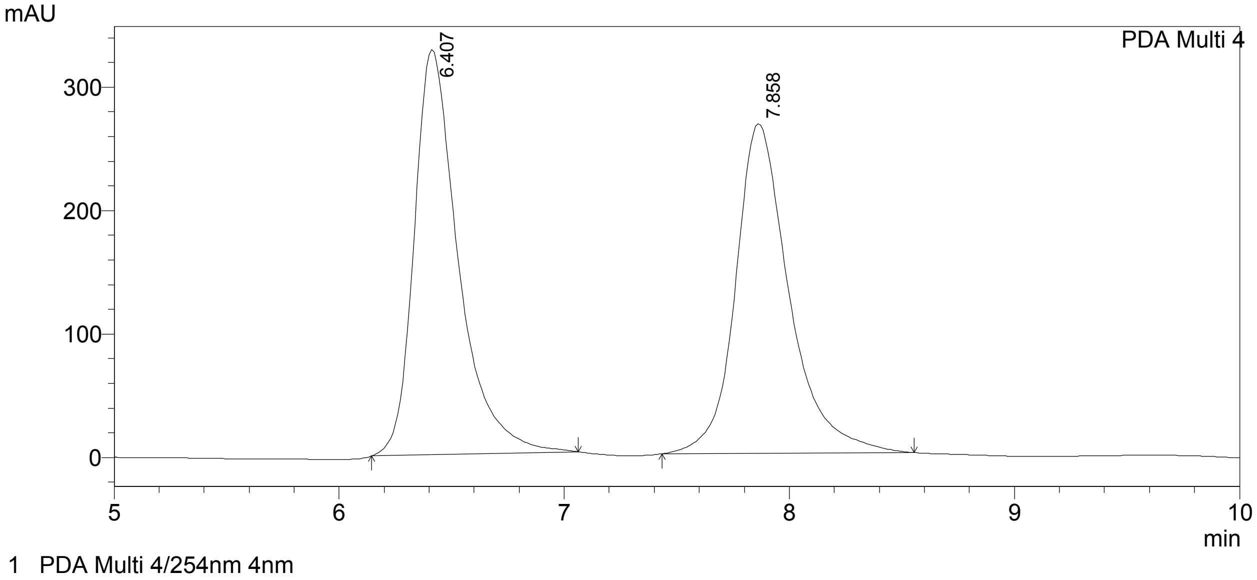

PDA Ch4 254nm 4nm

Peak#	Ret. Time	Area	Height	Area %	Height %
1	6.407	4483289	327933	50.283	55.102
2	7.858	4432753	267201	49.717	44.898
Total		8916043	595134	100.000	100.000

(*S*)-Enantiomer **6d**:

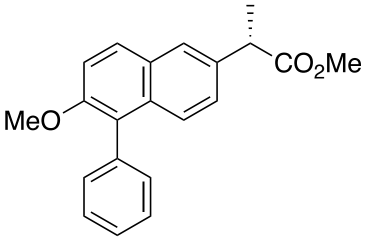

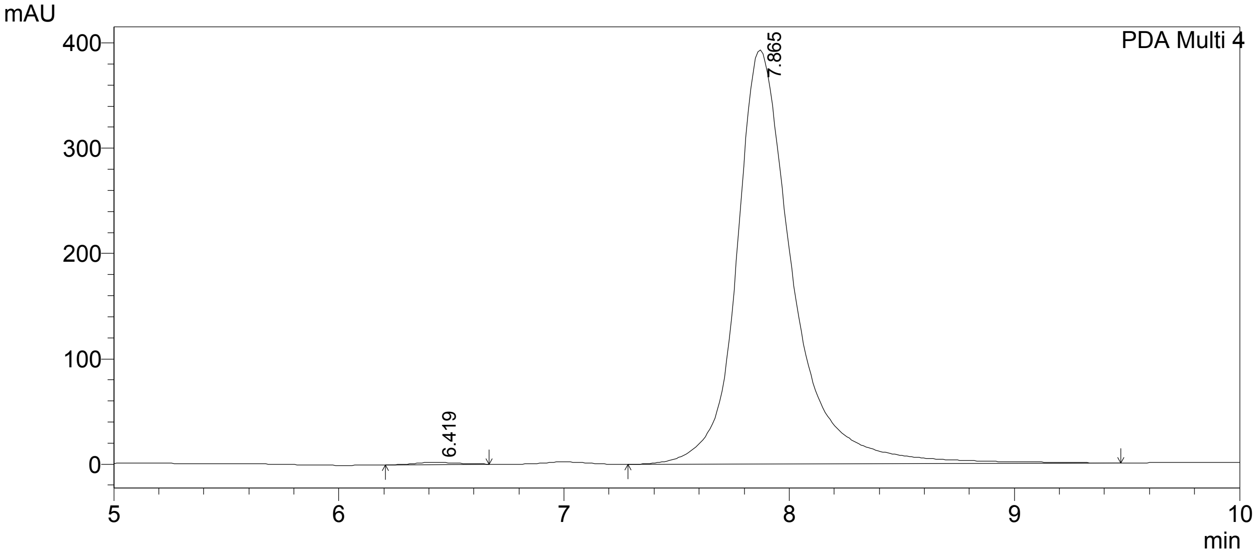

1 PDA Multi 4/254nm 4nm

PDA Ch4 254nm 4nm

Peak#	Ret. Time	Area	Height	Area %	Height %
1	6.419	26121	2109	0.381	0.534
2	7.865	6828799	393044	99.619	99.466
Total		6854921	395154	100.000	100.000

2. ^{1}H and ^{13}C NMR Spectra for all Compounds

^{1}H NMR [400 MHz, $(CD_3)_2CO$]

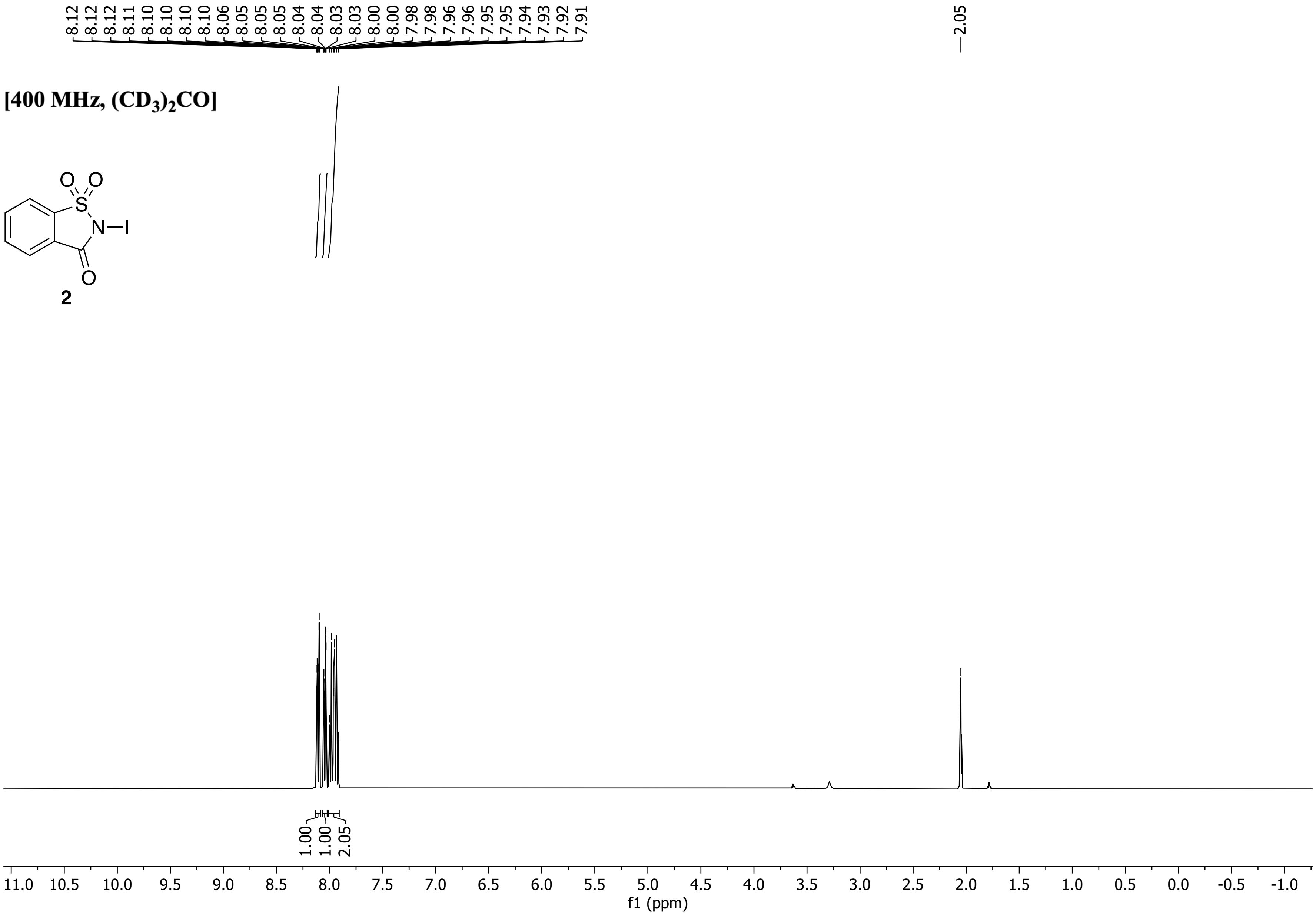

2

$^{13}C\{^1H\}$ NMR [101 MHz, $(CD_3)_2CO$]

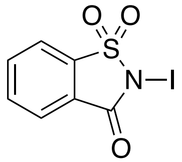

2

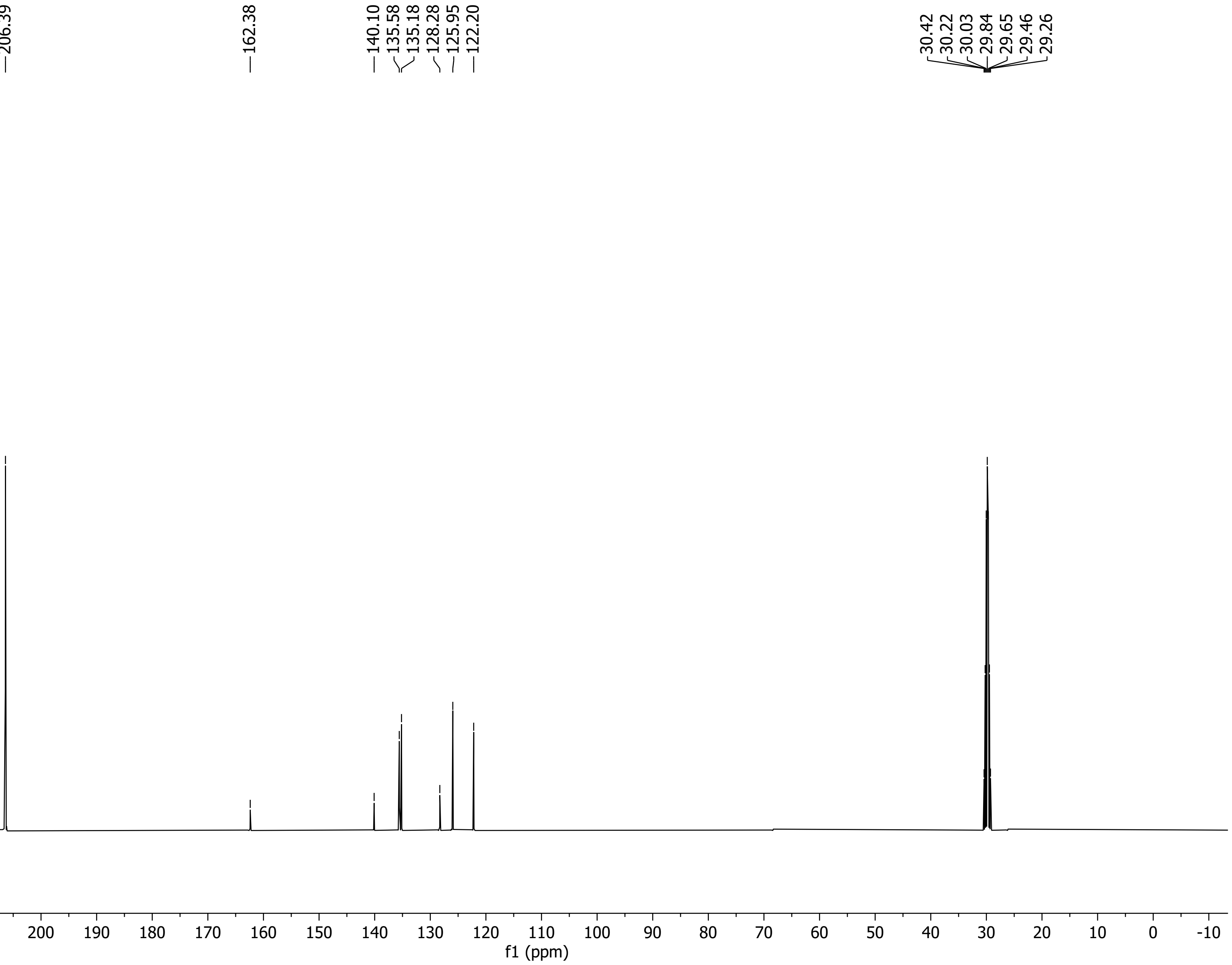

^{1}H NMR (400 MHz, $CDCl_3$)

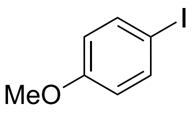

3a

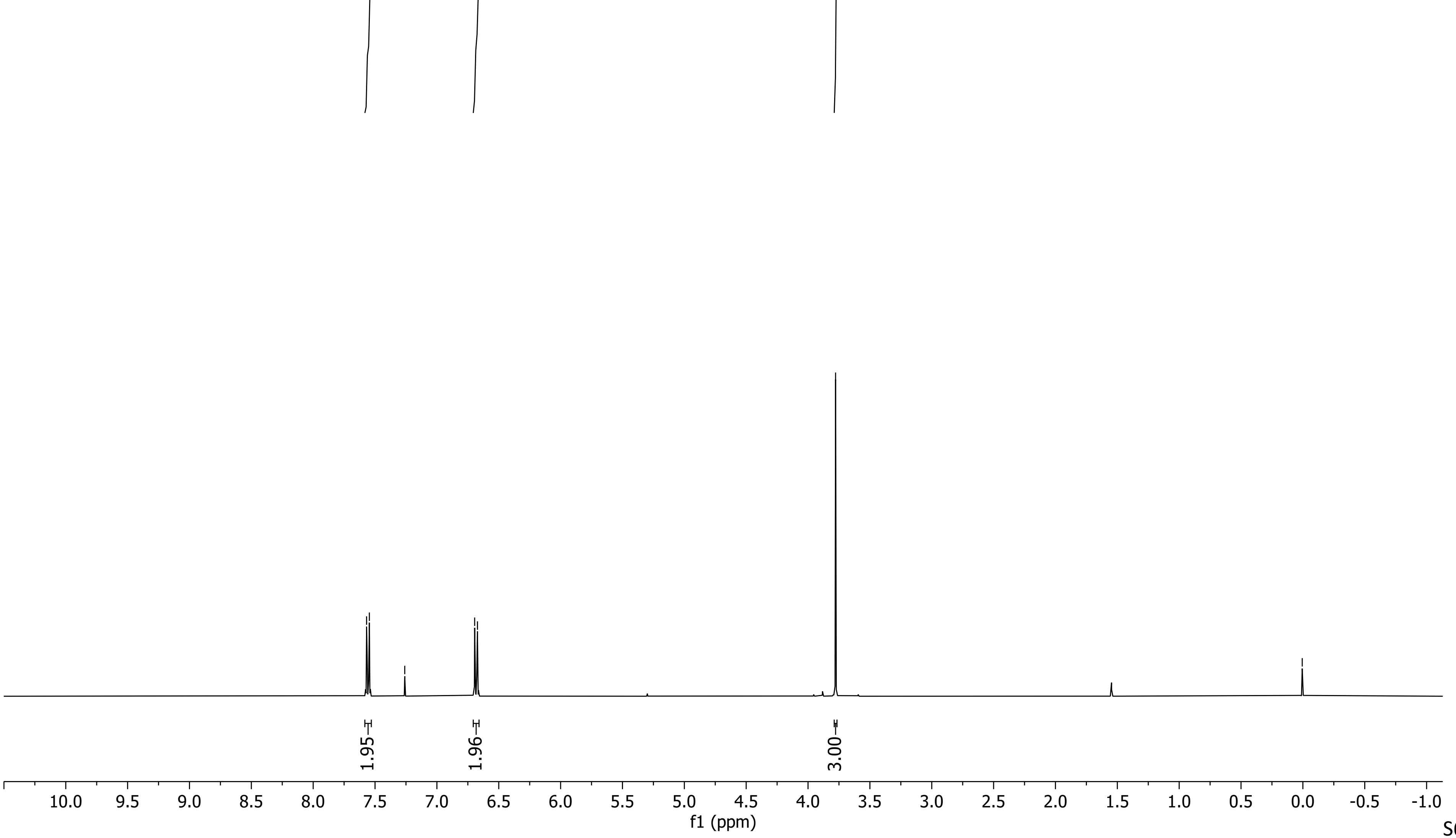

$^{13}C\{^{1}H\}$ NMR (101 MHz, $CDCl_3$)

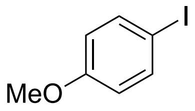

3a

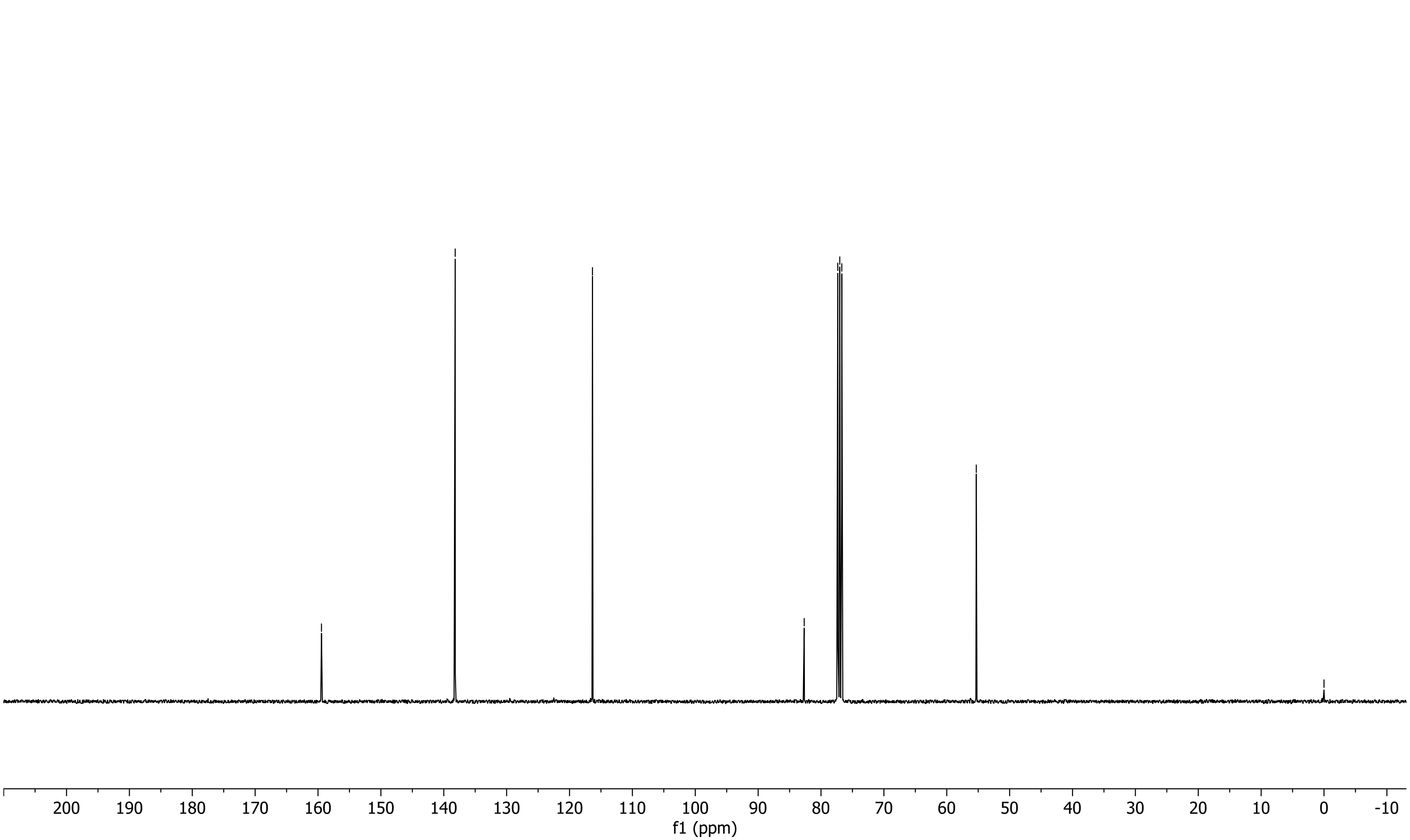

^{1}H NMR (400 MHz, $CDCl_3$)

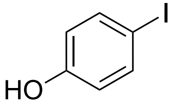

3b

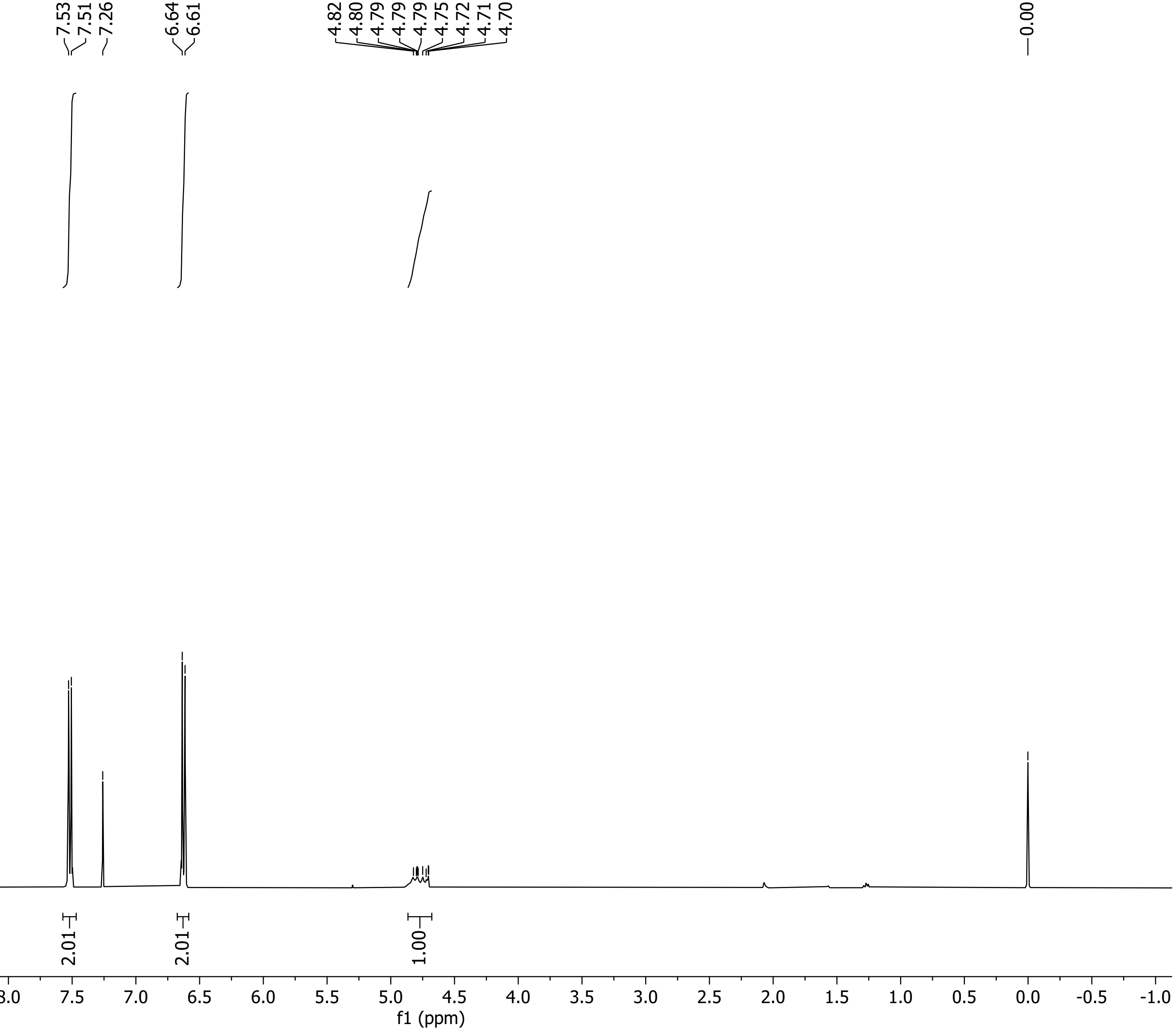

$^{13}C\{^{1}H\}$ NMR (101 MHz, $CDCl_3$)

3b

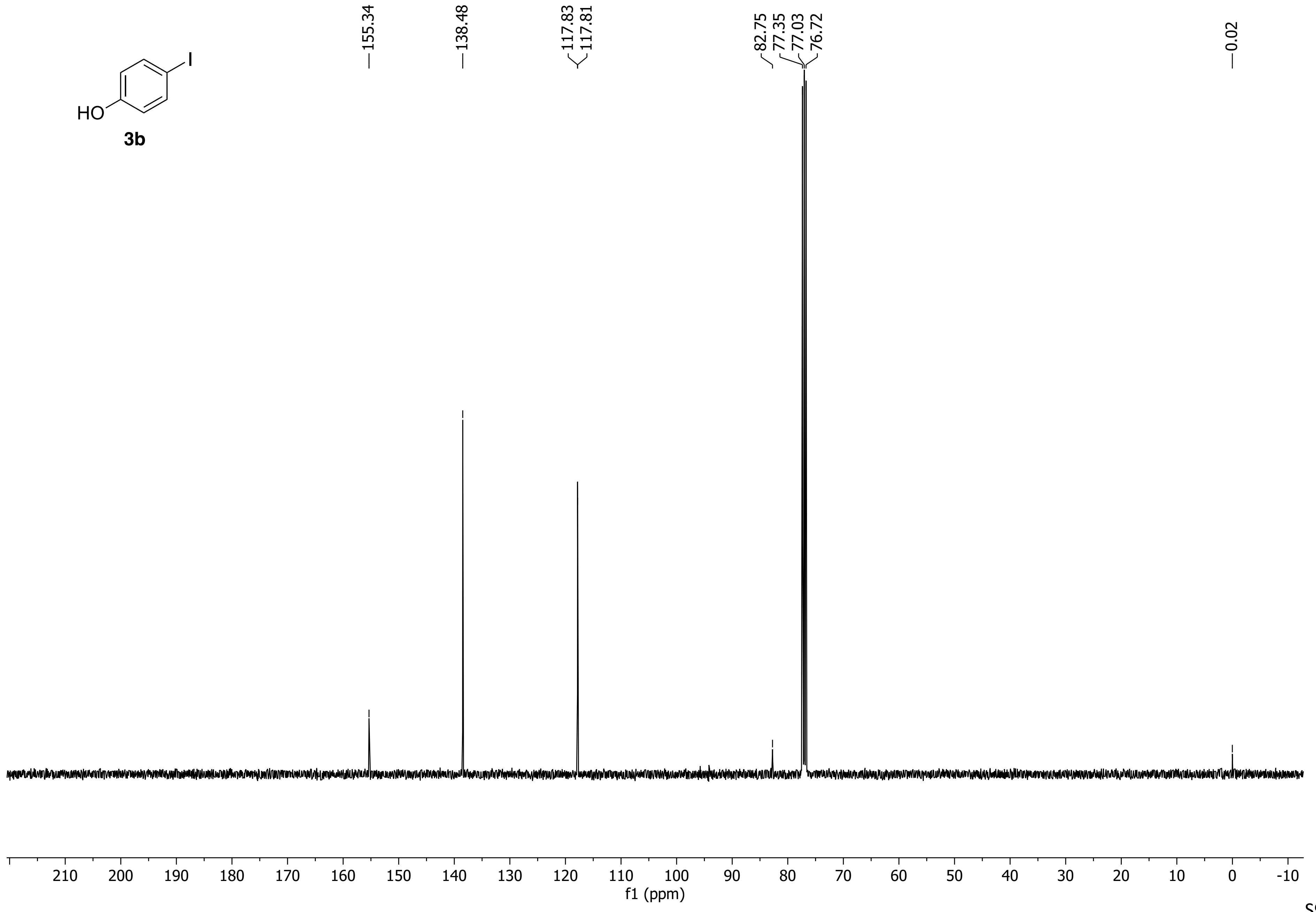

^{1}H NMR (400 MHz, $CDCl_3$)

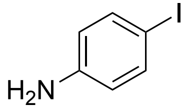

3c

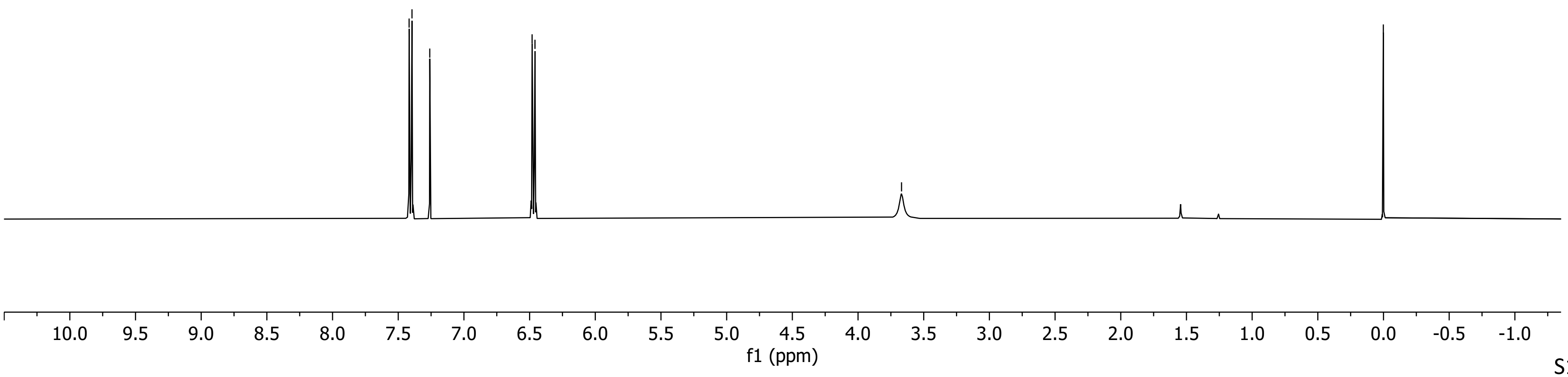

$^{13}C\{^{1}H\}$ NMR (101 MHz, $CDCl_3$)

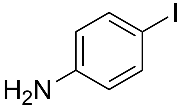

3c

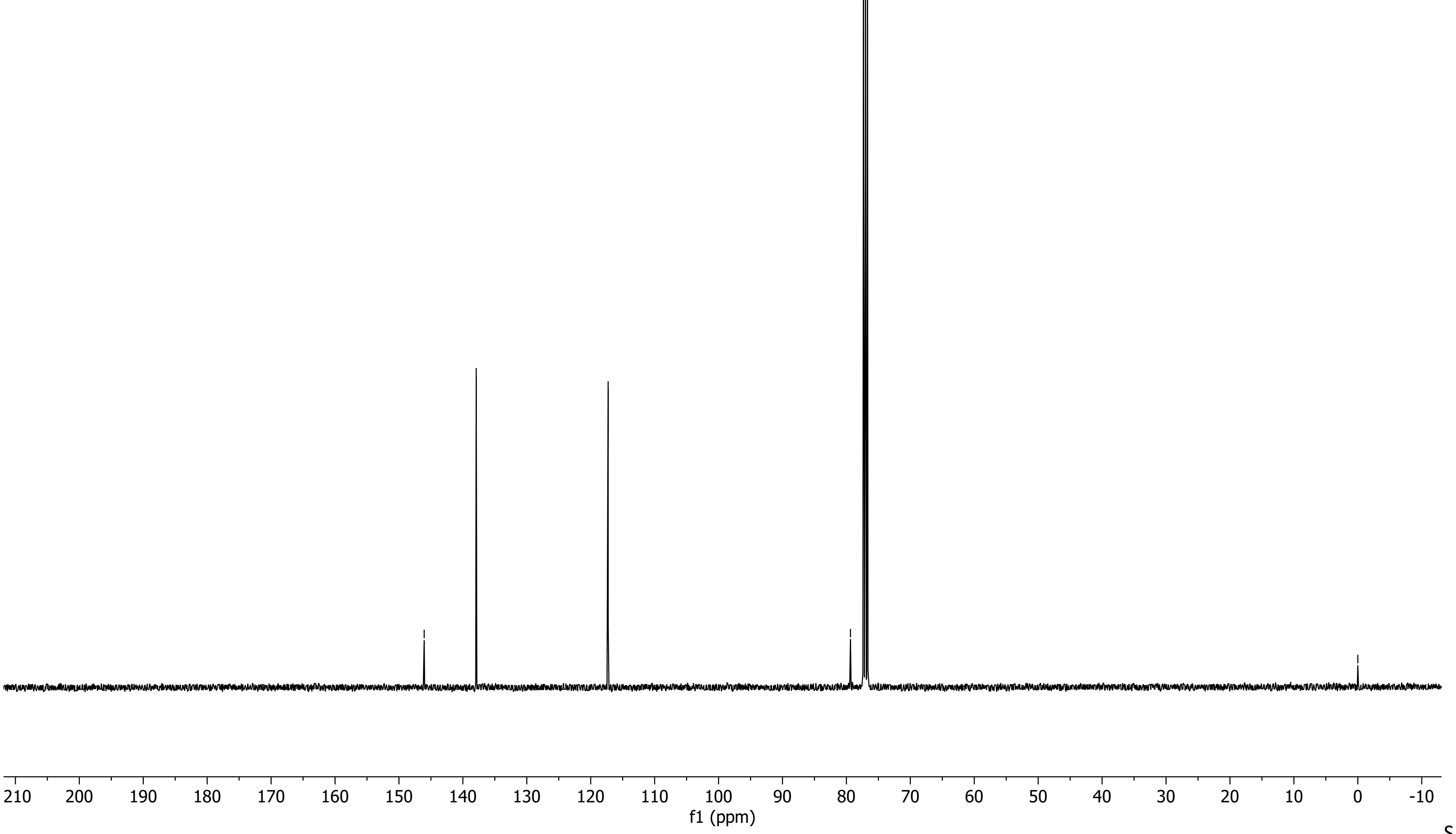

^{1}H NMR (400 MHz, CD_3OD)

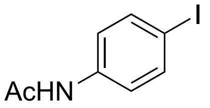

3d

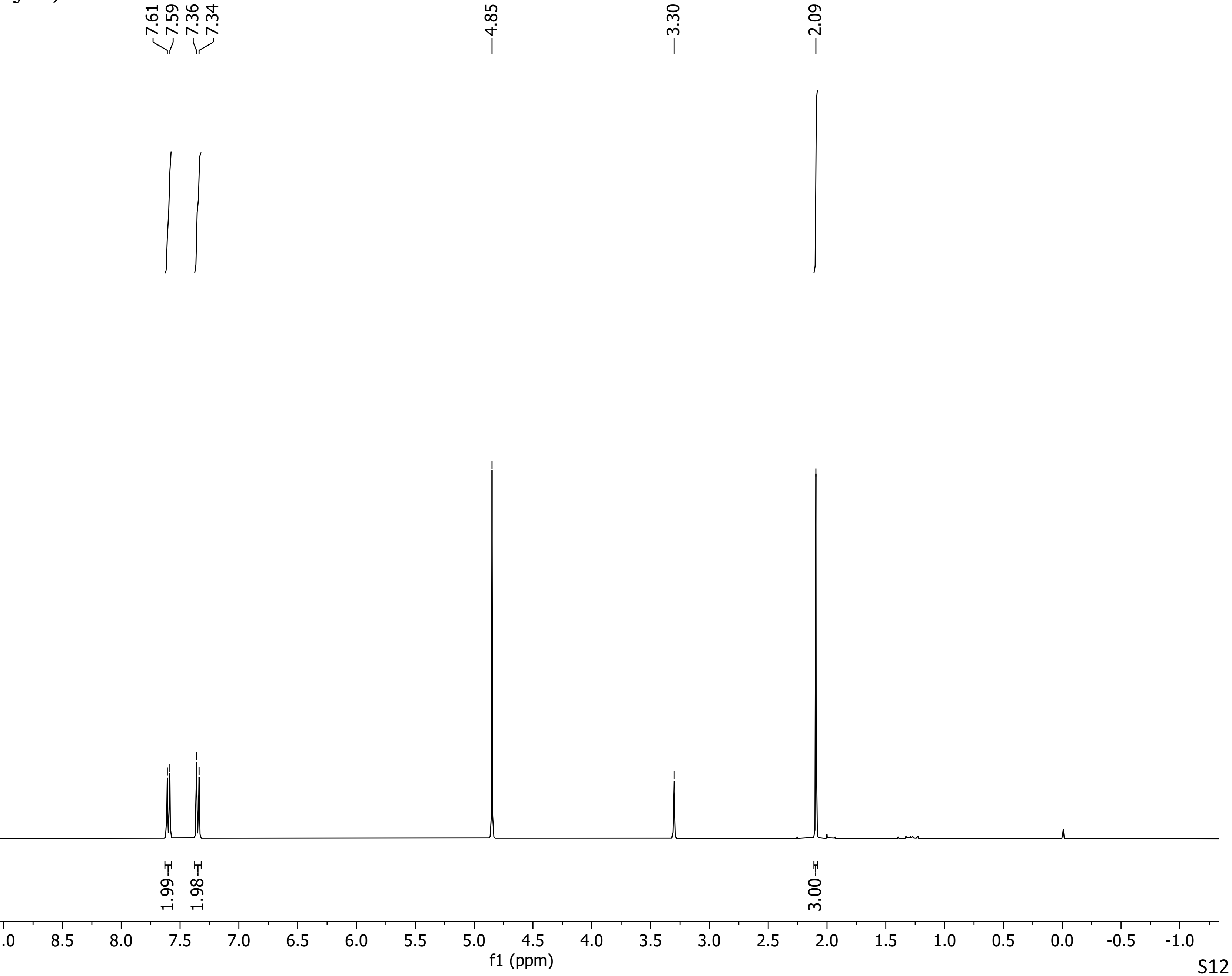

$^{13}C\{^{1}H\}$ NMR (101 MHz, CD_3OD)

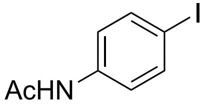

3d

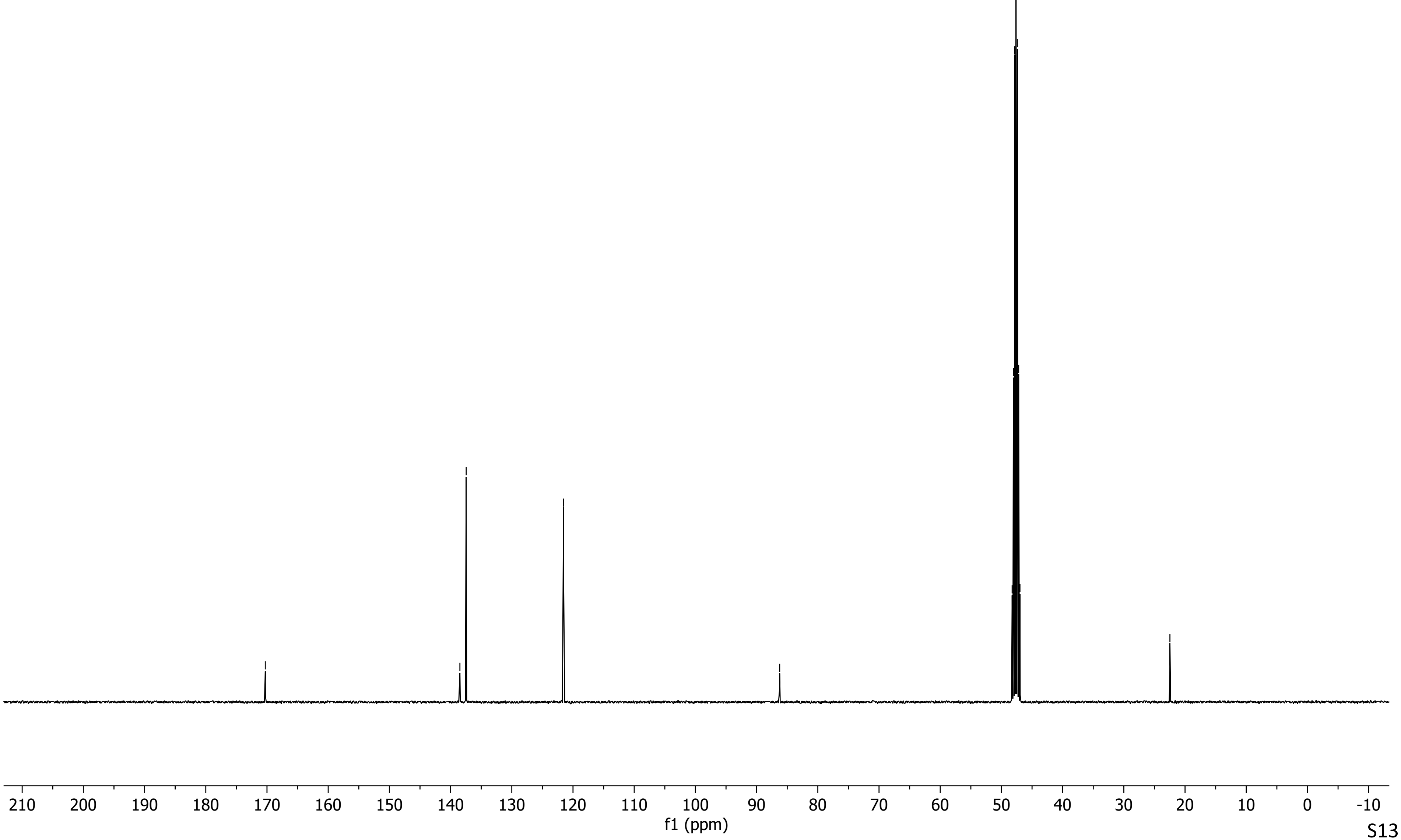

^{1}H NMR (400 MHz, $CDCl_3$)

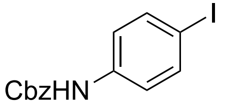

3e

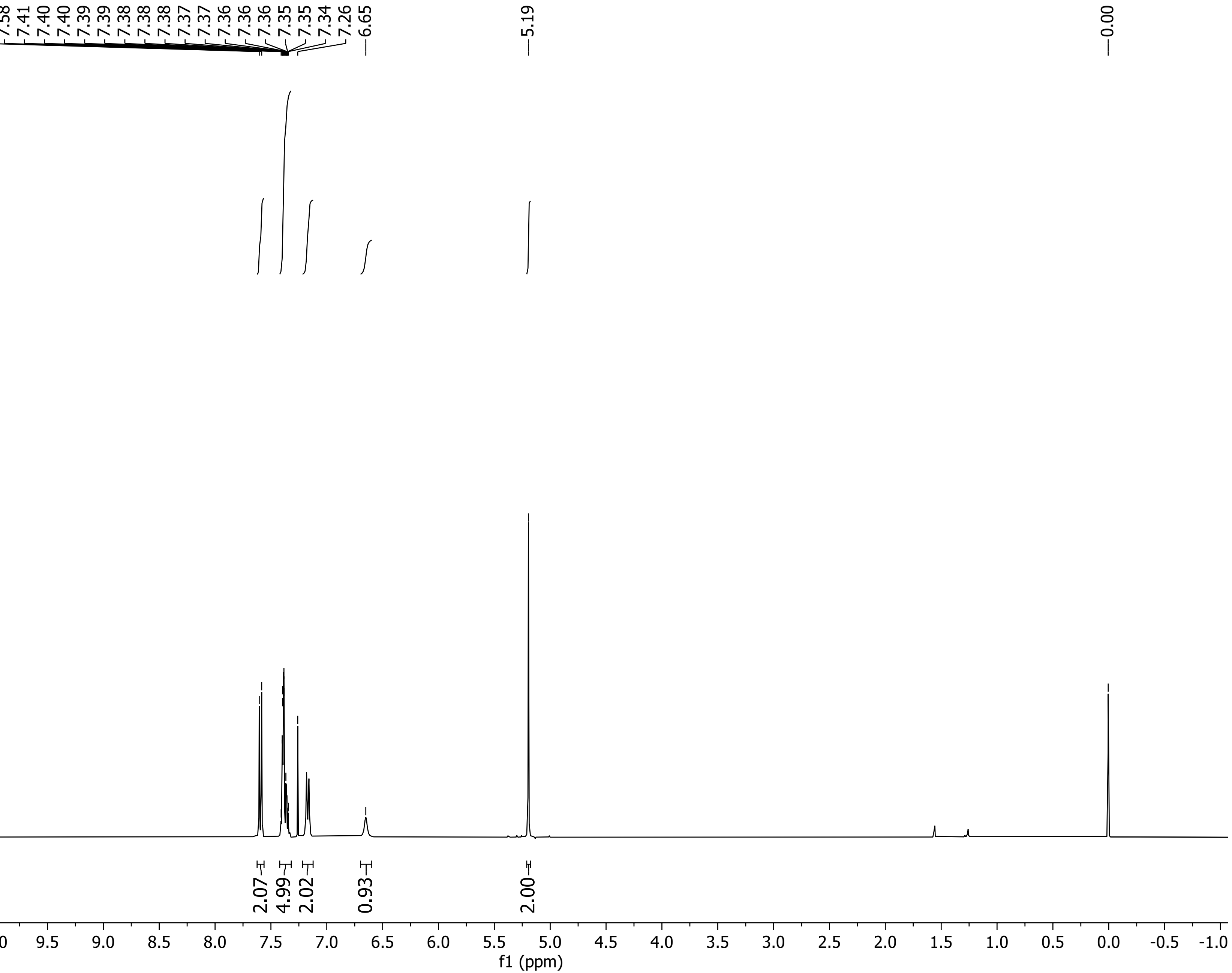

$^{13}C\{^1H\}$ **NMR (101 MHz, $CDCl_3$)**

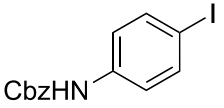

3e

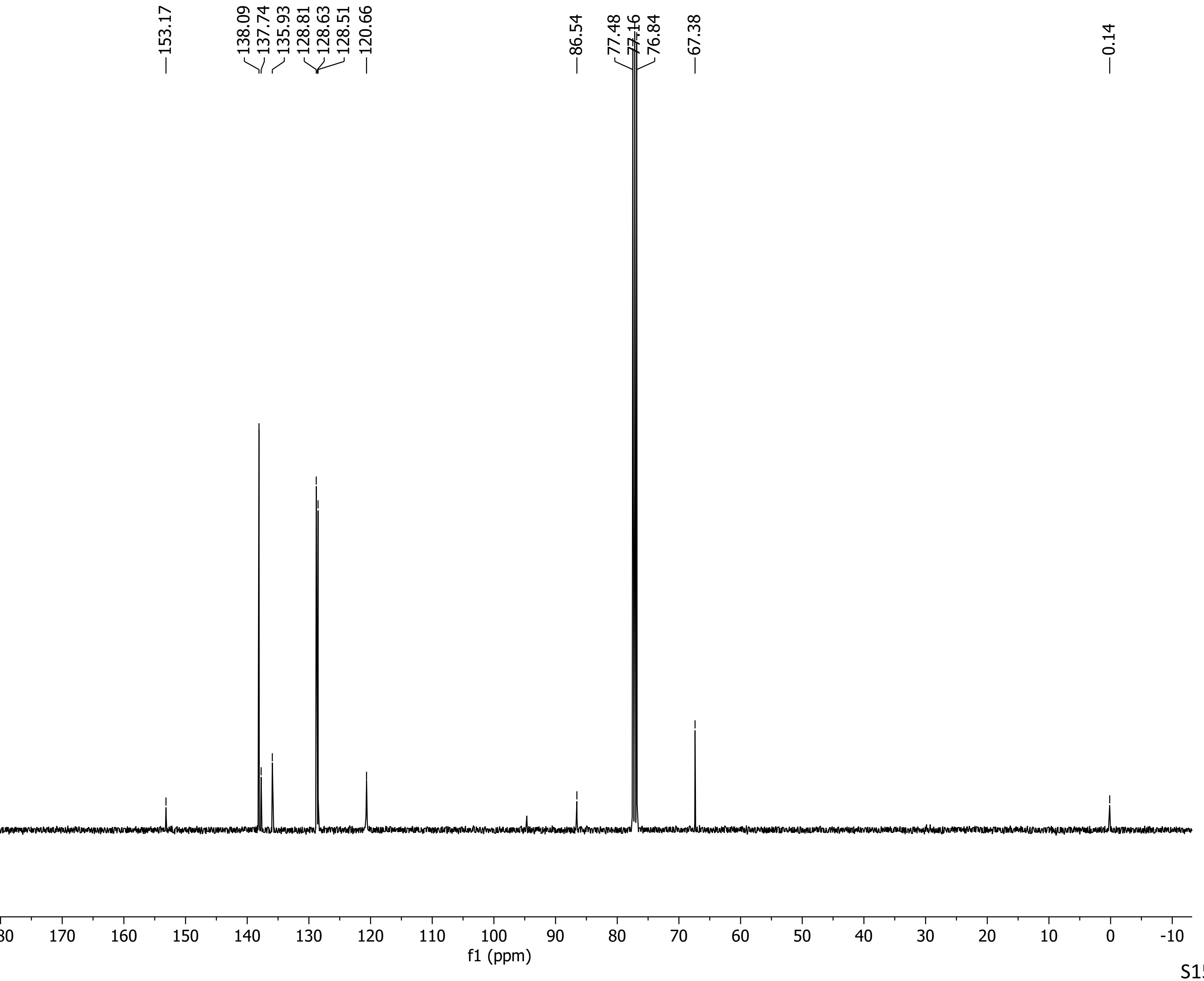

^{1}H NMR (400 MHz, $CDCl_3$)

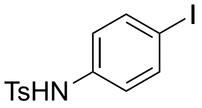

3f

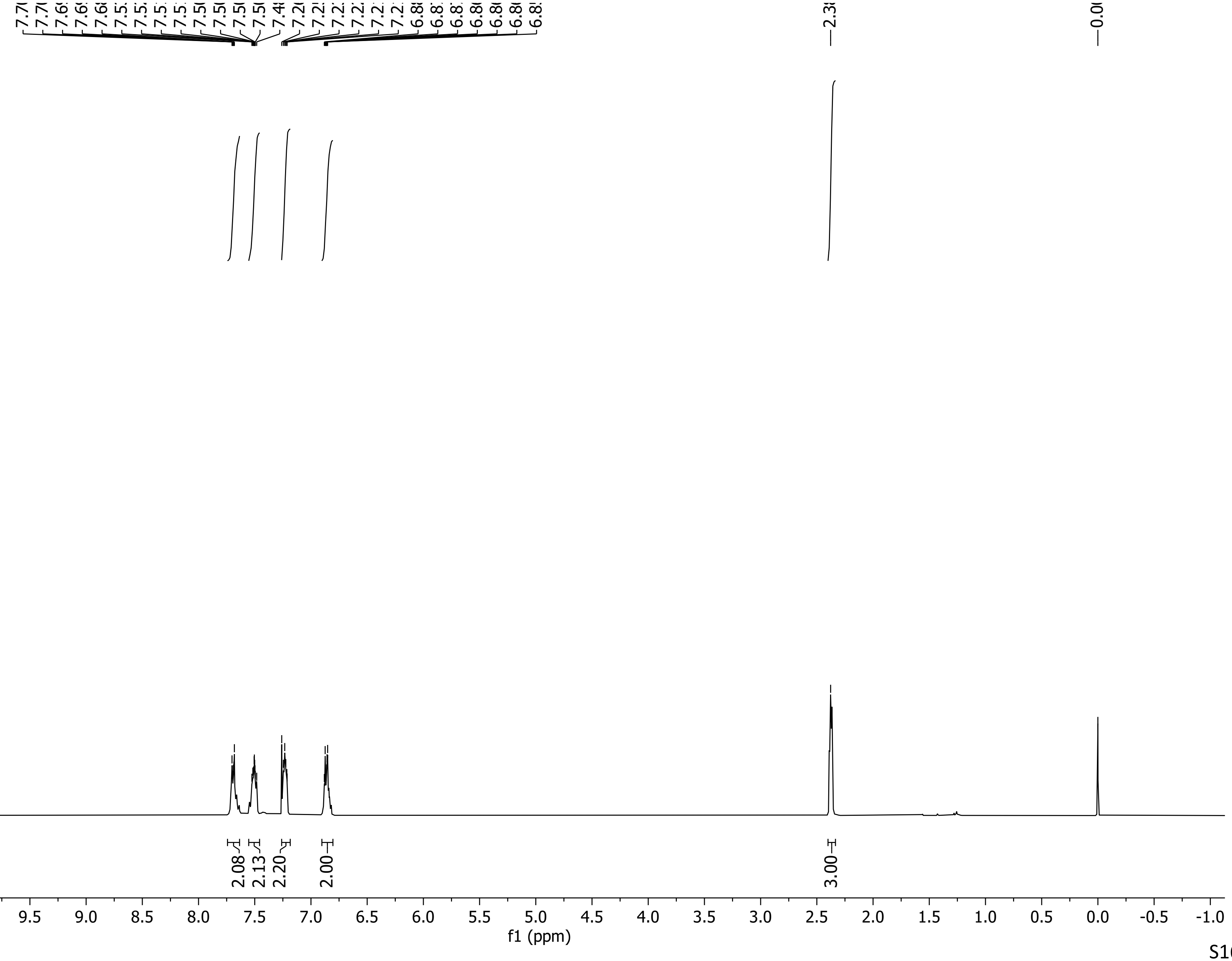

$^{13}C\{^{1}H\}$ NMR (101 MHz, $CDCl_3$)

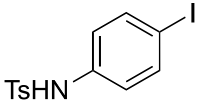

3f

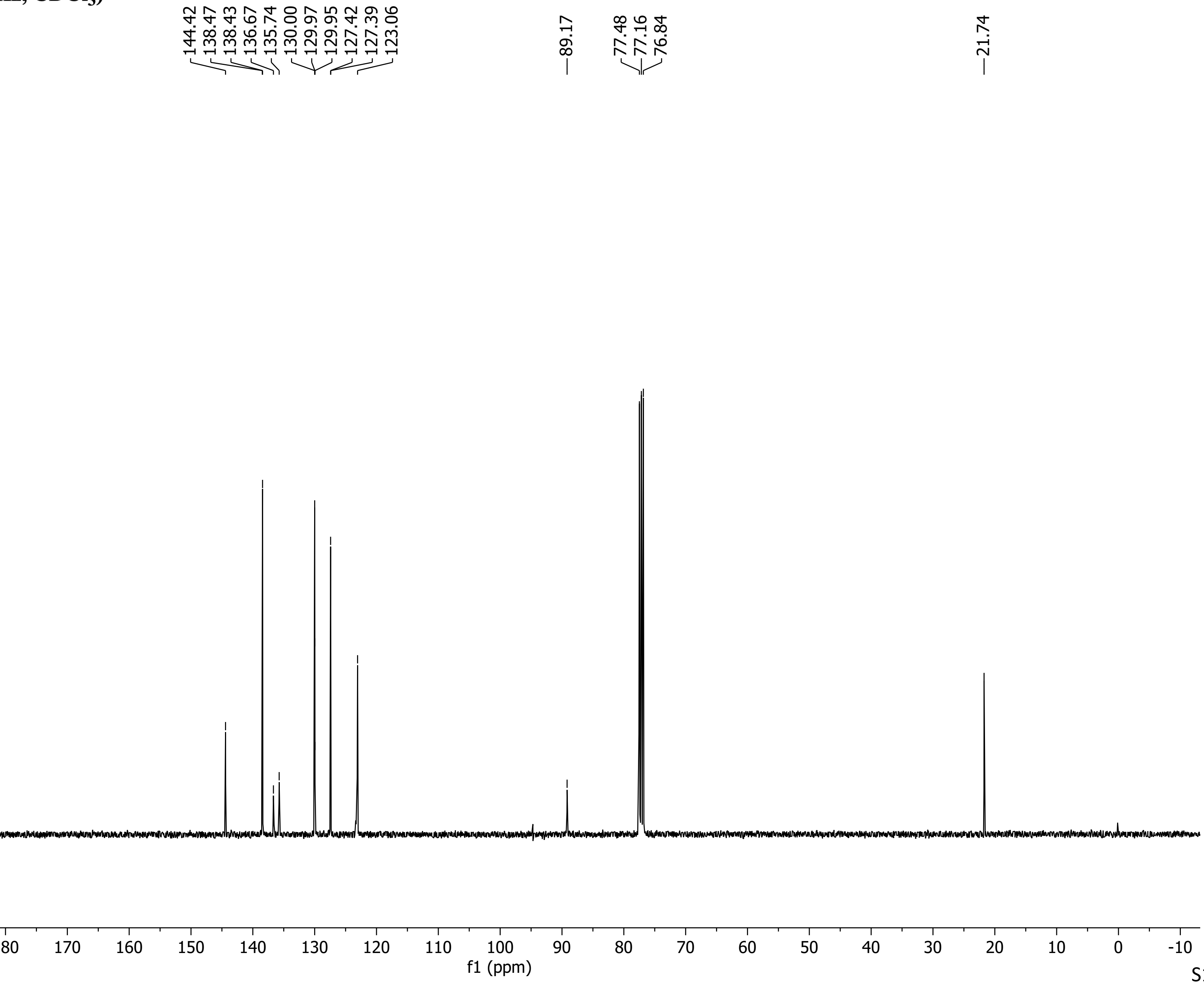

^{1}H NMR (400 MHz, $CDCl_3$)

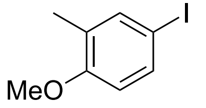

3g

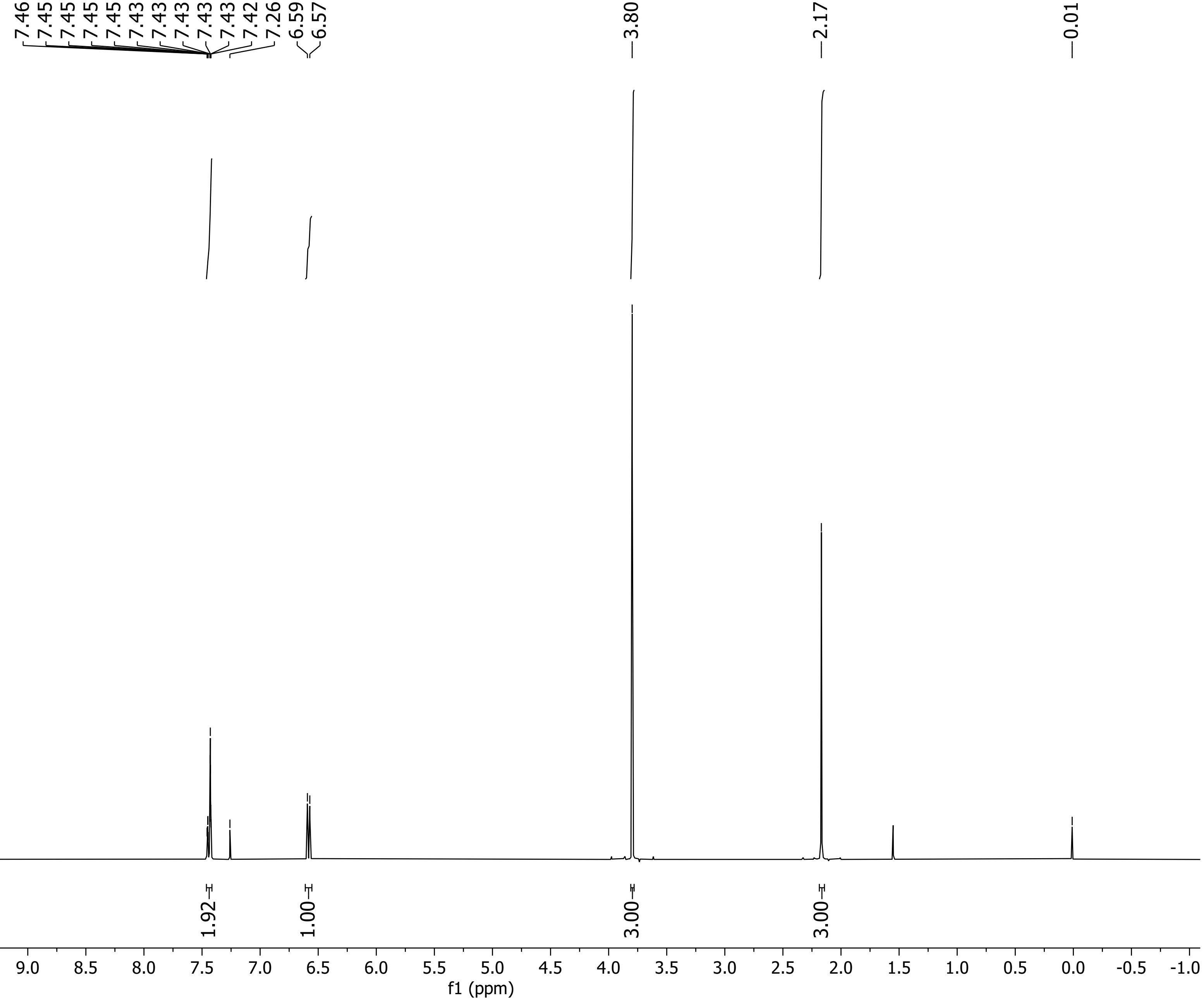

$^{13}C\{^{1}H\}$ NMR (101 MHz, $CDCl_3$)

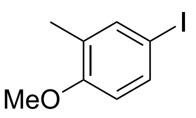

3g

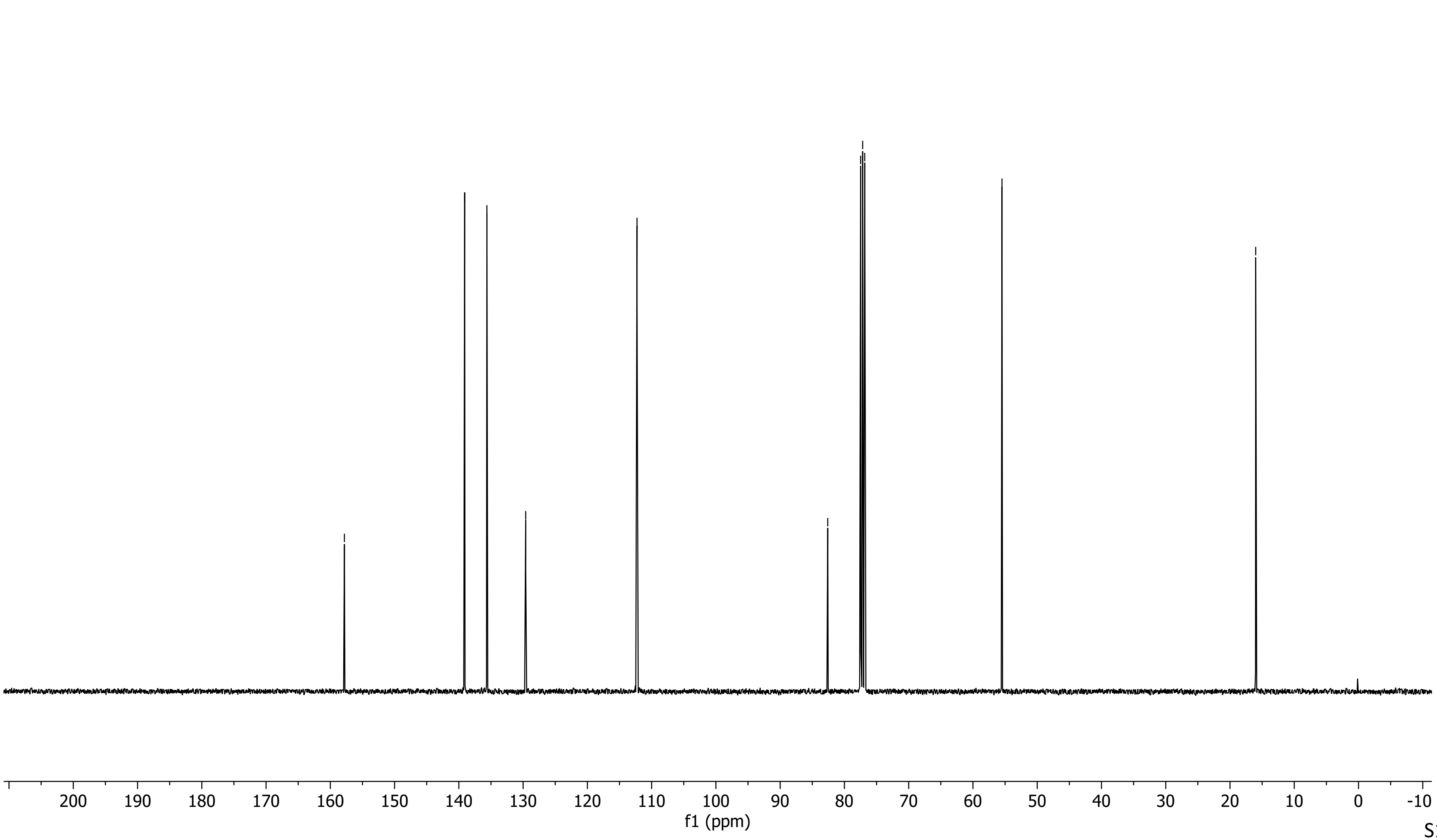

^{1}H NMR (400 MHz, CD_3OD)

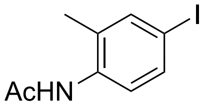

3h

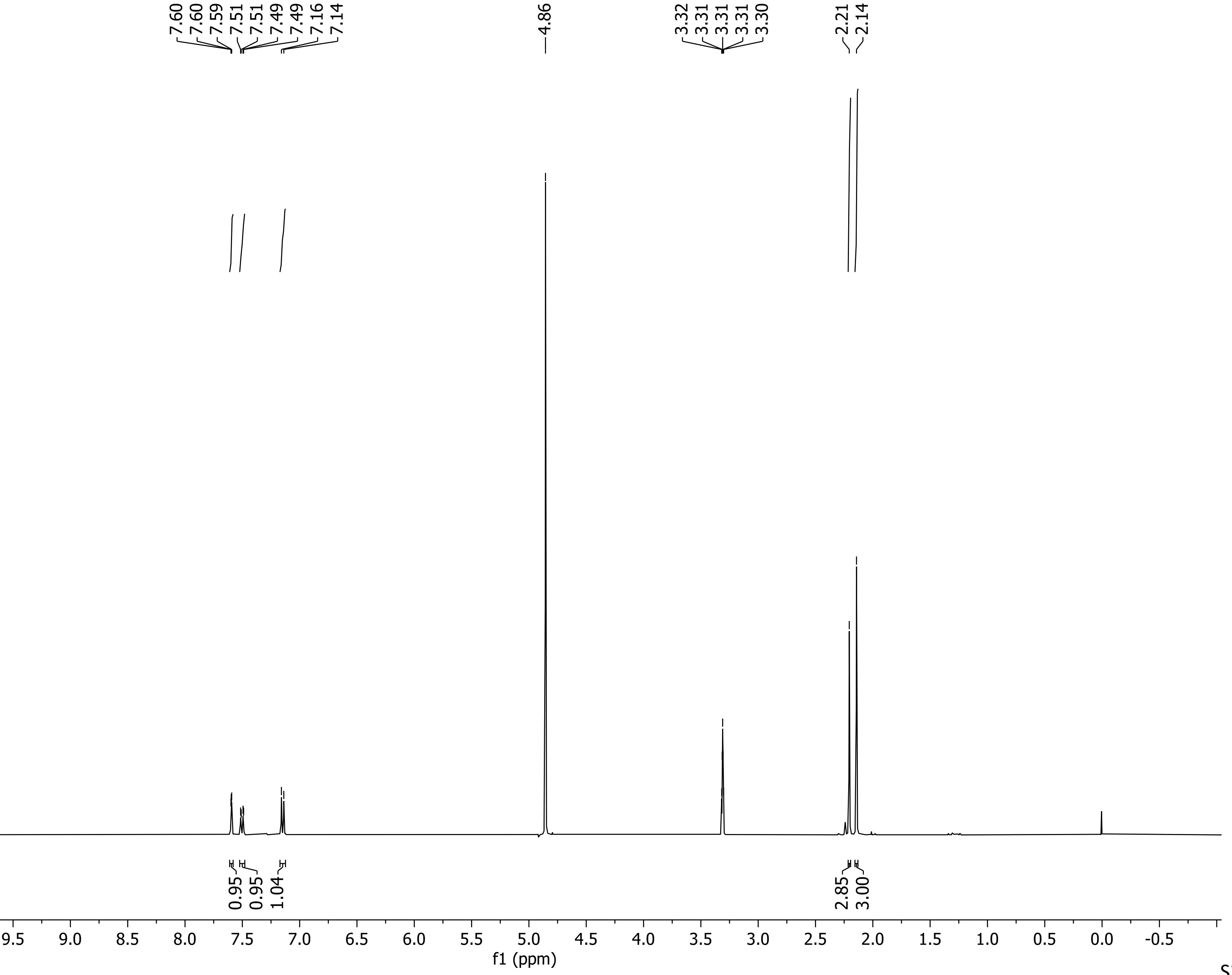

$^{13}C\{^1H\}$ NMR (101 MHz, CD_3OD)

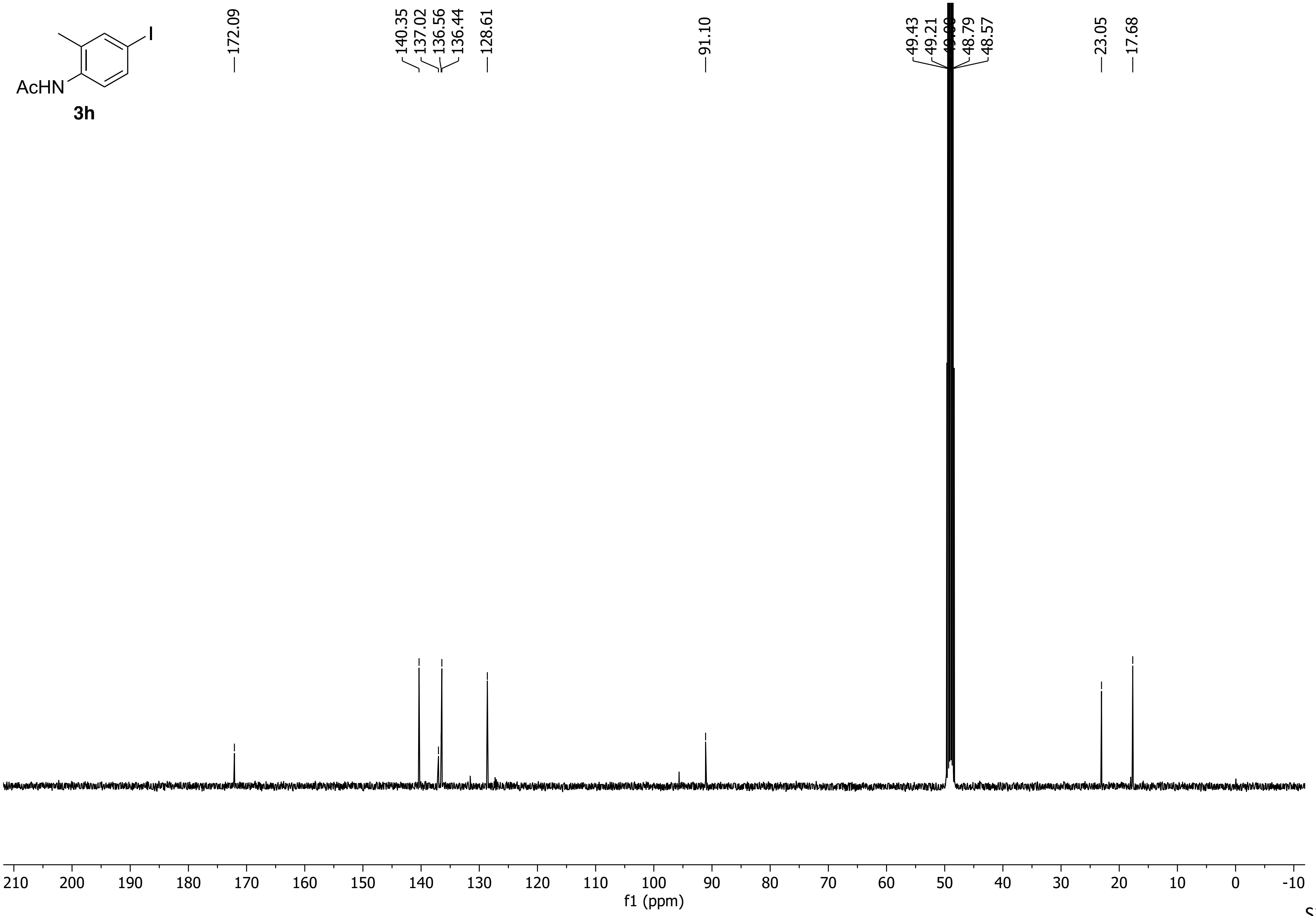

^{1}H NMR (400 MHz, $CDCl_3$)

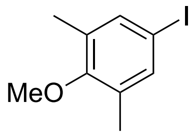

3i

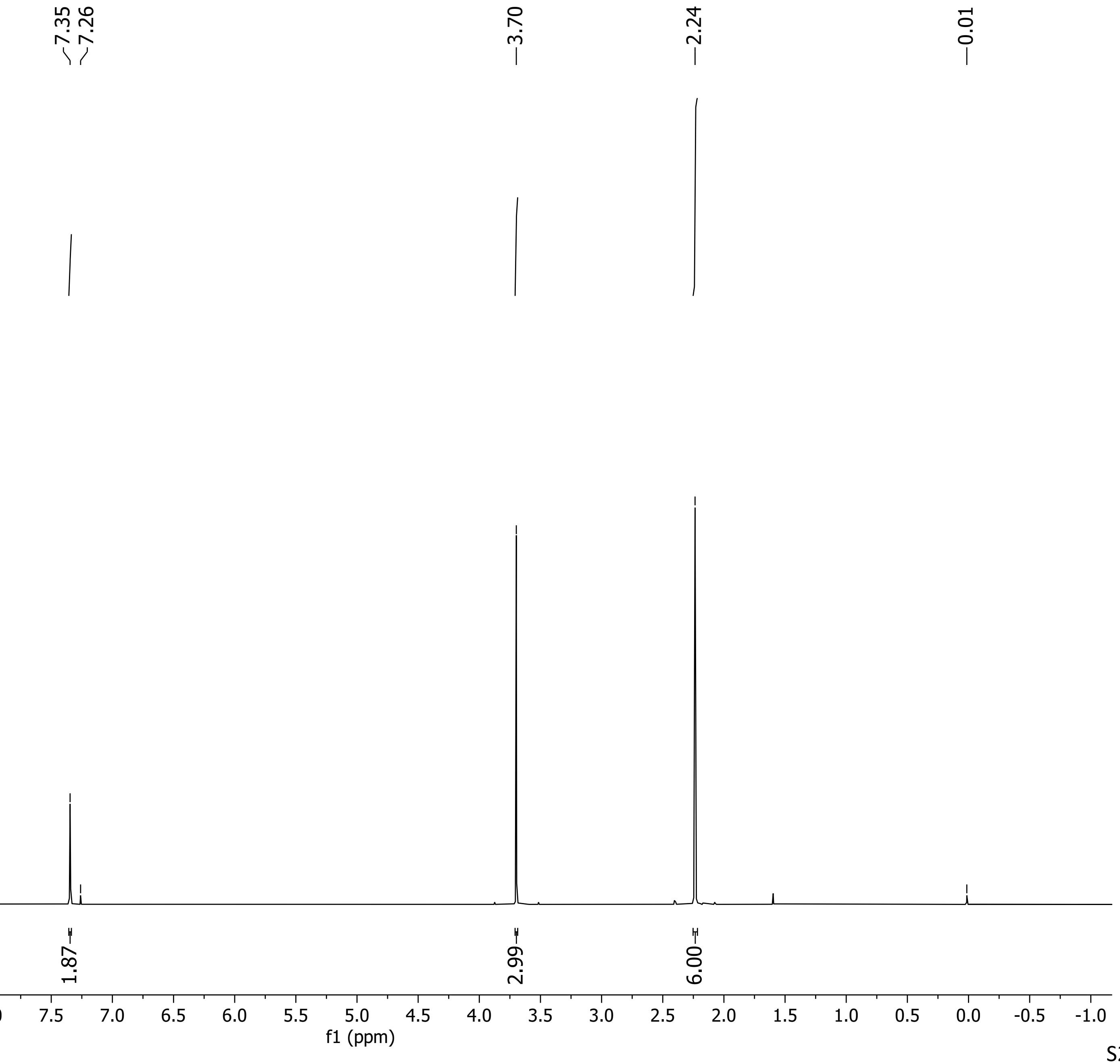

$^{13}C\{^{1}H\}$ NMR (101 MHz, $CDCl_3$)

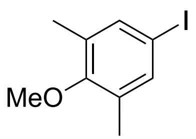

3i

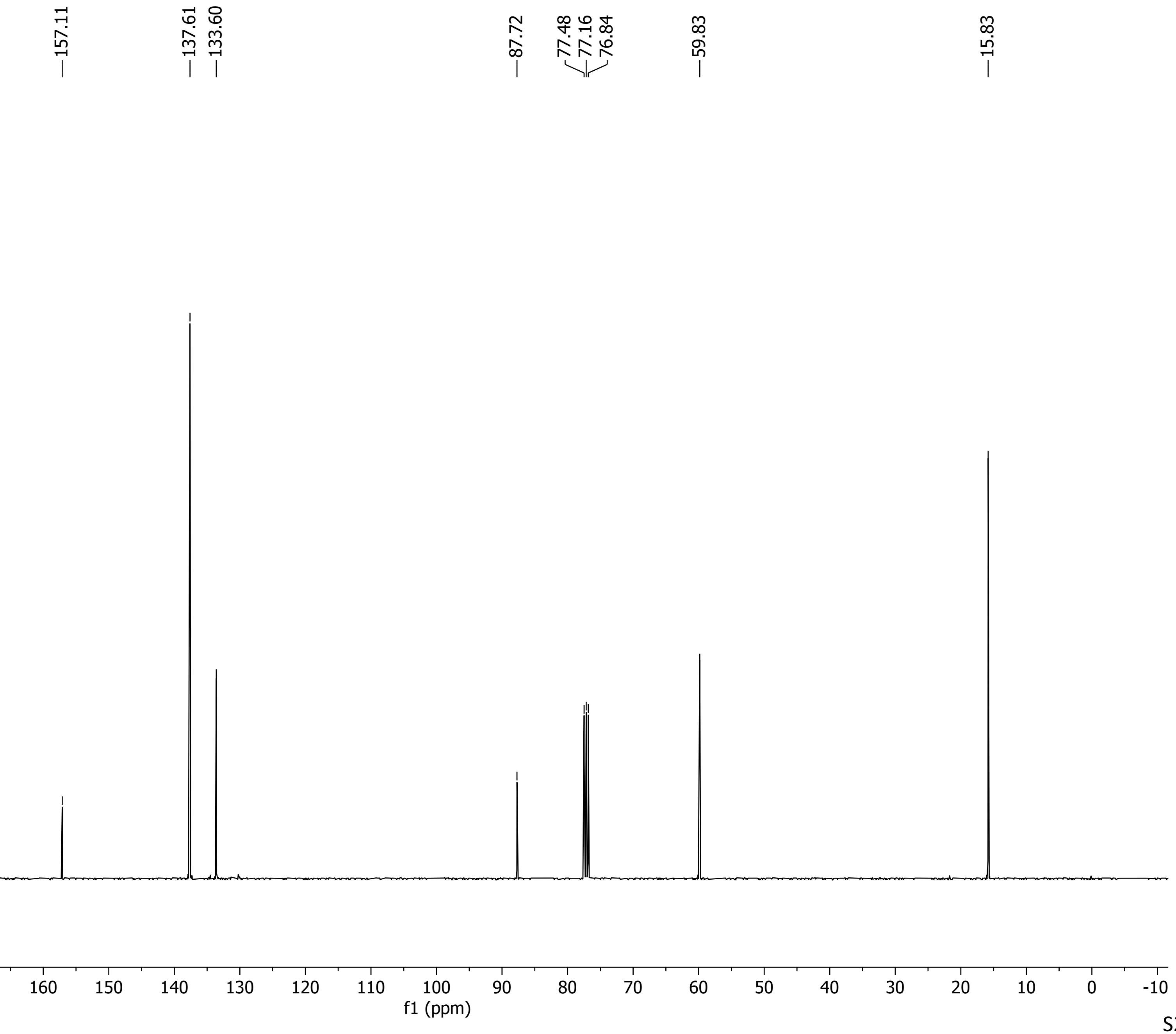

^{1}H NMR (400 MHz, $CDCl_3$)

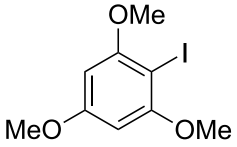

3j

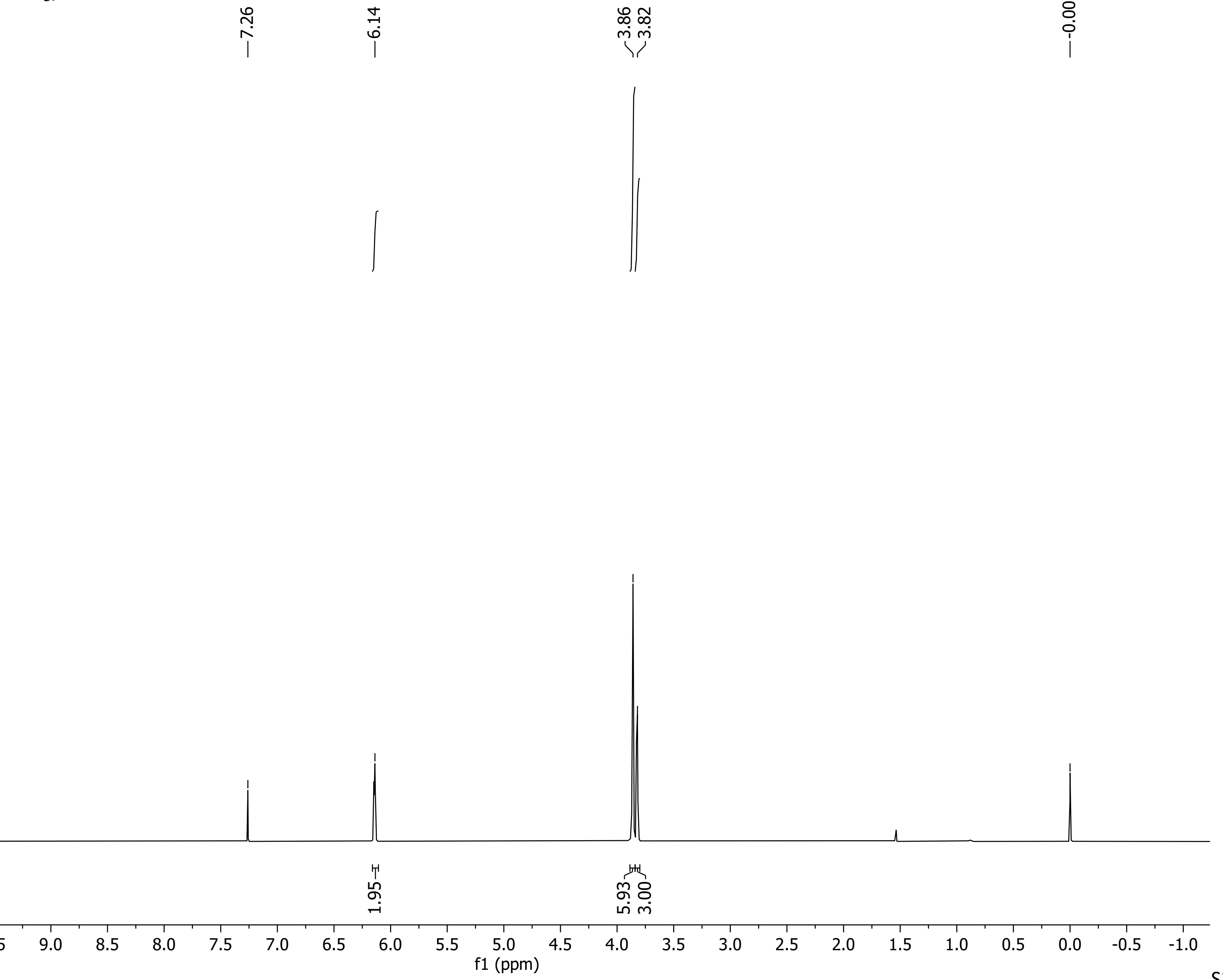

$^{13}C\{^1H\}$ NMR (101 MHz, $CDCl_3$)

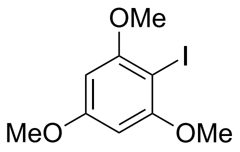

3j

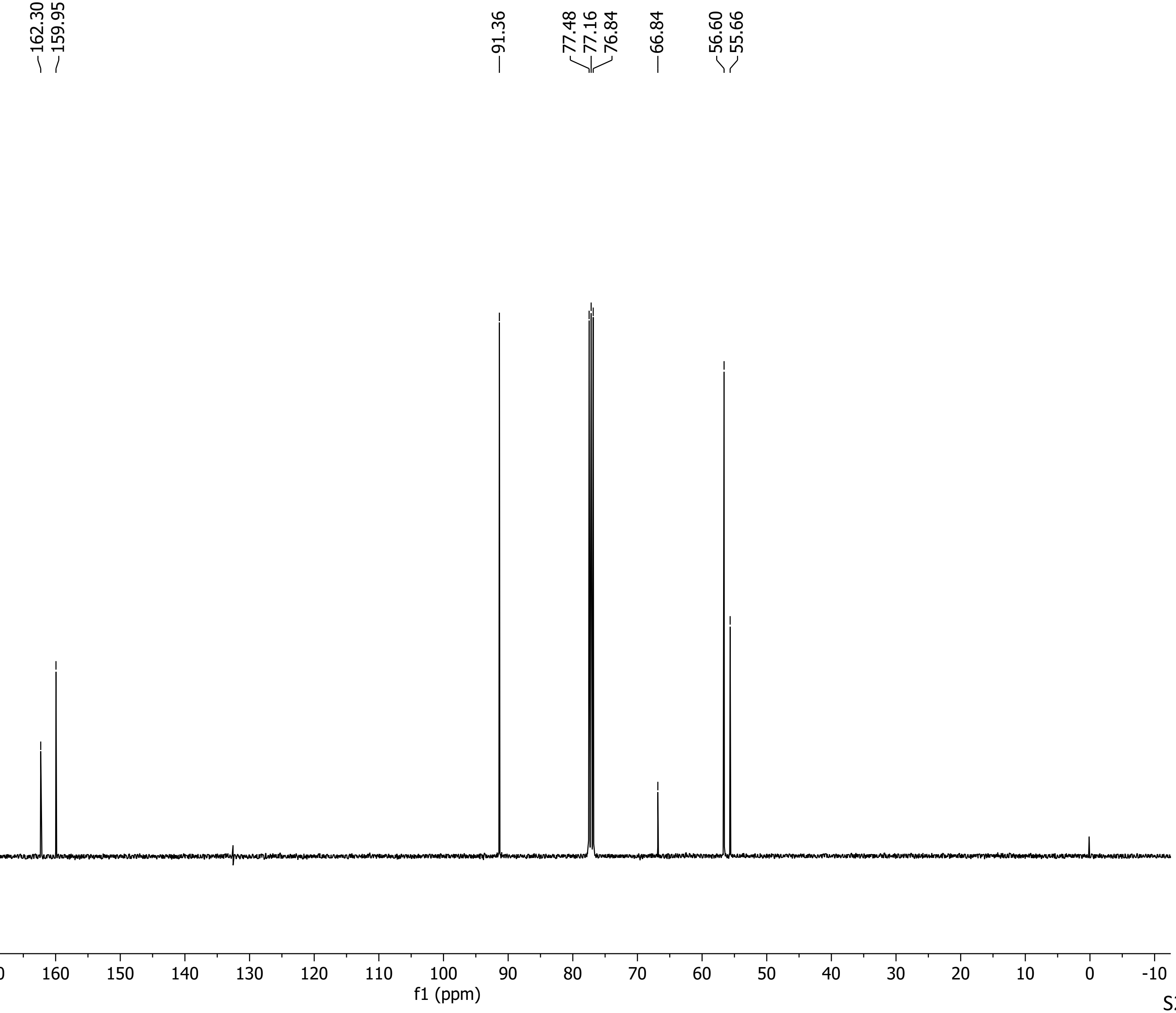

^{1}H NMR (400 MHz, $CDCl_3$)

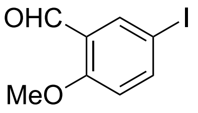

3k

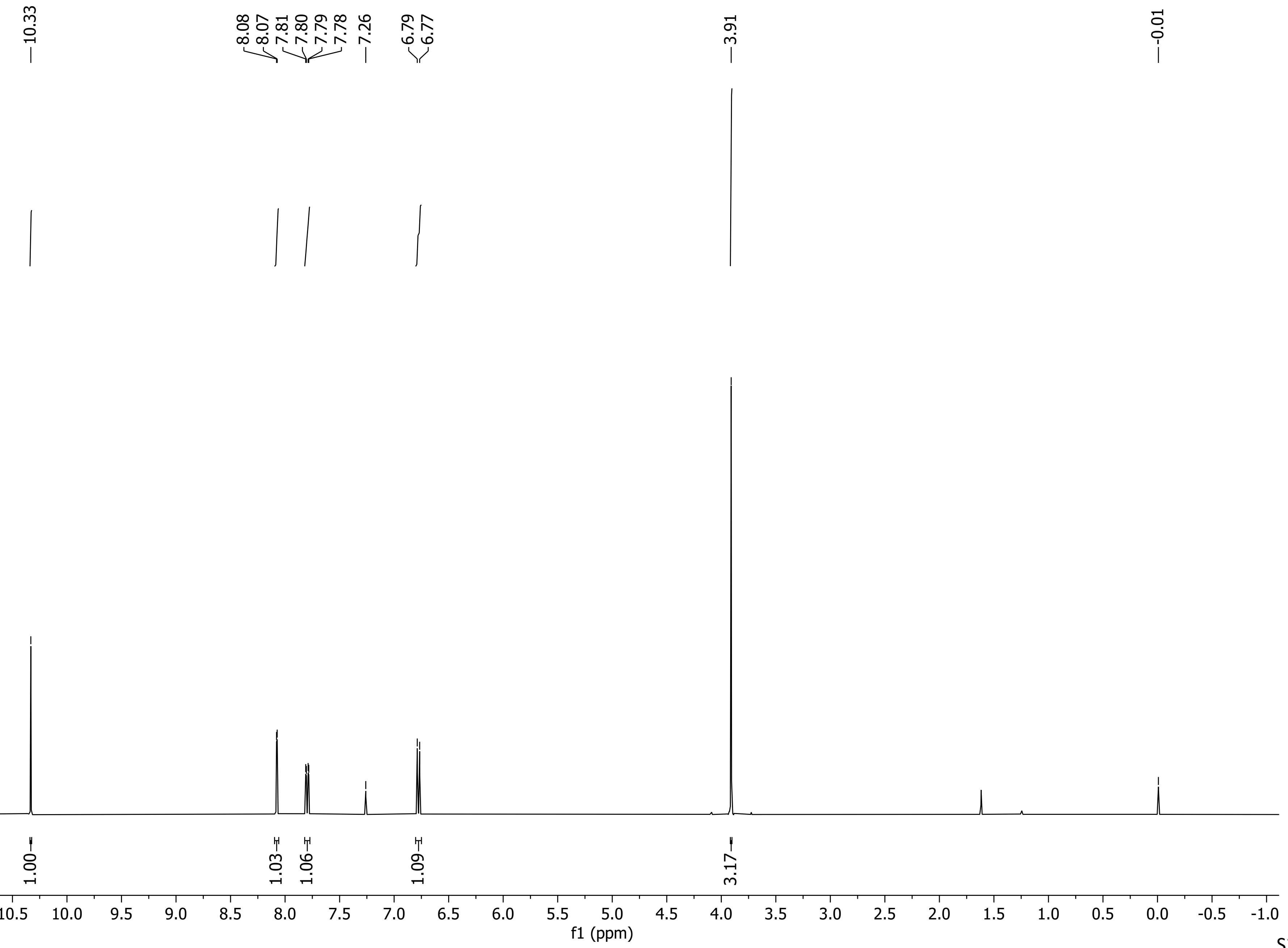

$^{13}C\{^1H\}$ NMR (101 MHz, $CDCl_3$)

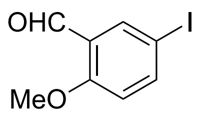

3k

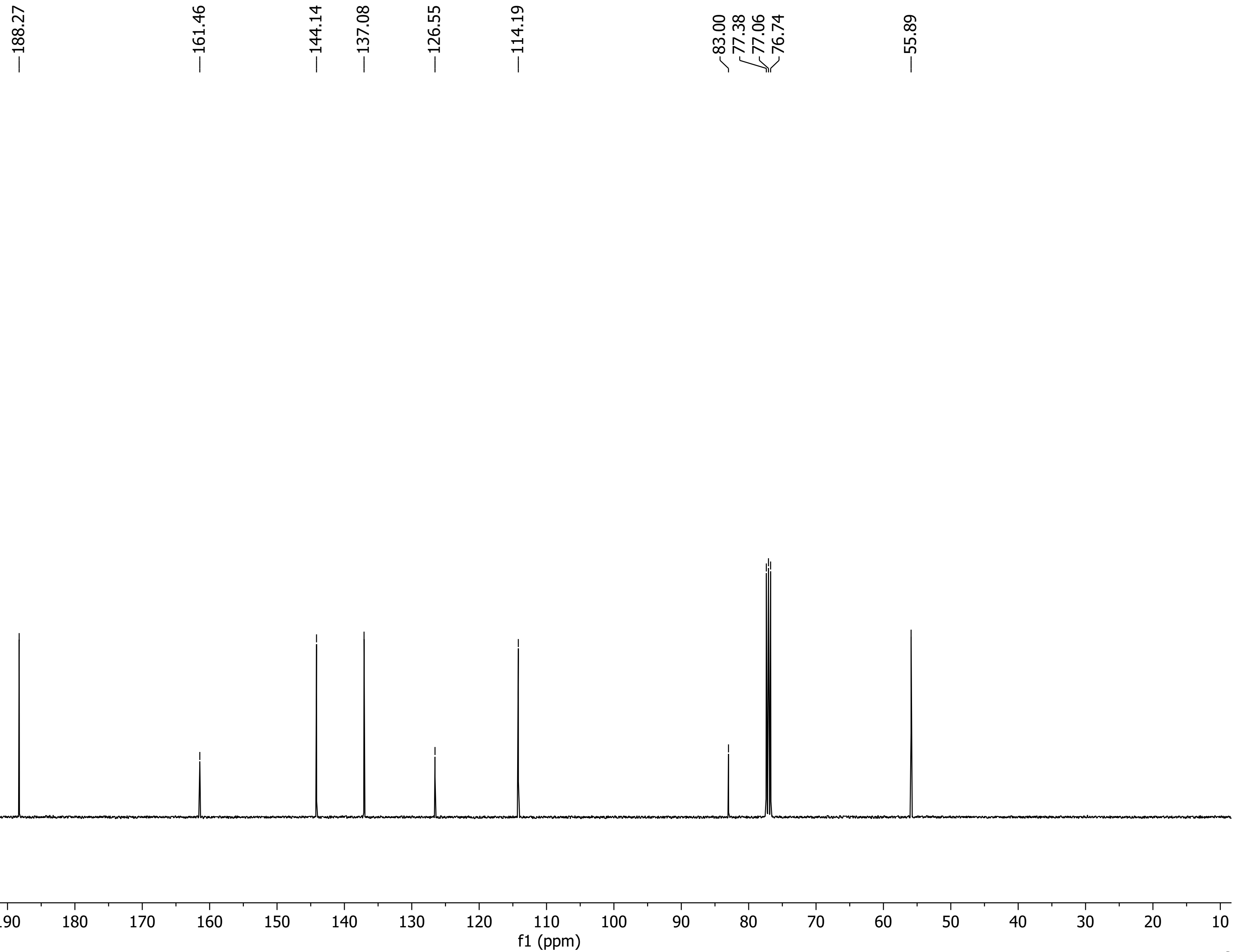

^{1}H NMR (400 MHz, $CDCl_3$)

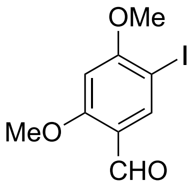

3l

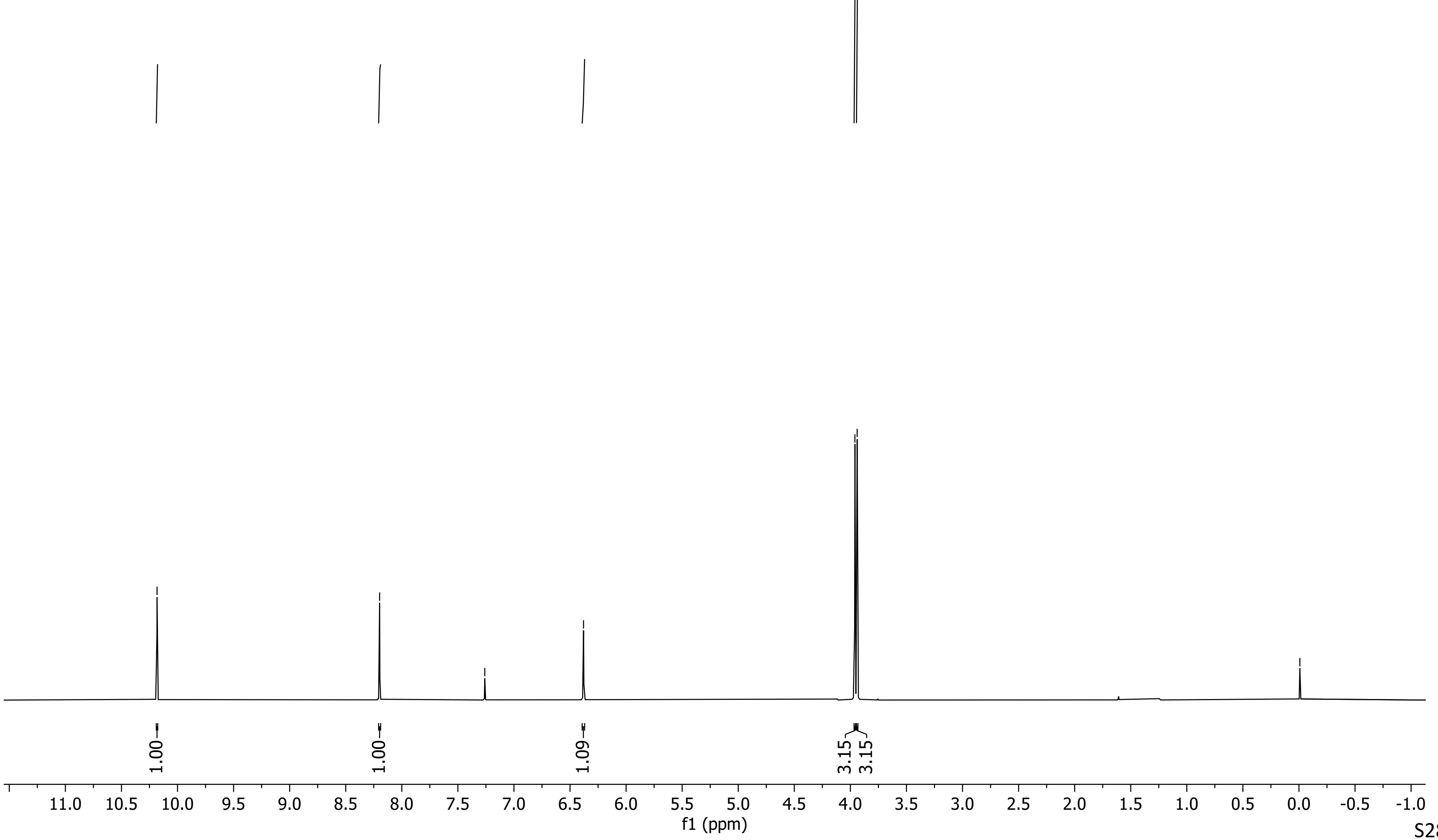

$^{13}C\{^{1}H\}$ NMR (101 MHz, CDCl$_3$)

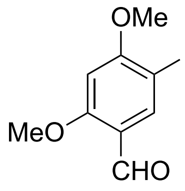

3l

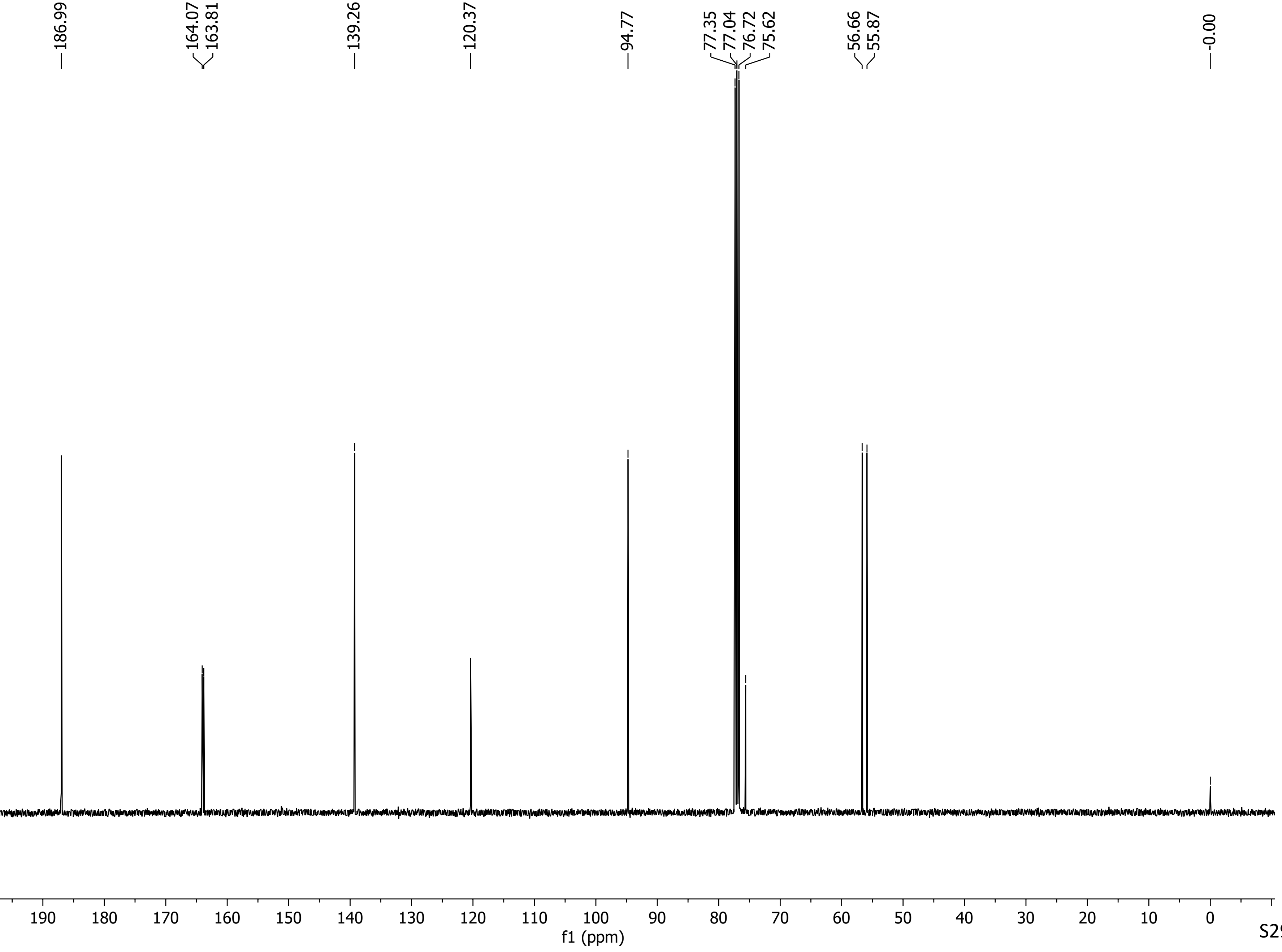

^{1}H NMR (400 MHz, $CDCl_3$)

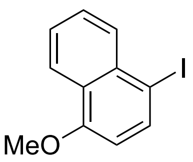

3m

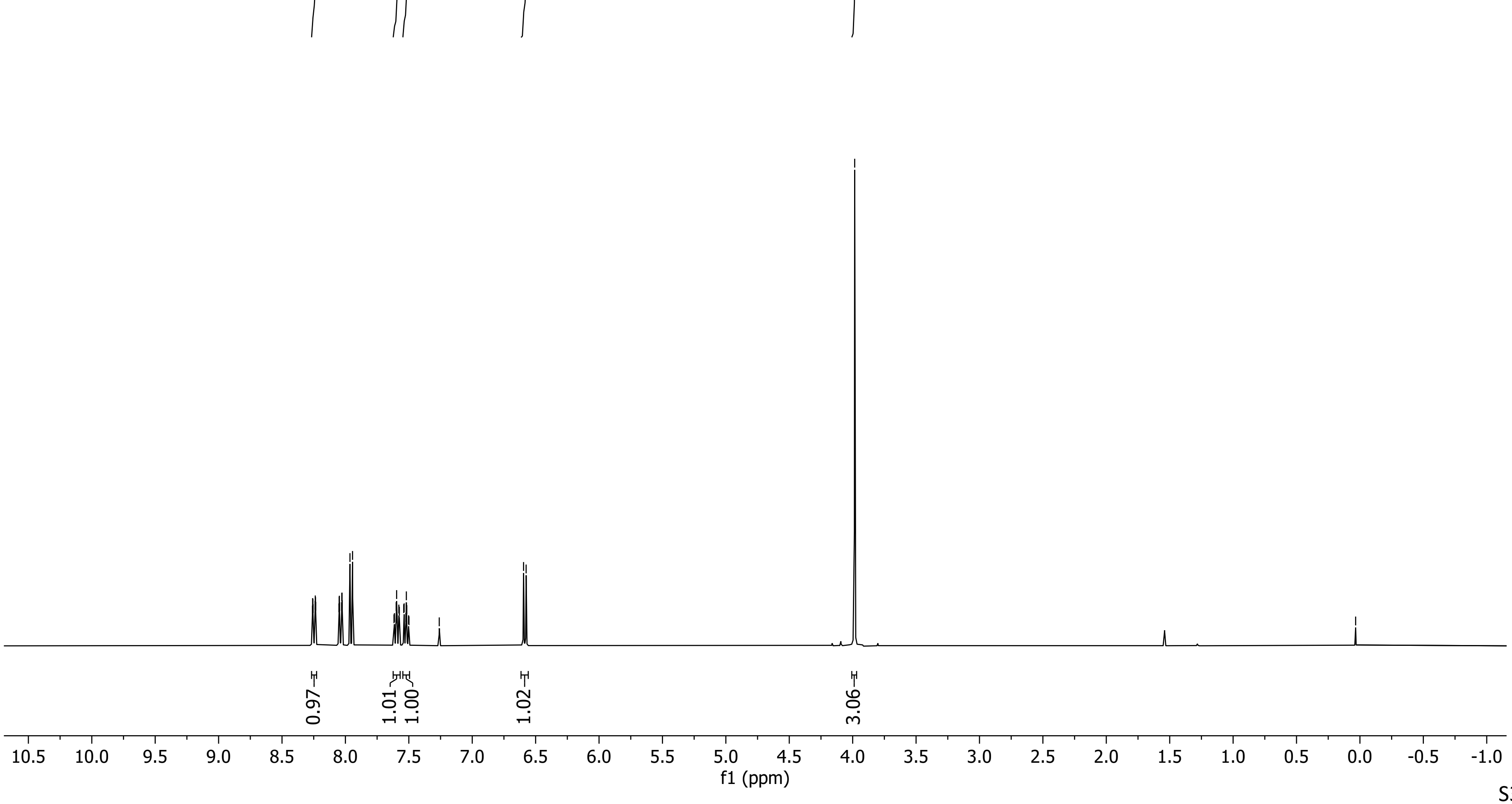

$^{13}C\{^1H\}$ NMR (101 MHz, $CDCl_3$)

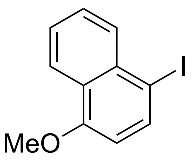

3m

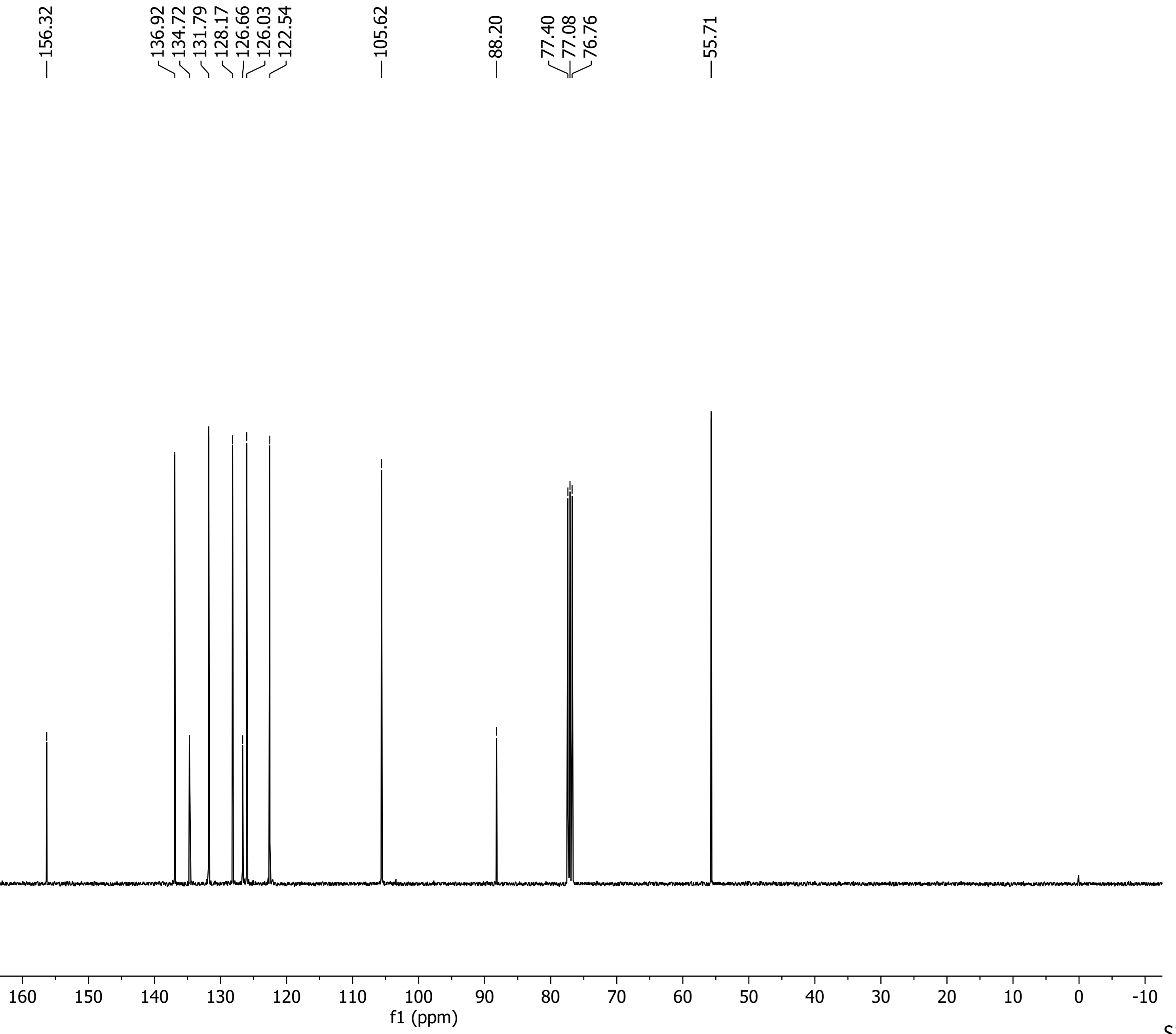

^{1}H NMR (400 MHz, $CDCl_3$)

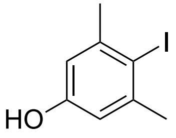

3n

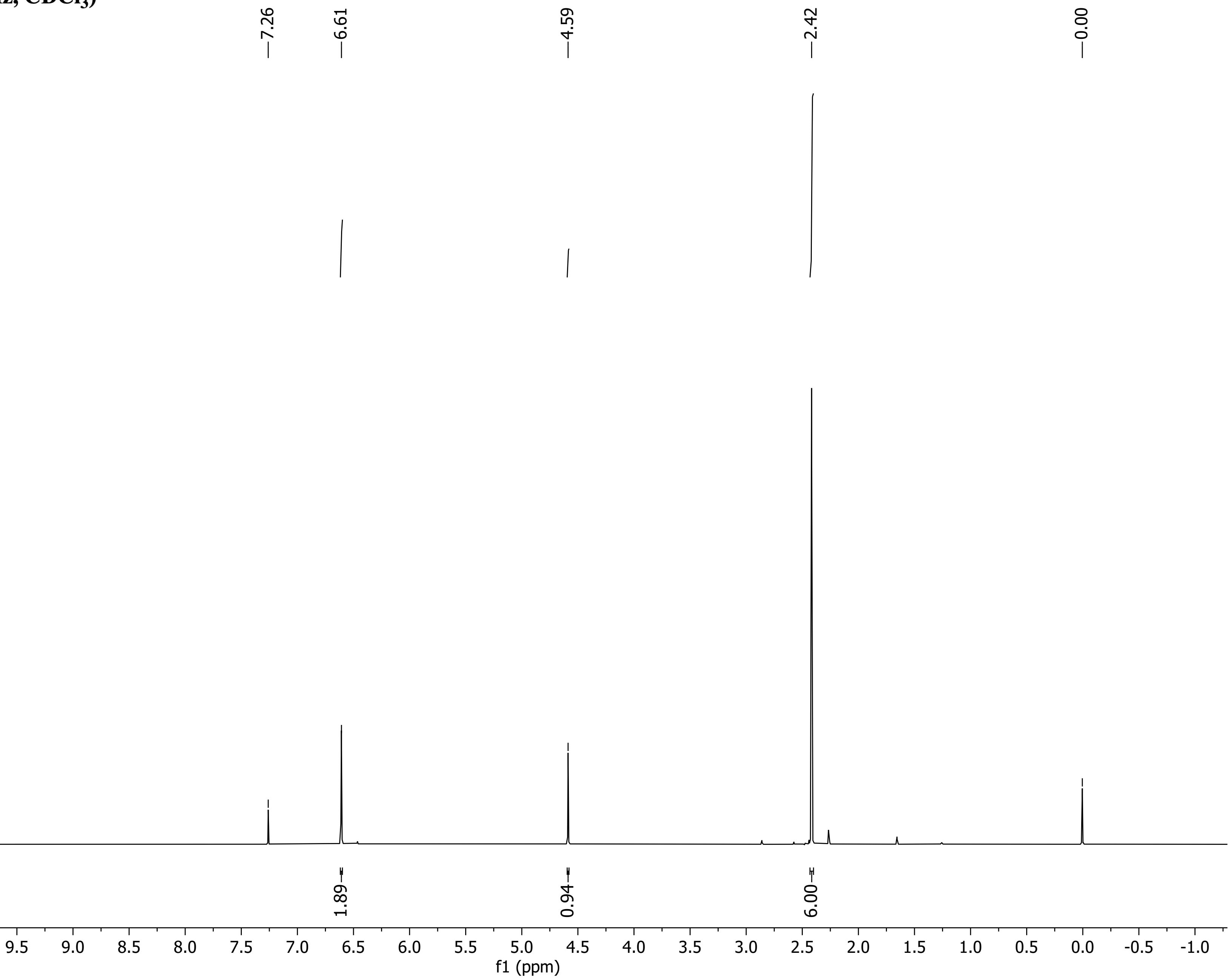

$^{13}C\{^1H\}$ NMR (101 MHz, $CDCl_3$)

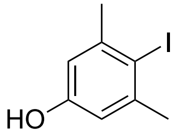

3n

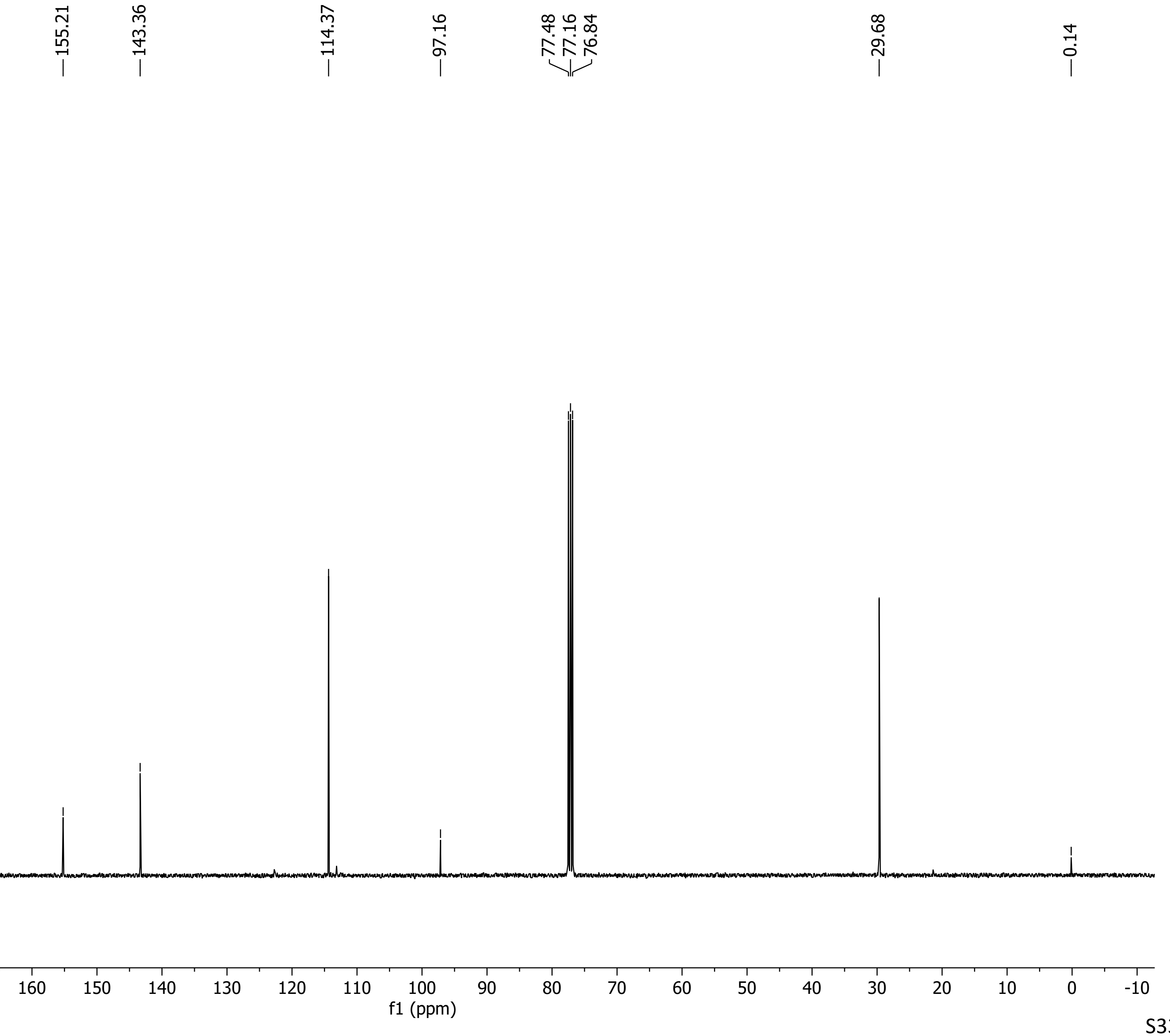

^{1}H NMR (400 MHz, $CDCl_3$)

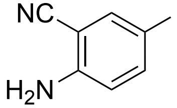

3o

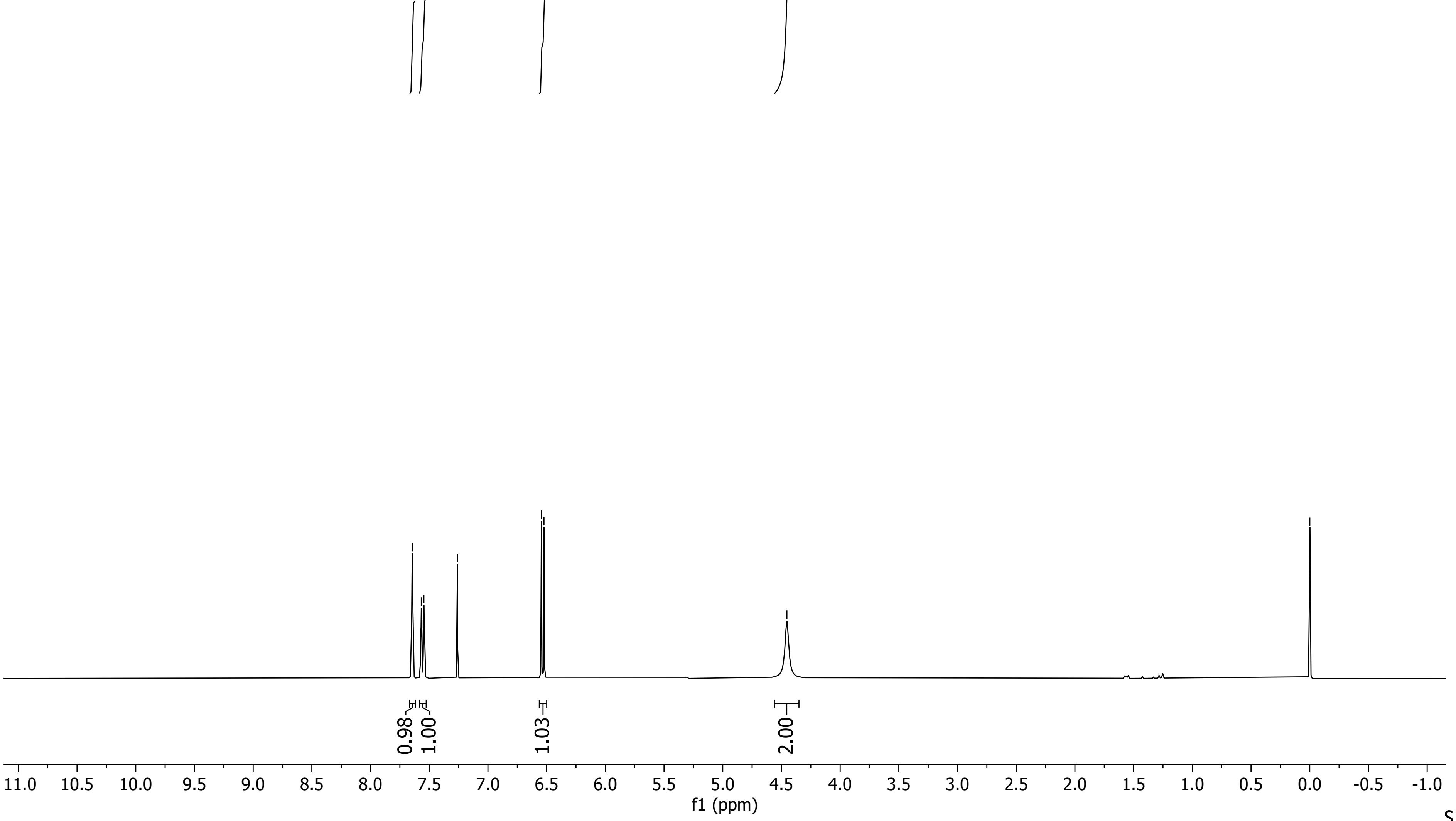

$^{13}C\{^1H\}$ NMR (101 MHz, $CDCl_3$)

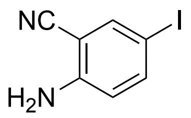

3o

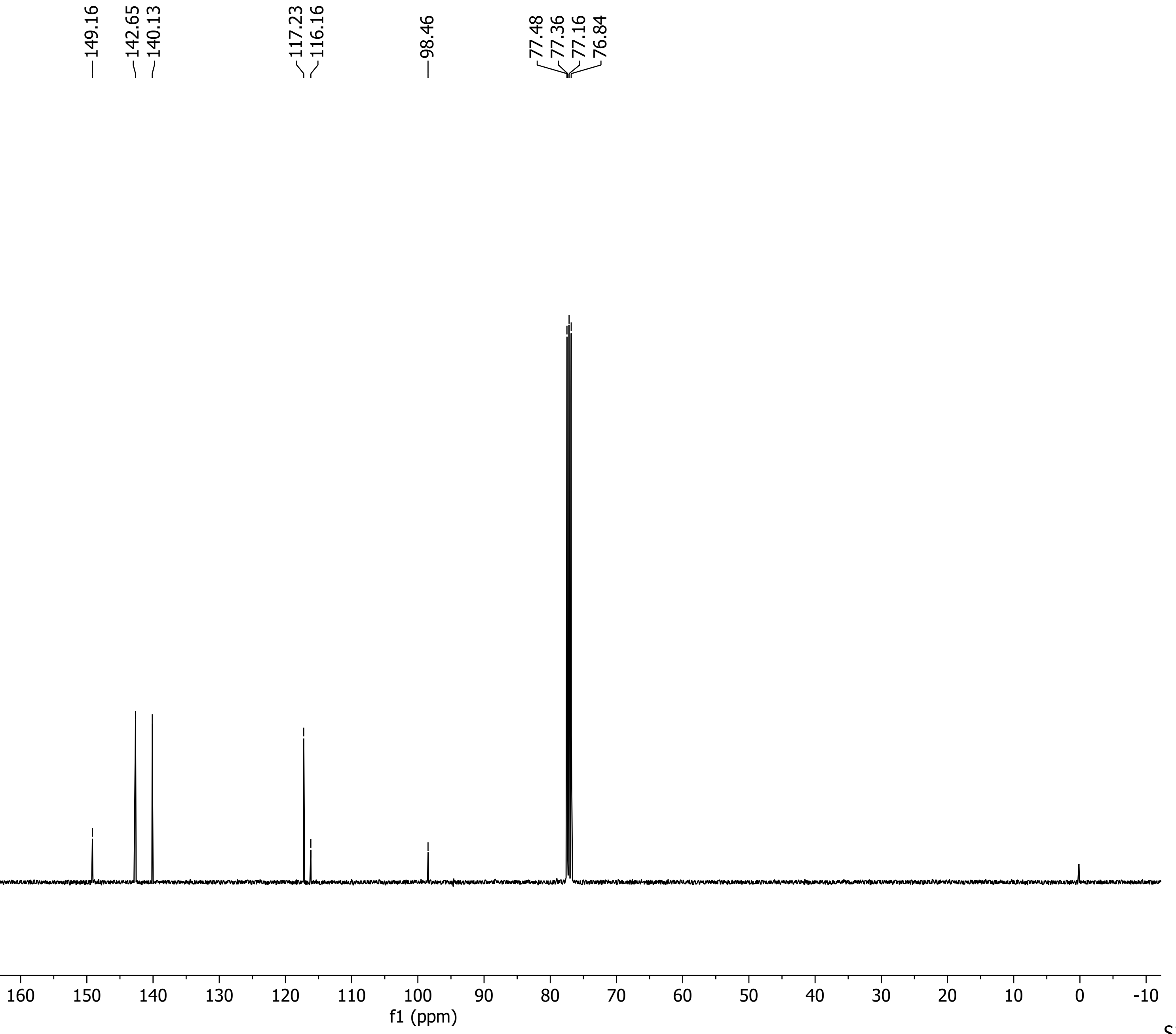

^{1}H NMR (400 MHz, $CDCl_3$)

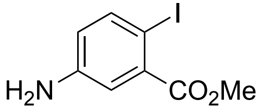

3p

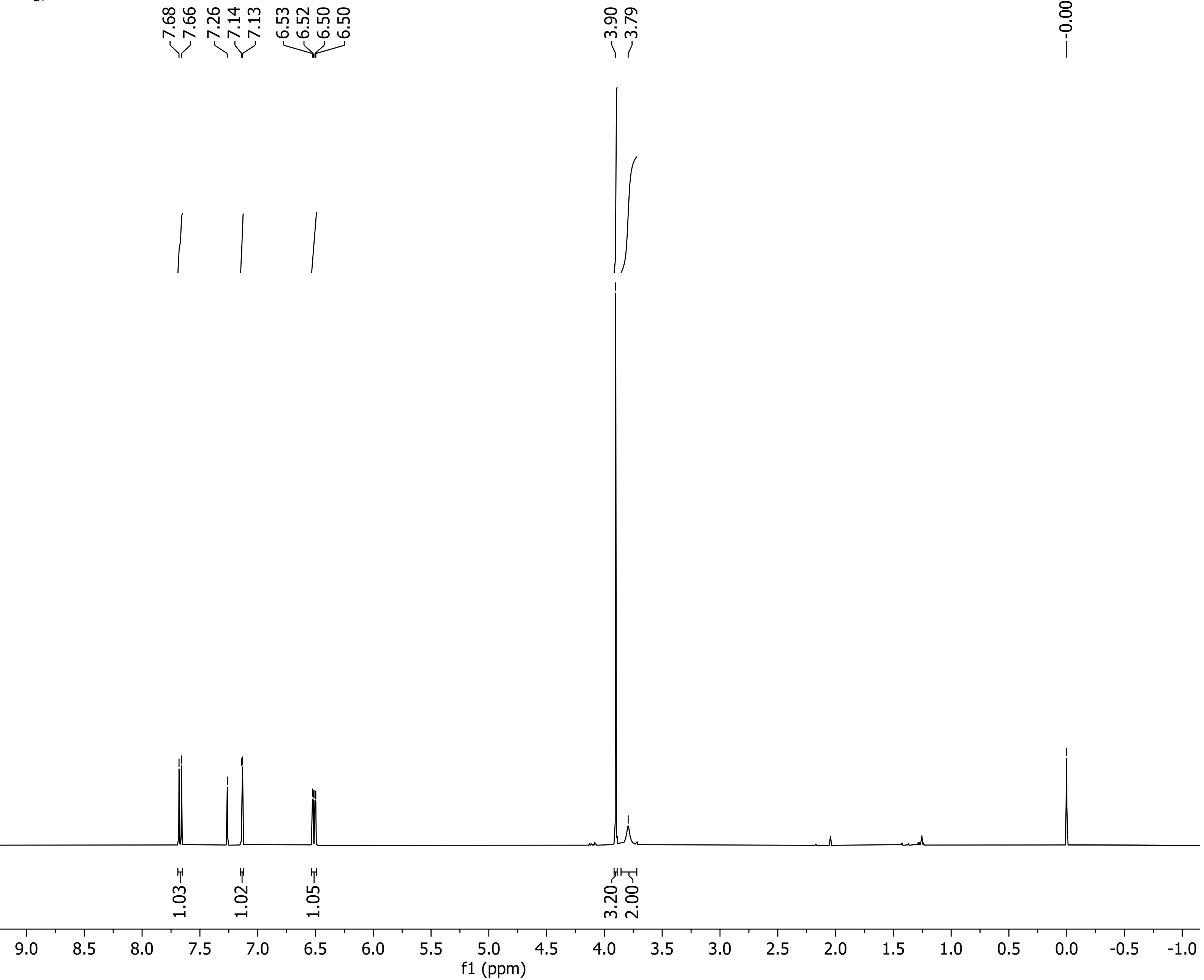

$^{13}C\{^1H\}$ NMR (101 MHz, $CDCl_3$)

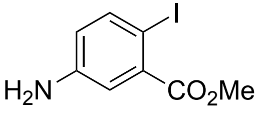

3p

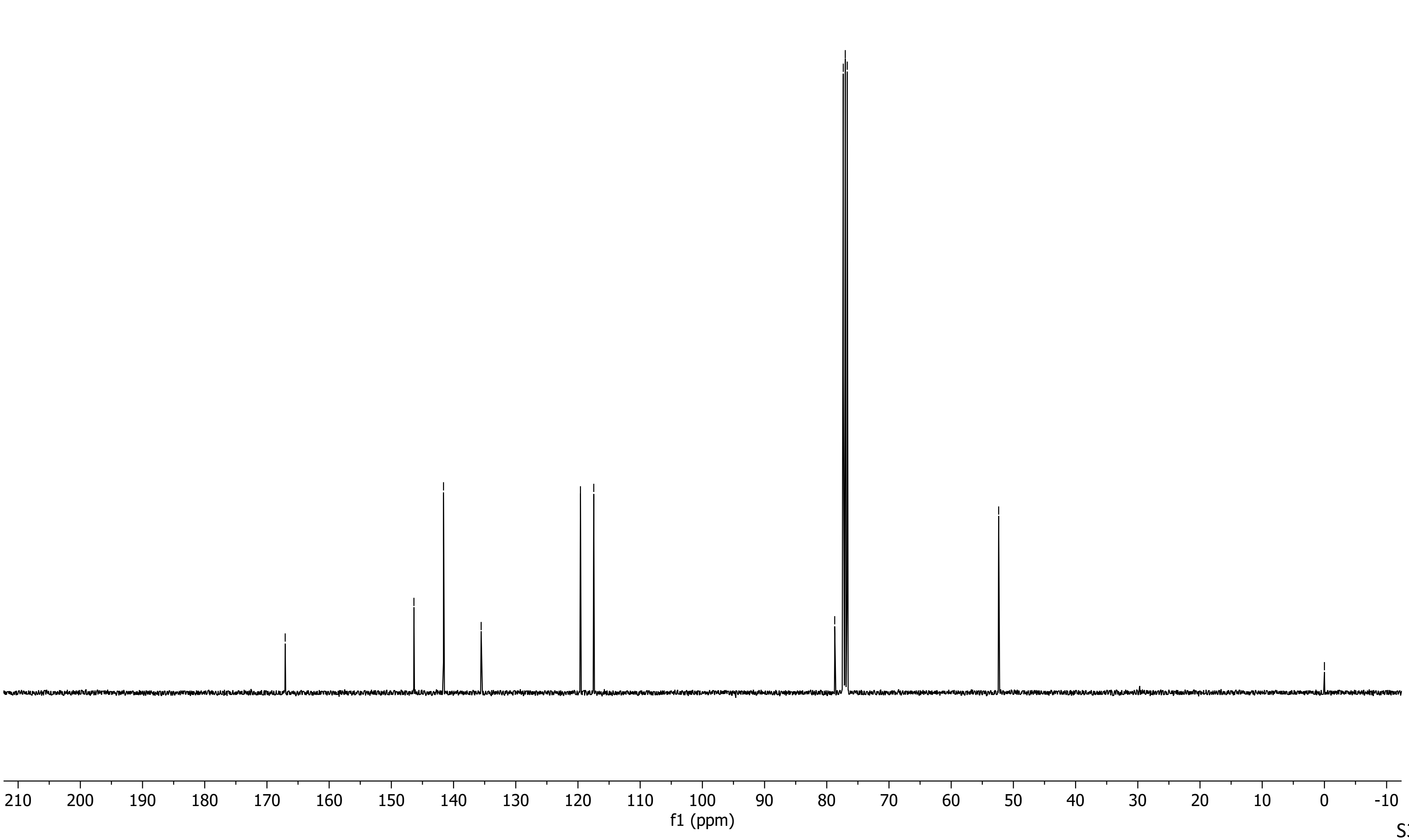

^{1}H NMR (400 MHz, $CDCl_3$)

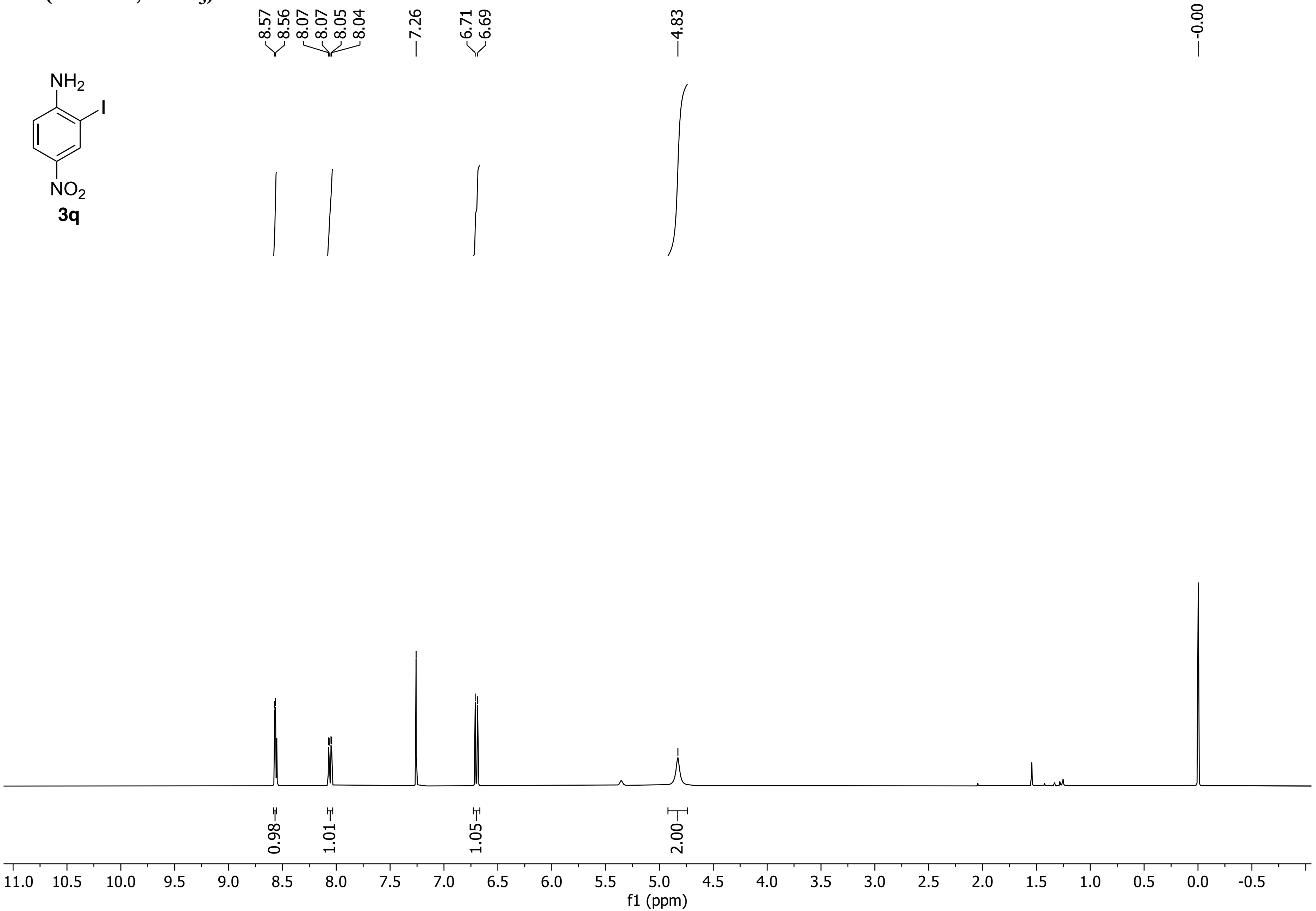

$^{13}C\{^1H\}$ NMR (101 MHz, $CDCl_3$)

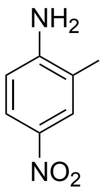

3q

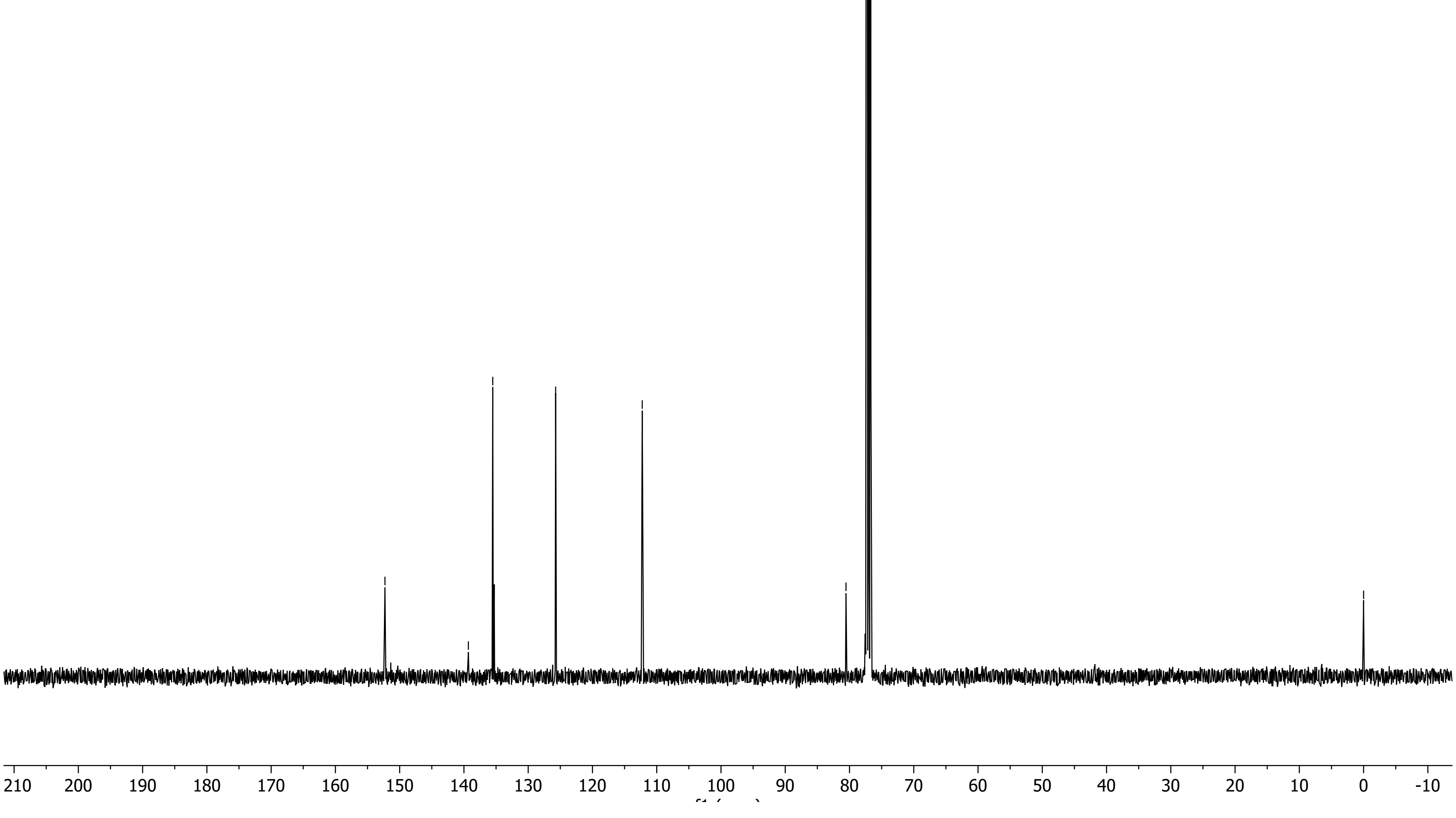

^{1}H NMR (400 MHz, $CDCl_3$)

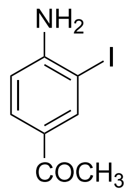

3r

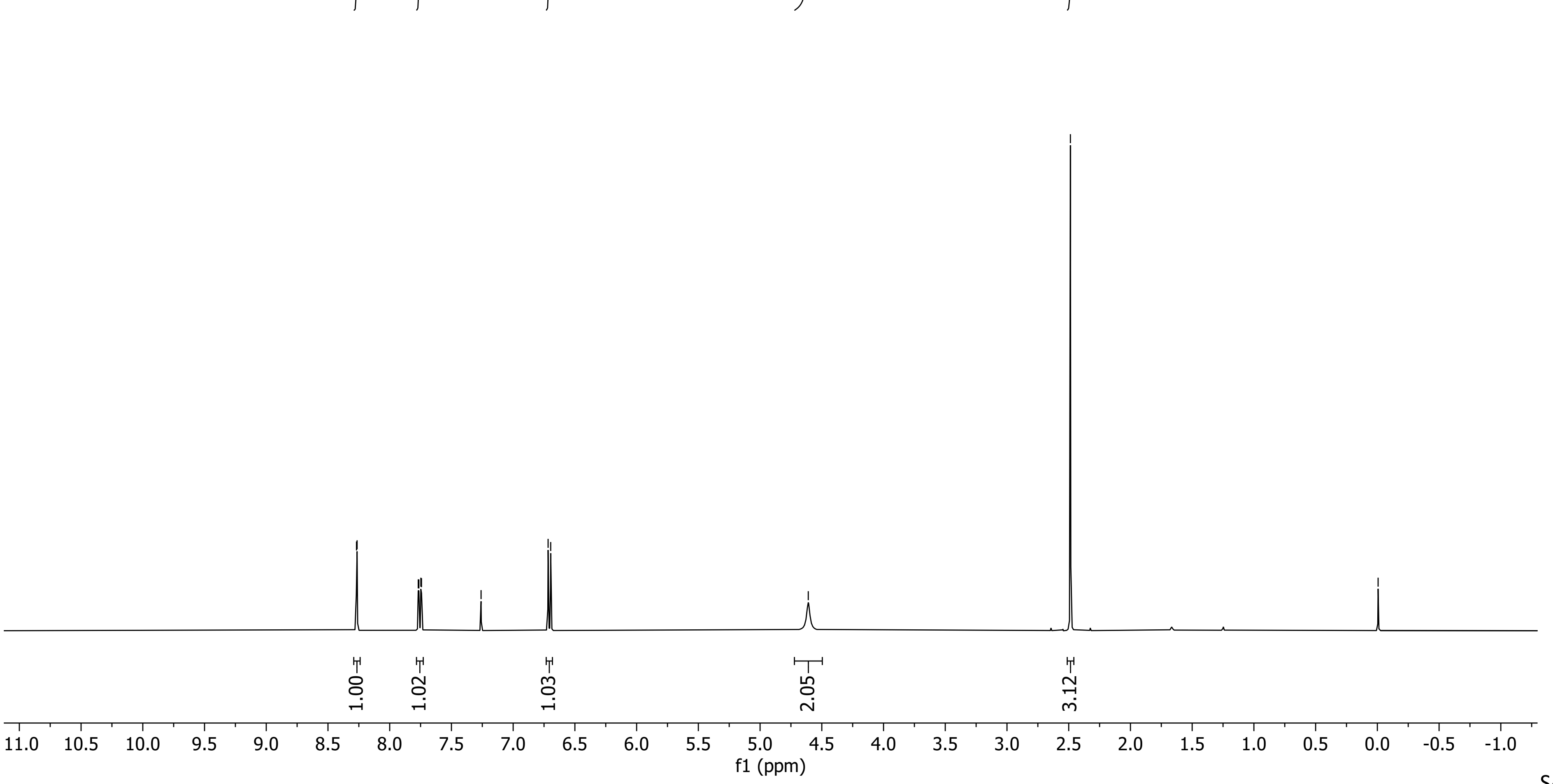

$^{13}C\{^{1}H\}$ NMR (101 MHz, $CDCl_3$)

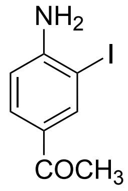

3r

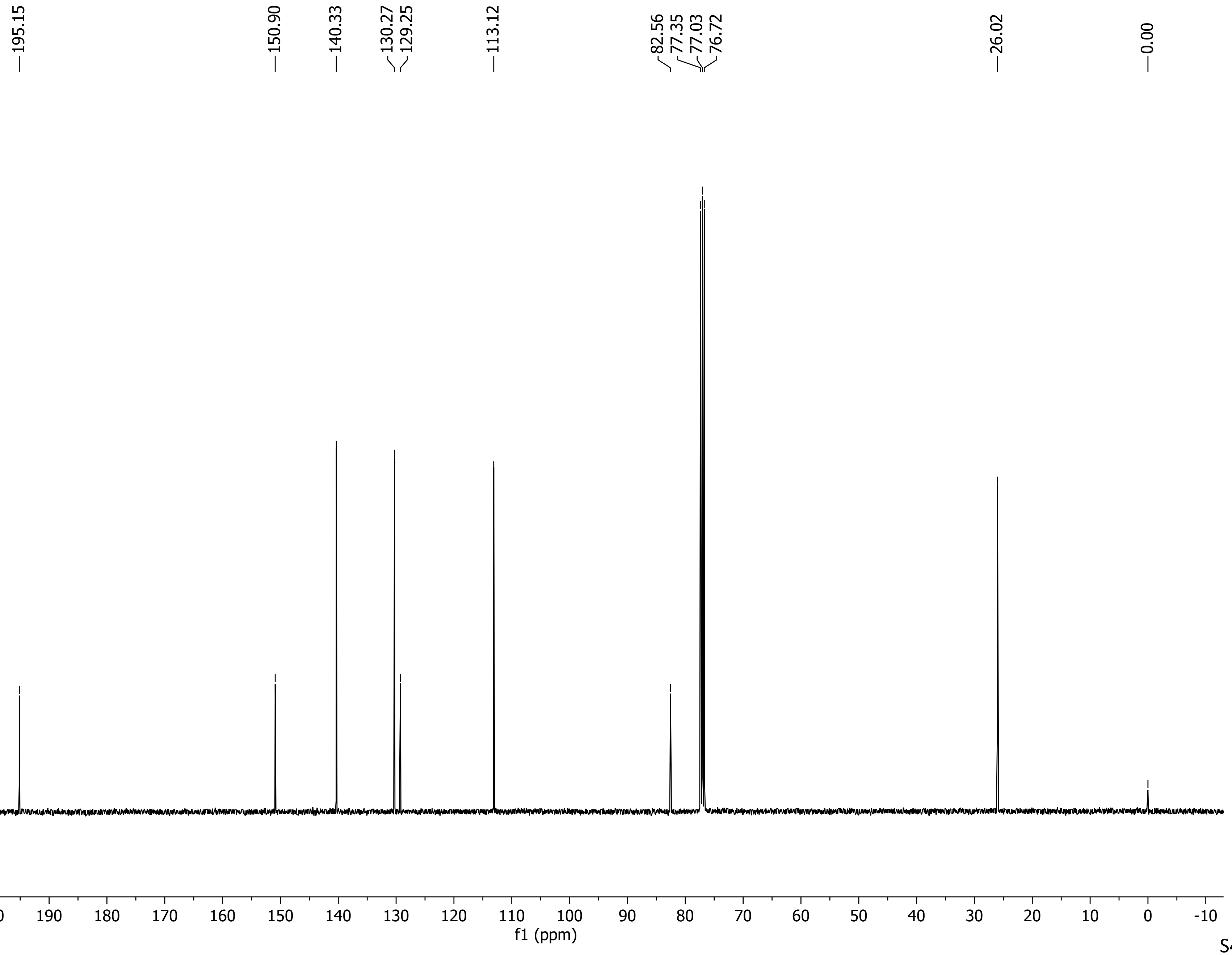

^{1}H NMR (400 MHz, $CDCl_3$)

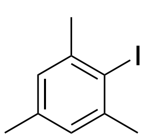

3s

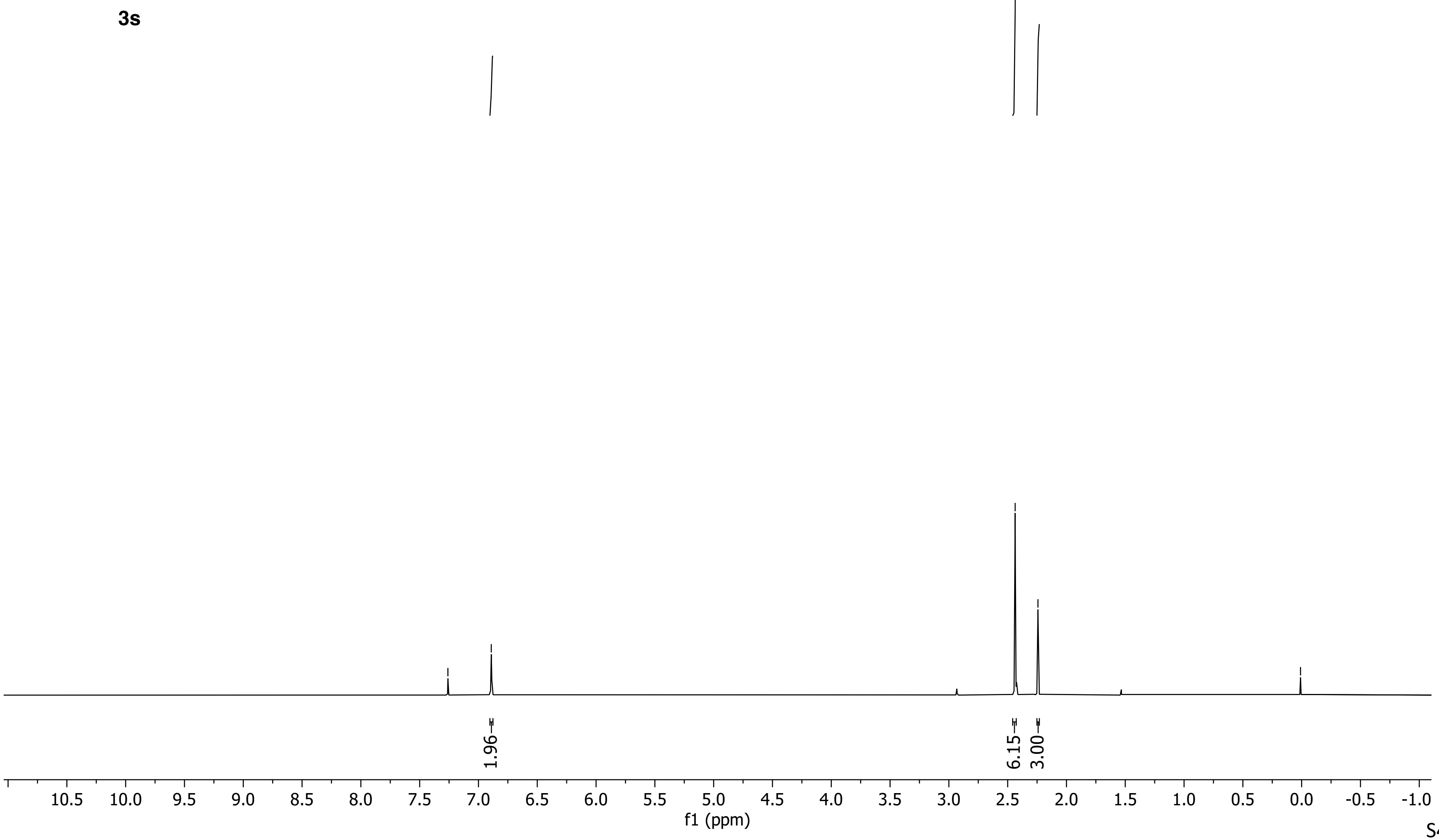

$^{13}C\{^1H\}$ NMR (101 MHz, $CDCl_3$)

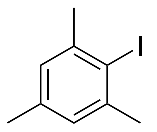

3s

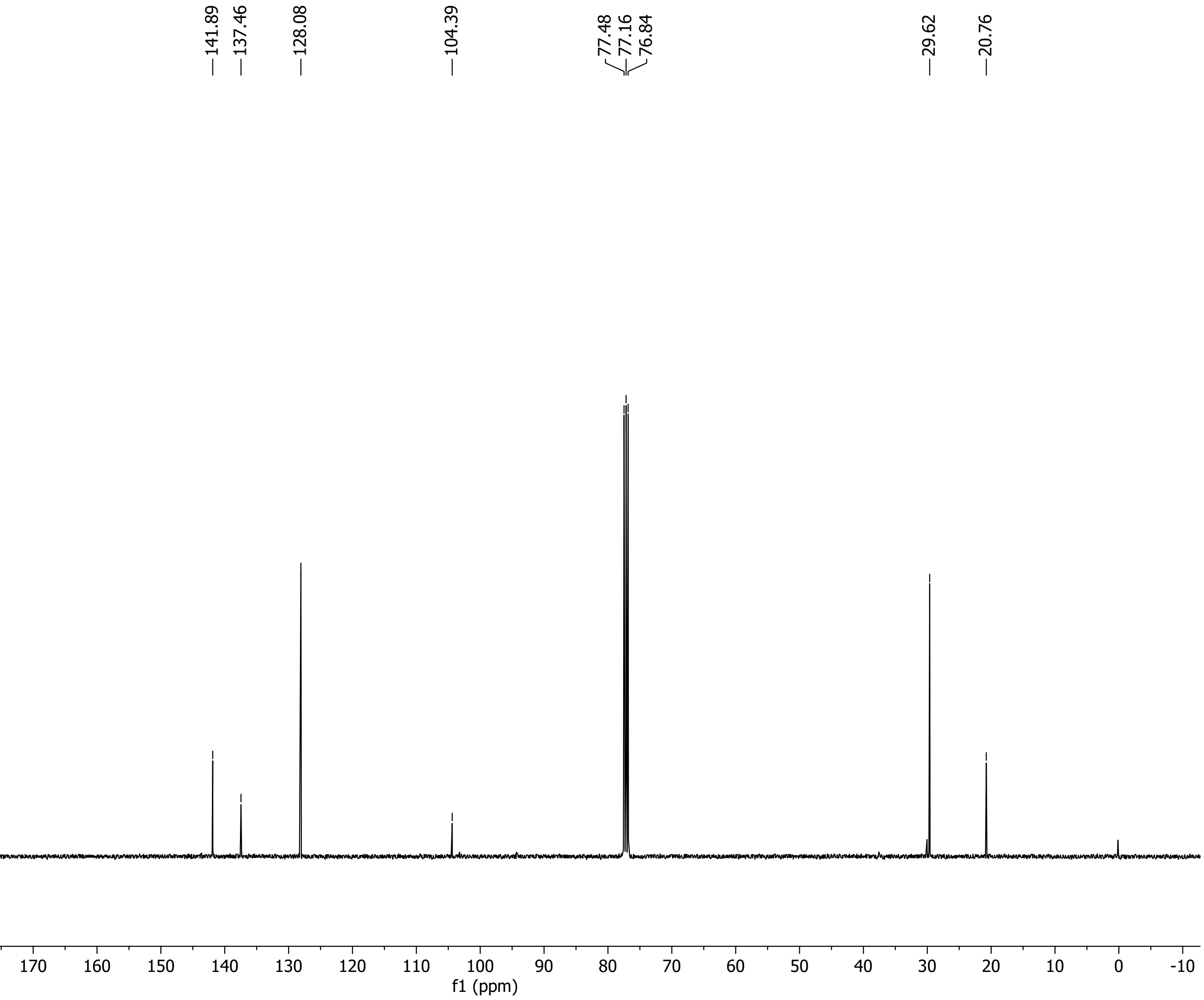

^{1}H NMR (400 MHz, $CDCl_3$)

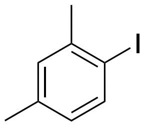

3t

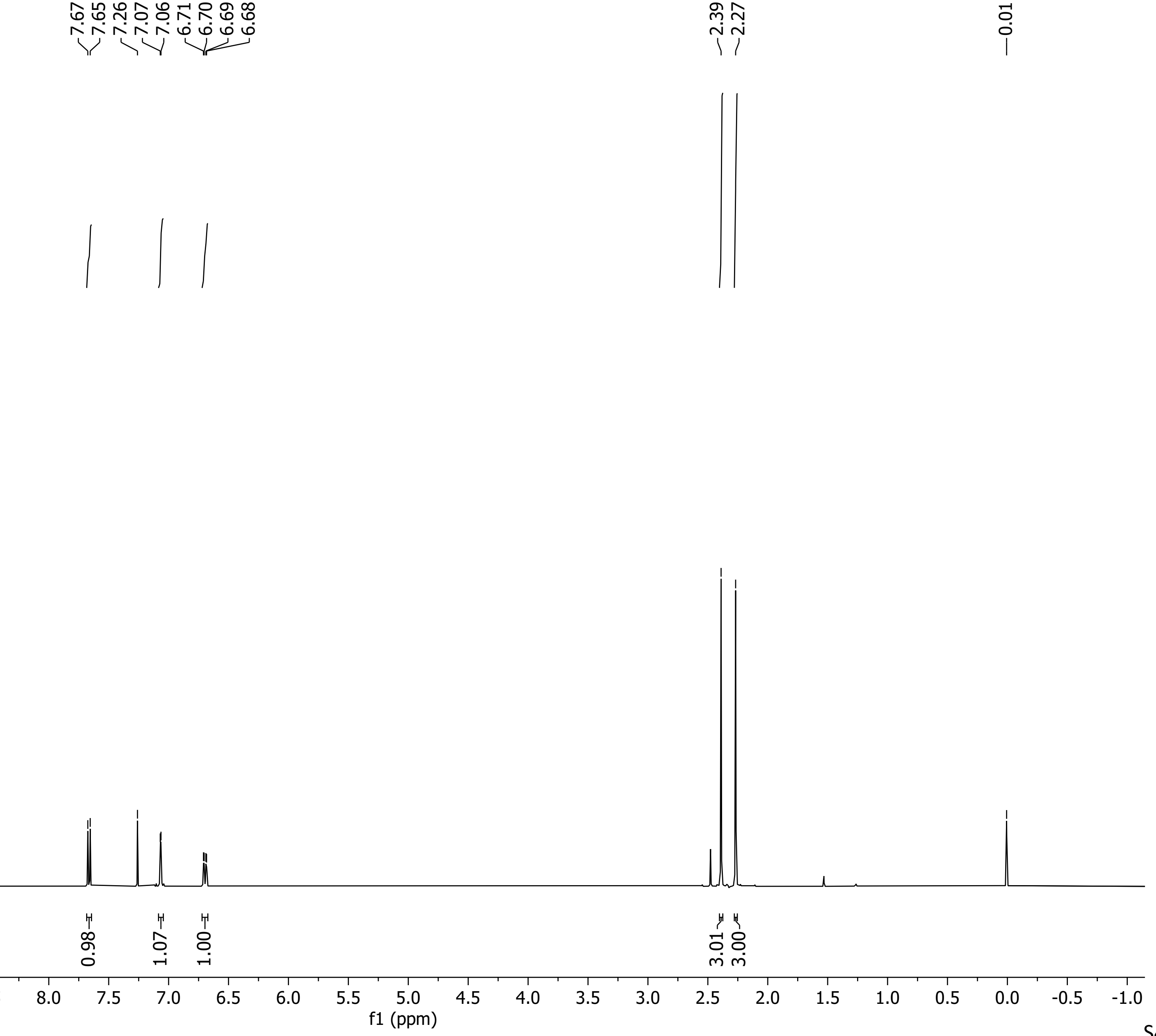

$^{13}C\{^{1}H\}$ NMR (101 MHz, $CDCl_3$)

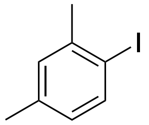

3t

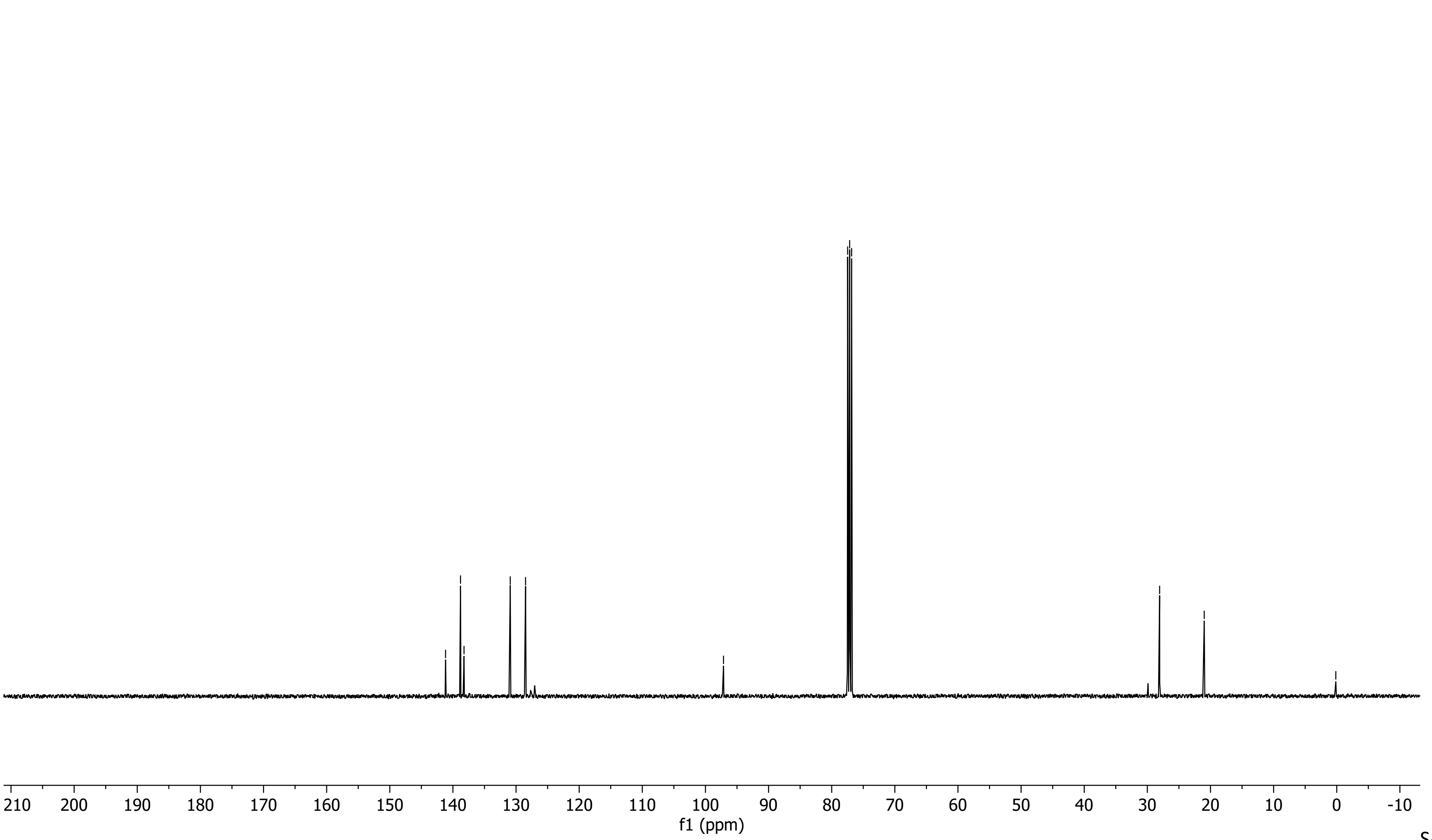

^{1}H NMR (400 MHz, $CDCl_3$)

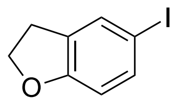

3u

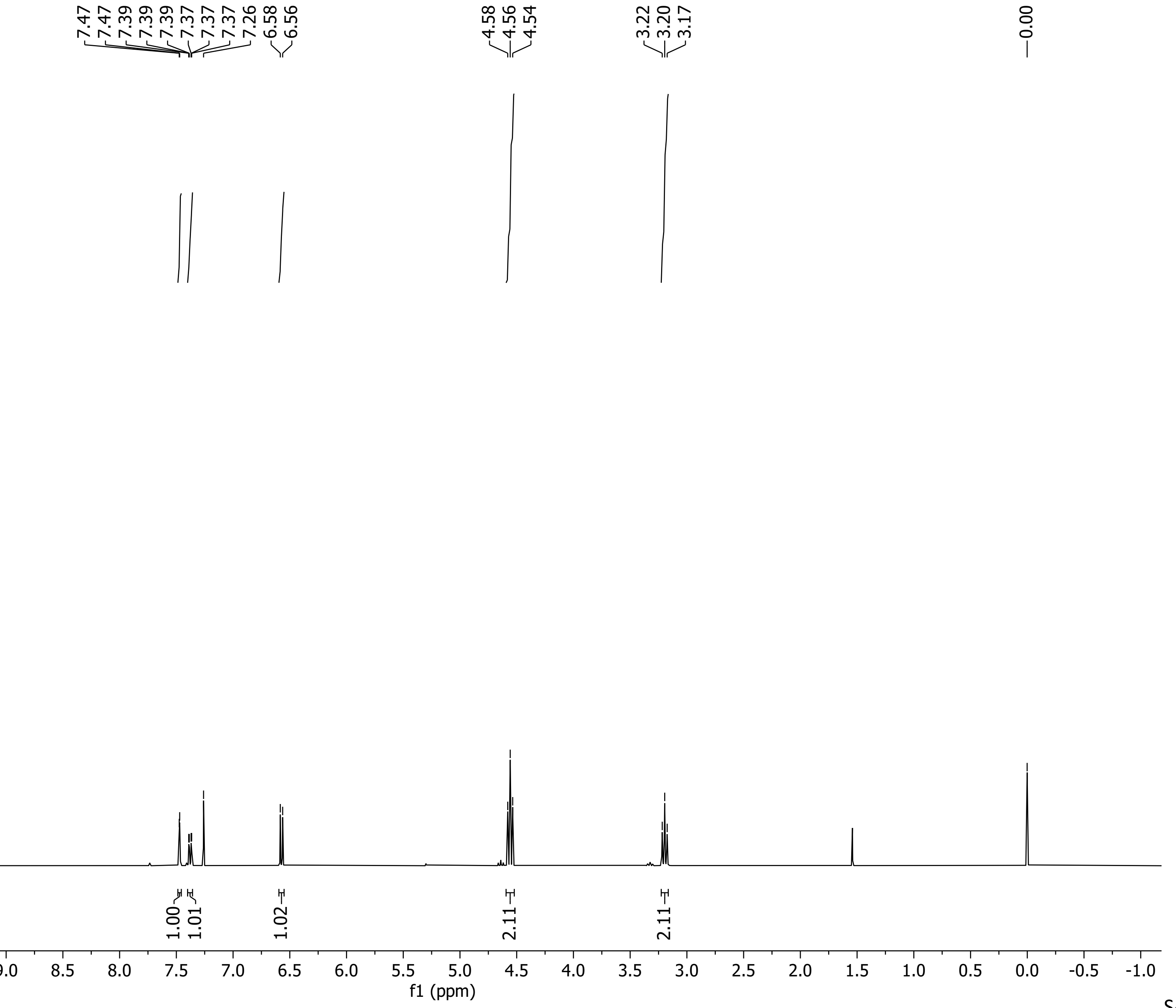

$^{13}C\{^{1}H\}$ NMR (101 MHz, $CDCl_3$)

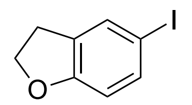

3u

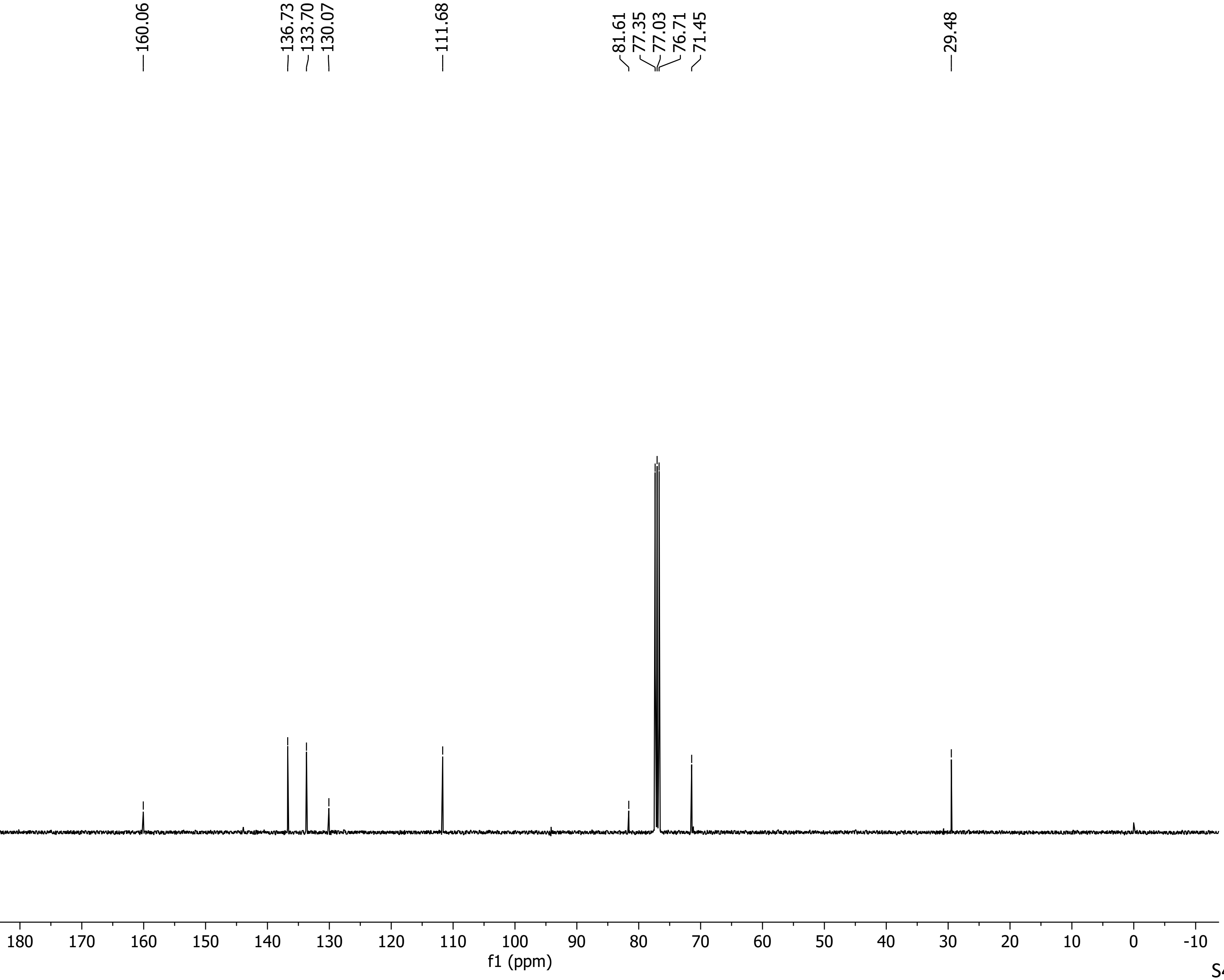

^{1}H NMR (400 MHz, CDCl$_3$)

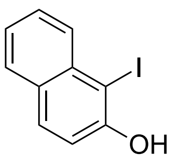

3w

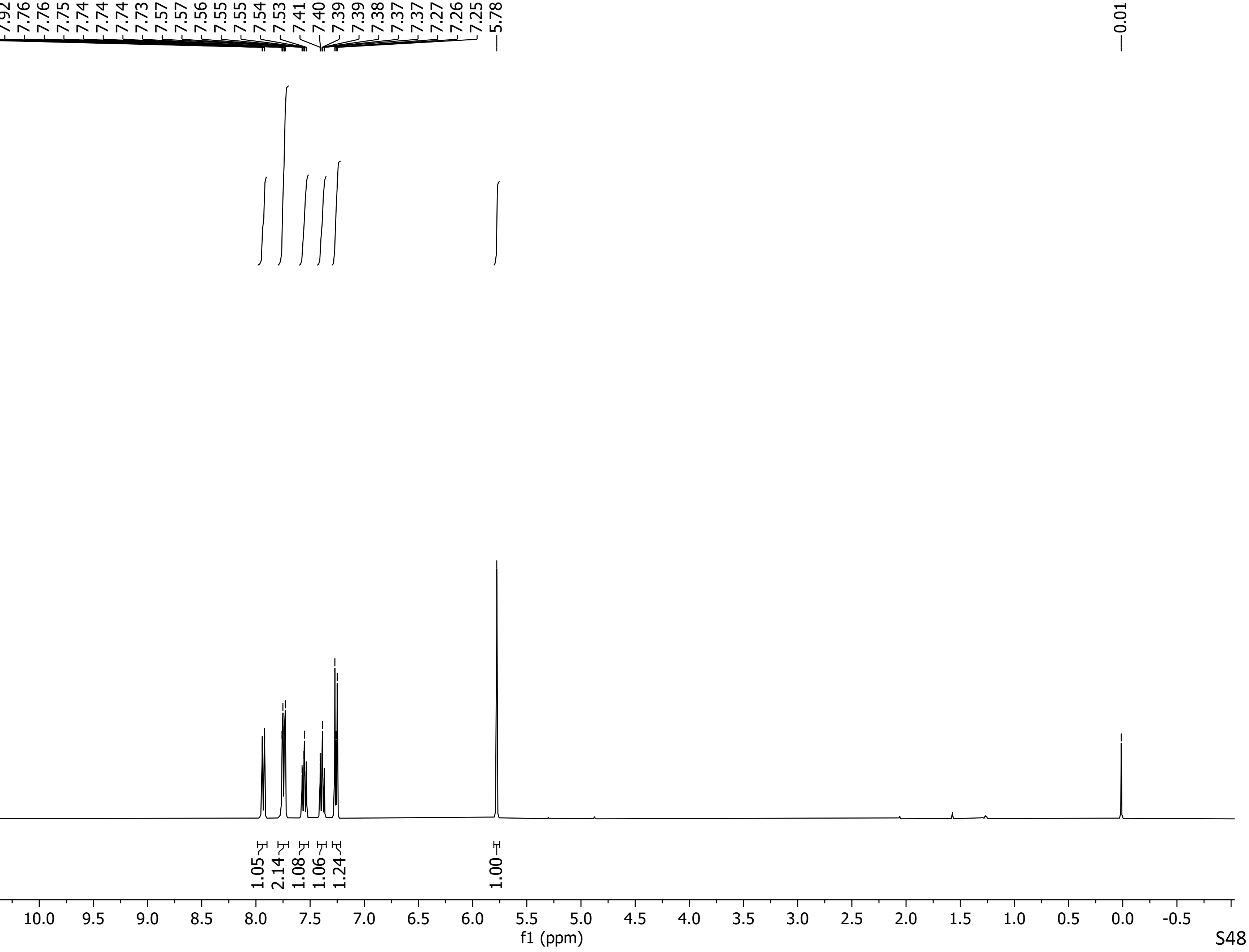

$^{13}C\{^{1}H\}$ NMR (101 MHz, $CDCl_3$)

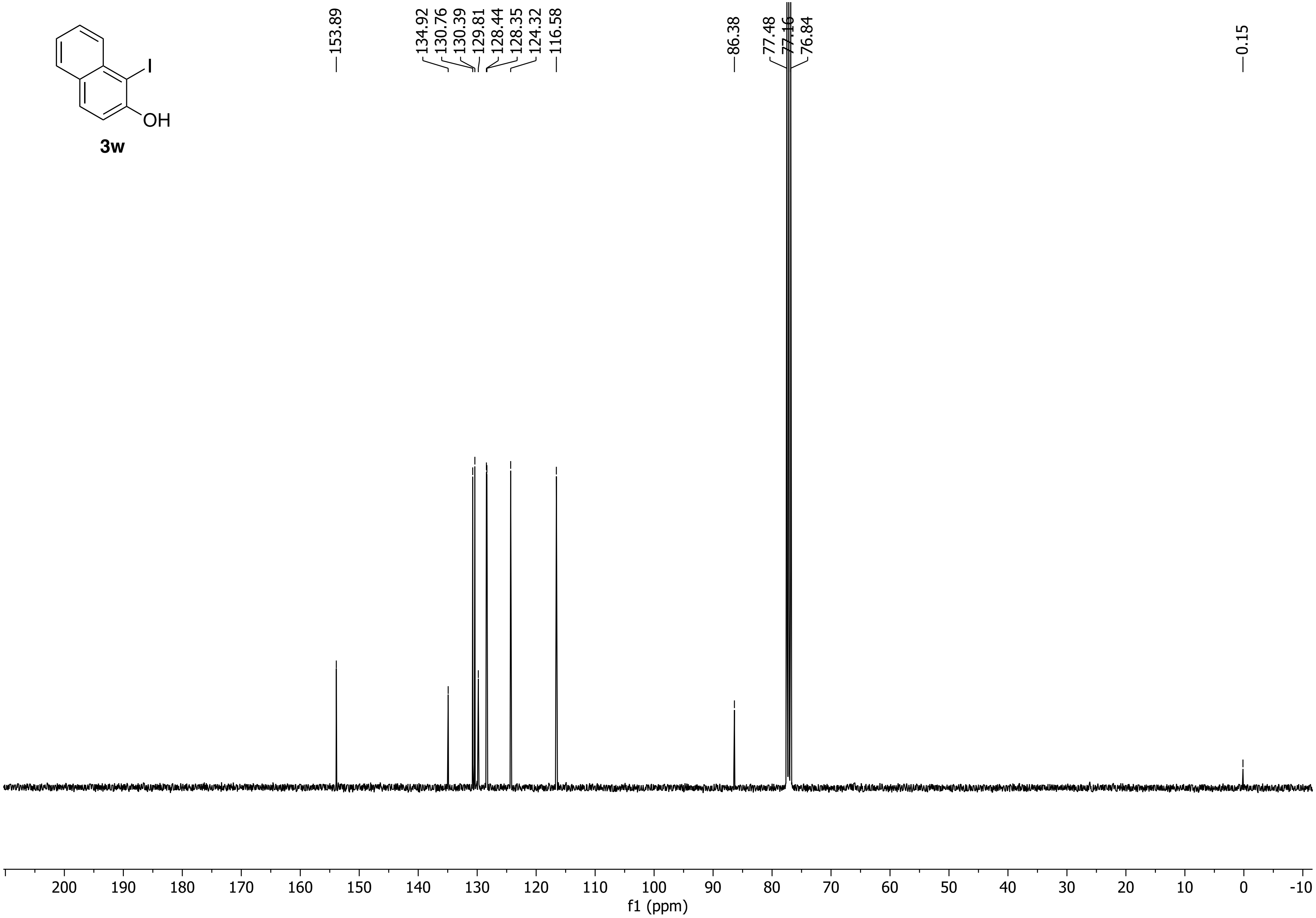

^{1}H NMR (400 MHz, $CDCl_3$)

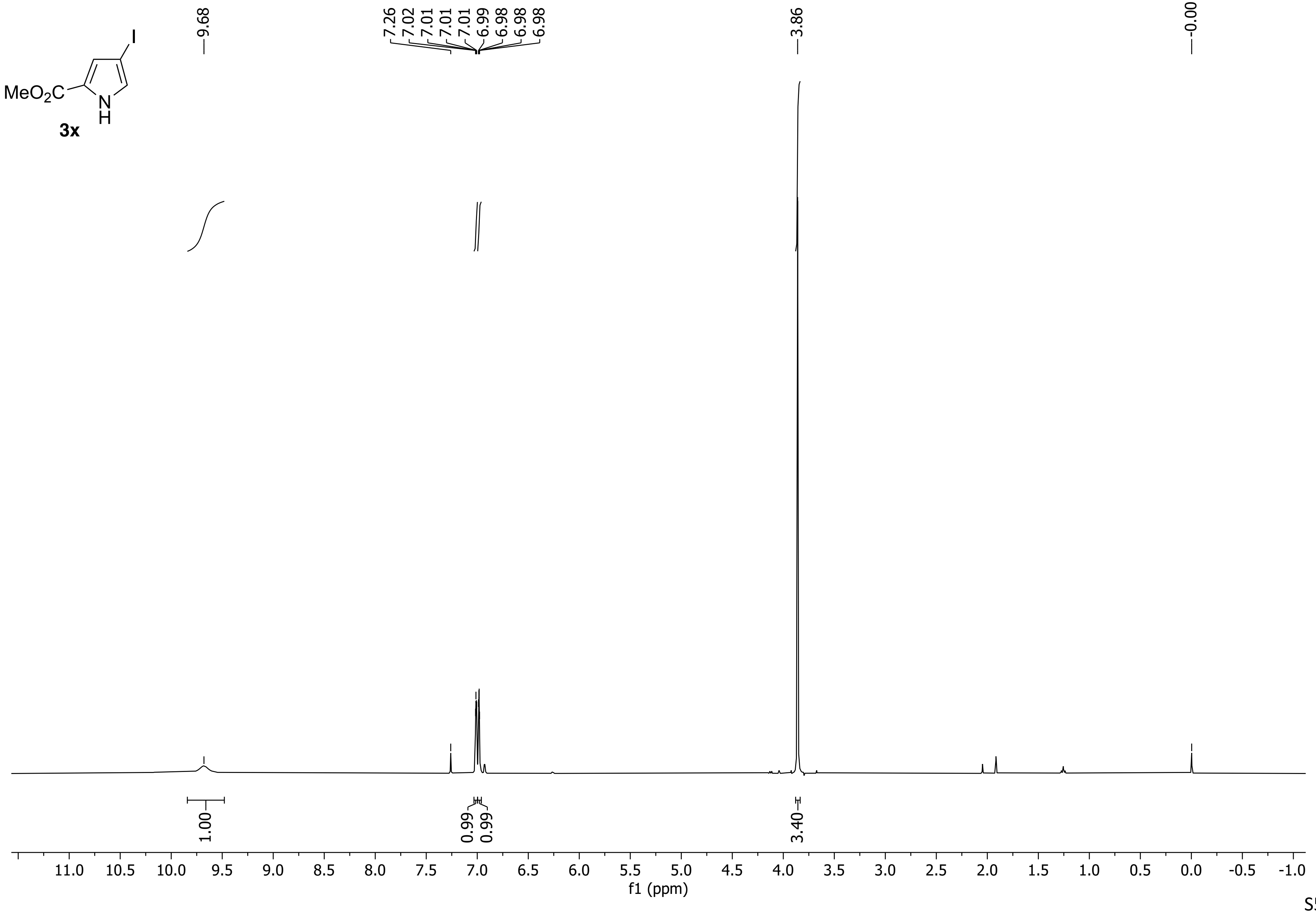

$^{13}C\{^{1}H\}$ NMR (101 MHz, $CDCl_3$)

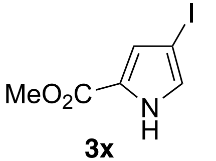

3x

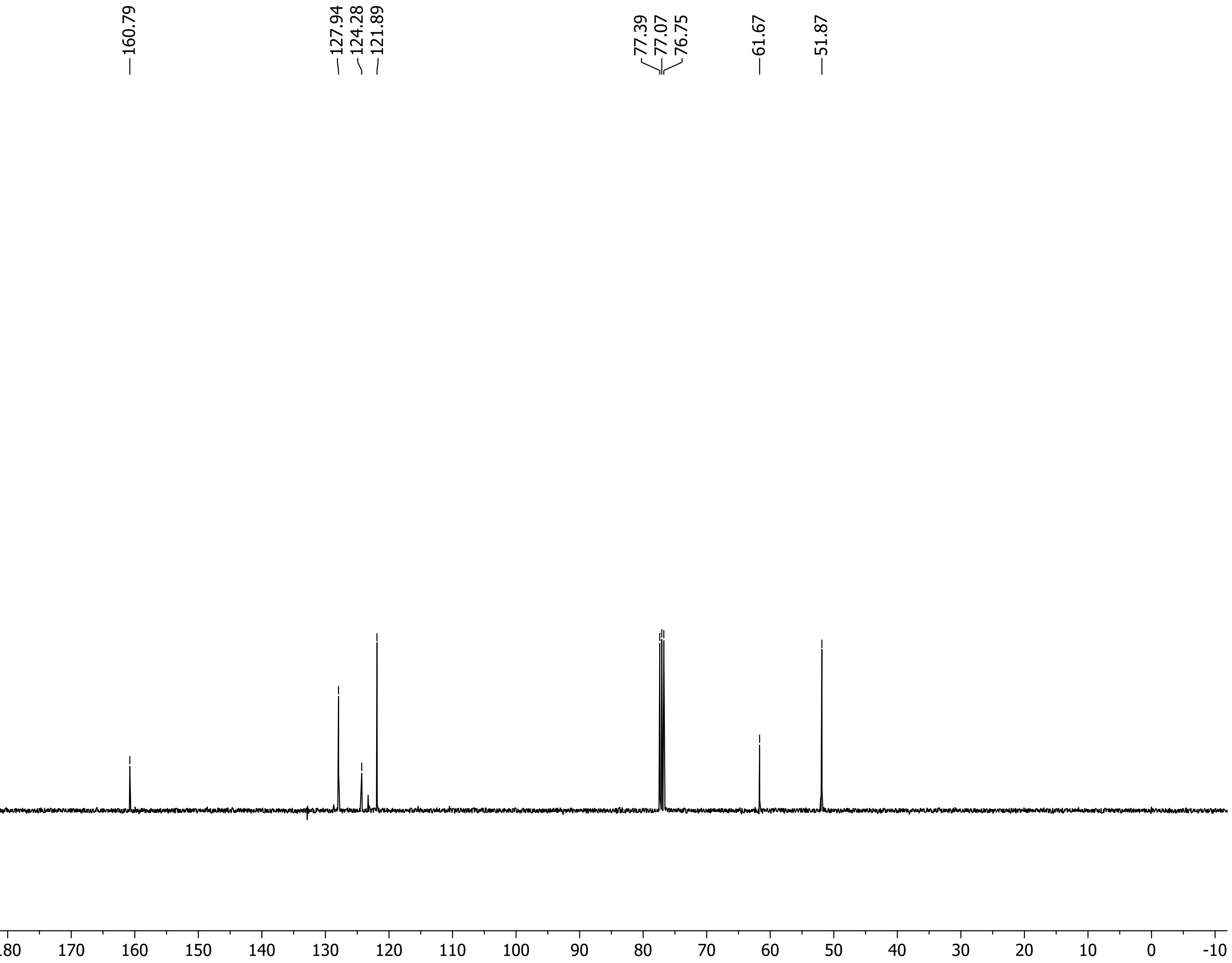

^{1}H NMR (400 MHz, $CDCl_3$)

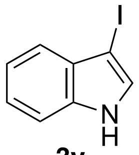

3y

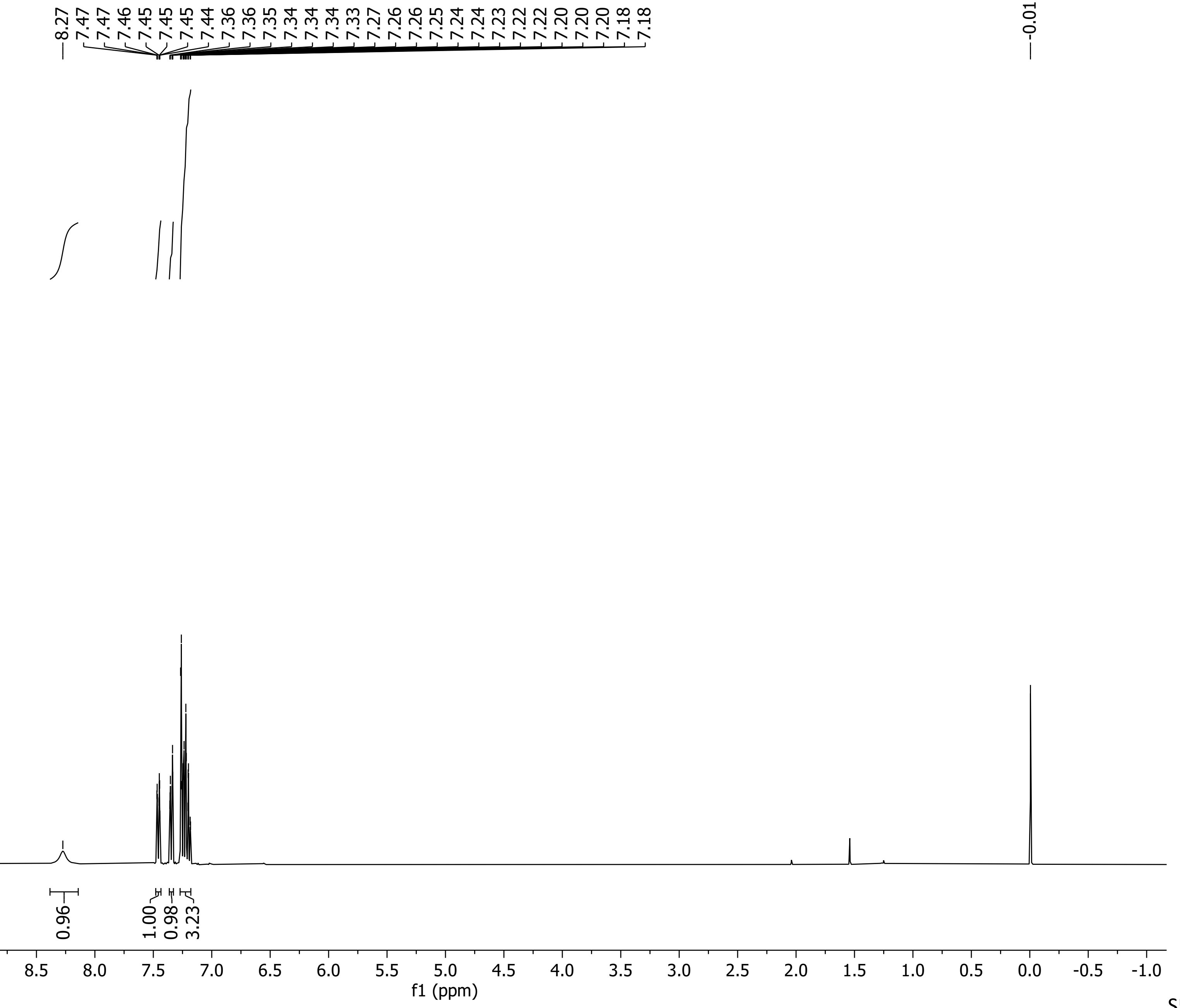

$^{13}C\{^1H\}$ NMR (101 MHz, $CDCl_3$)

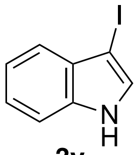

3y

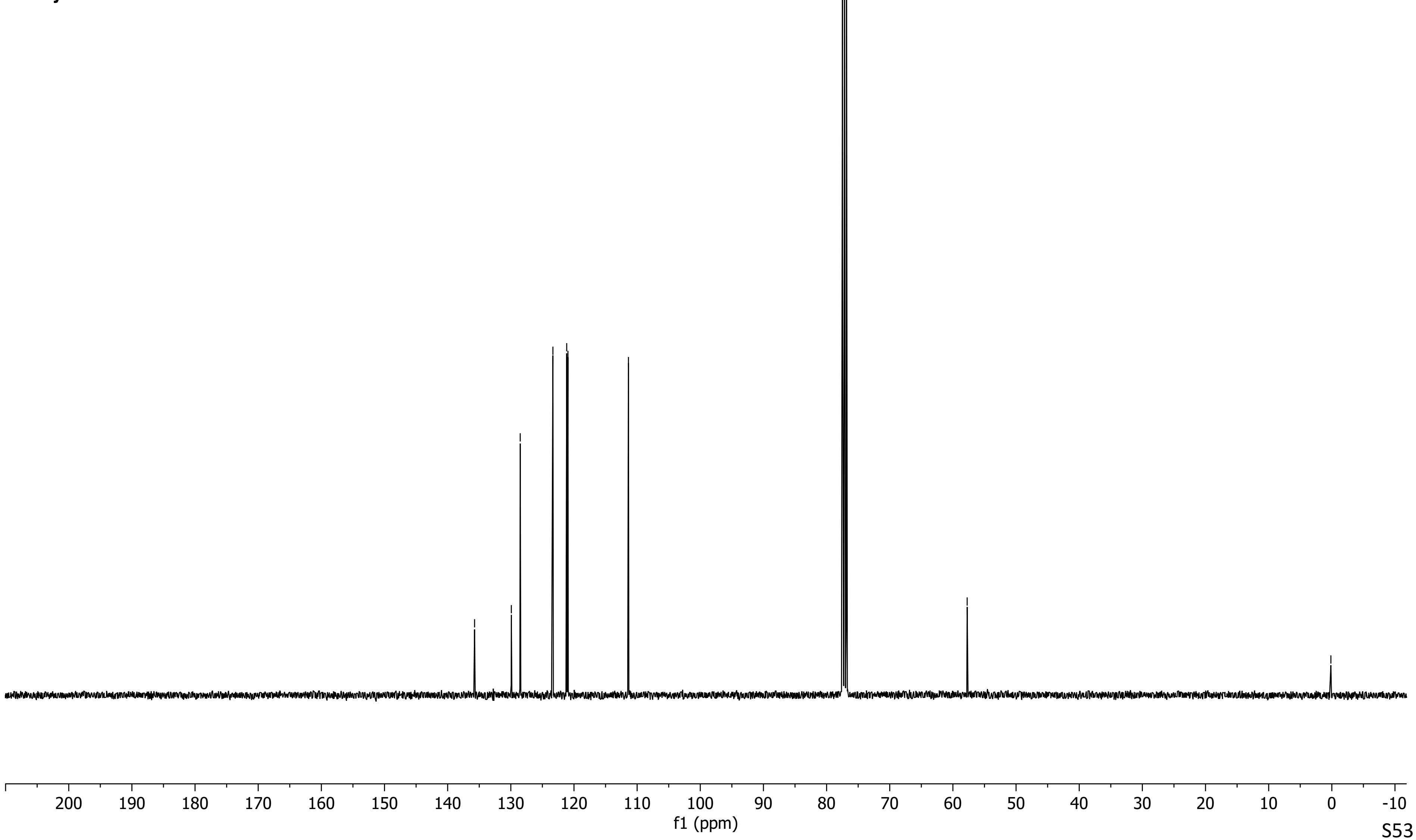

^{1}H NMR (400 MHz, $CDCl_3$)

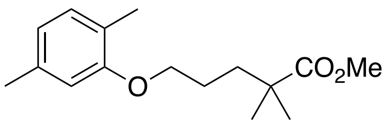

4a

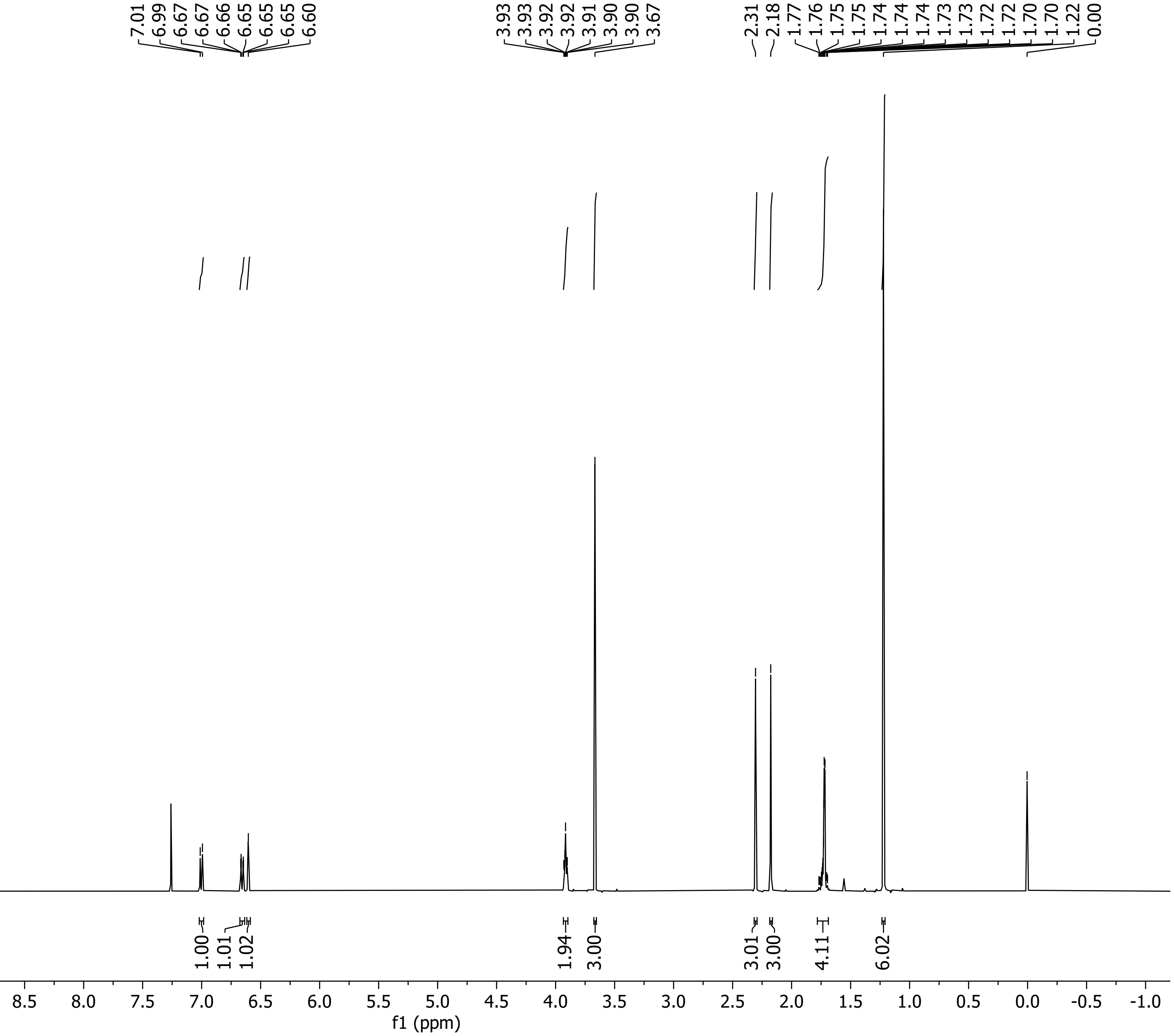

$^{13}C\{^{1}H\}$ NMR (101 MHz, $CDCl_3$)

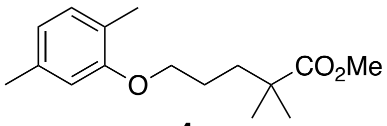

4a

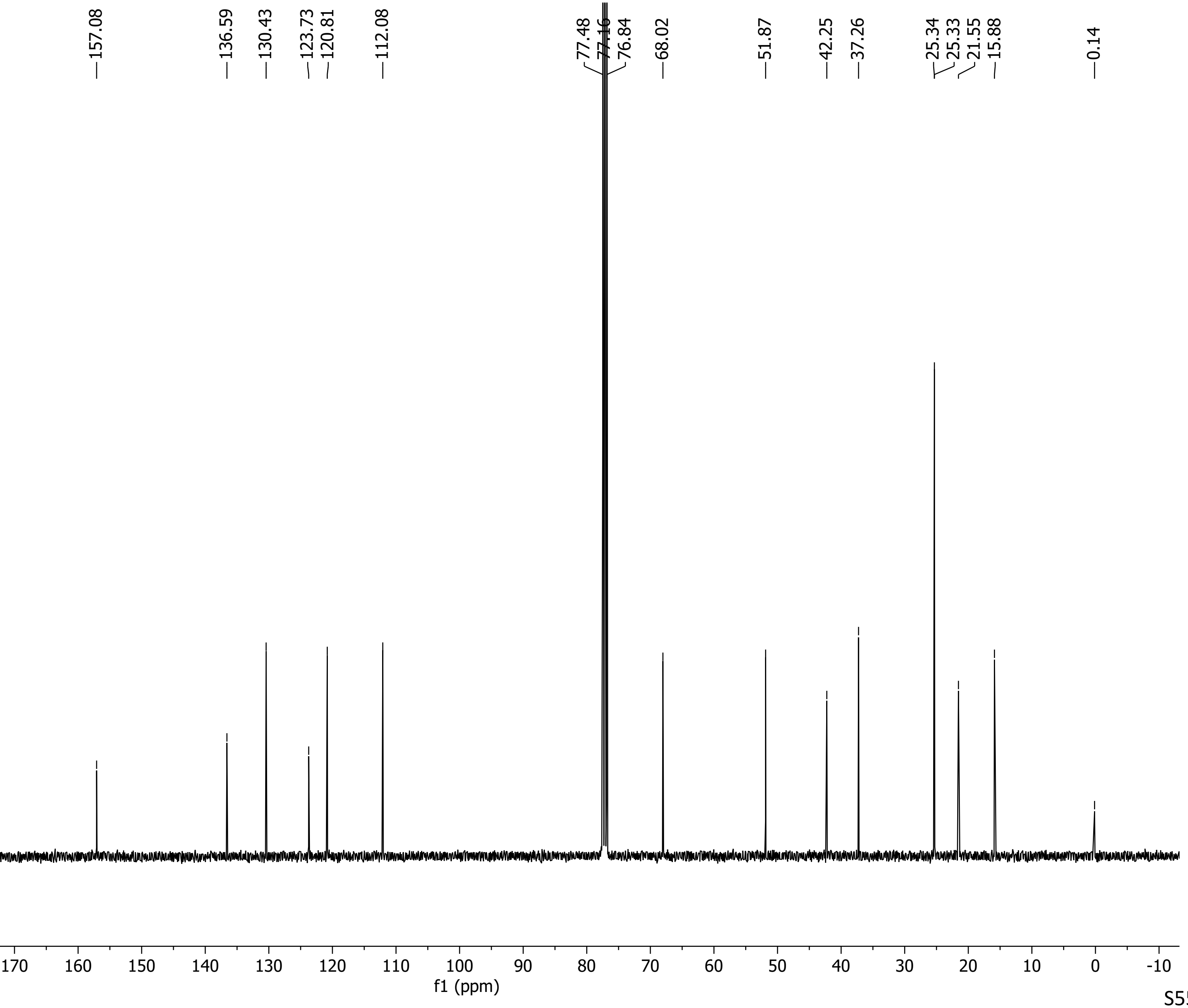

^{1}H NMR (400 MHz, CDCl$_3$)

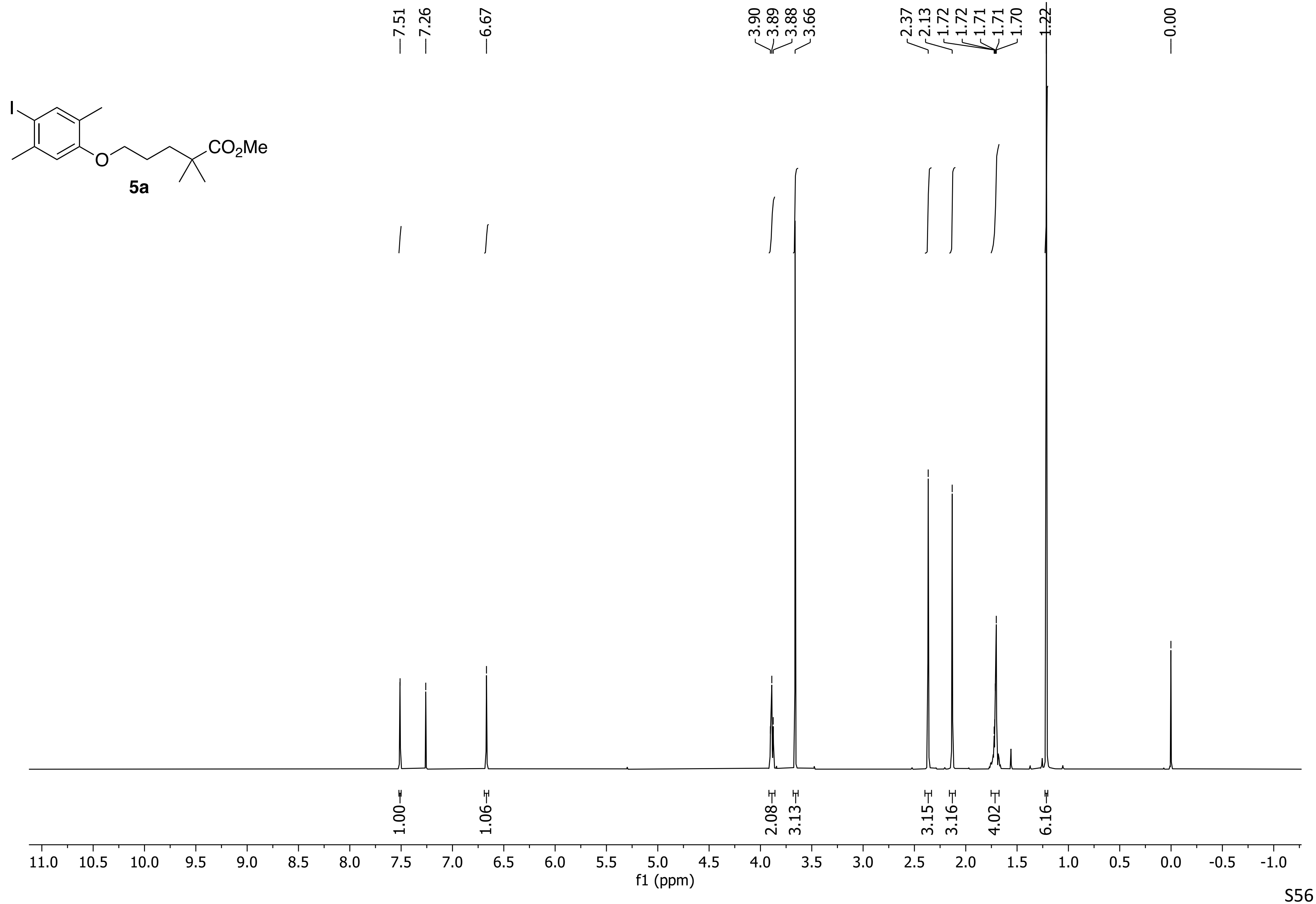

$^{13}C\{^{1}H\}$ NMR (101 MHz, $CDCl_3$)

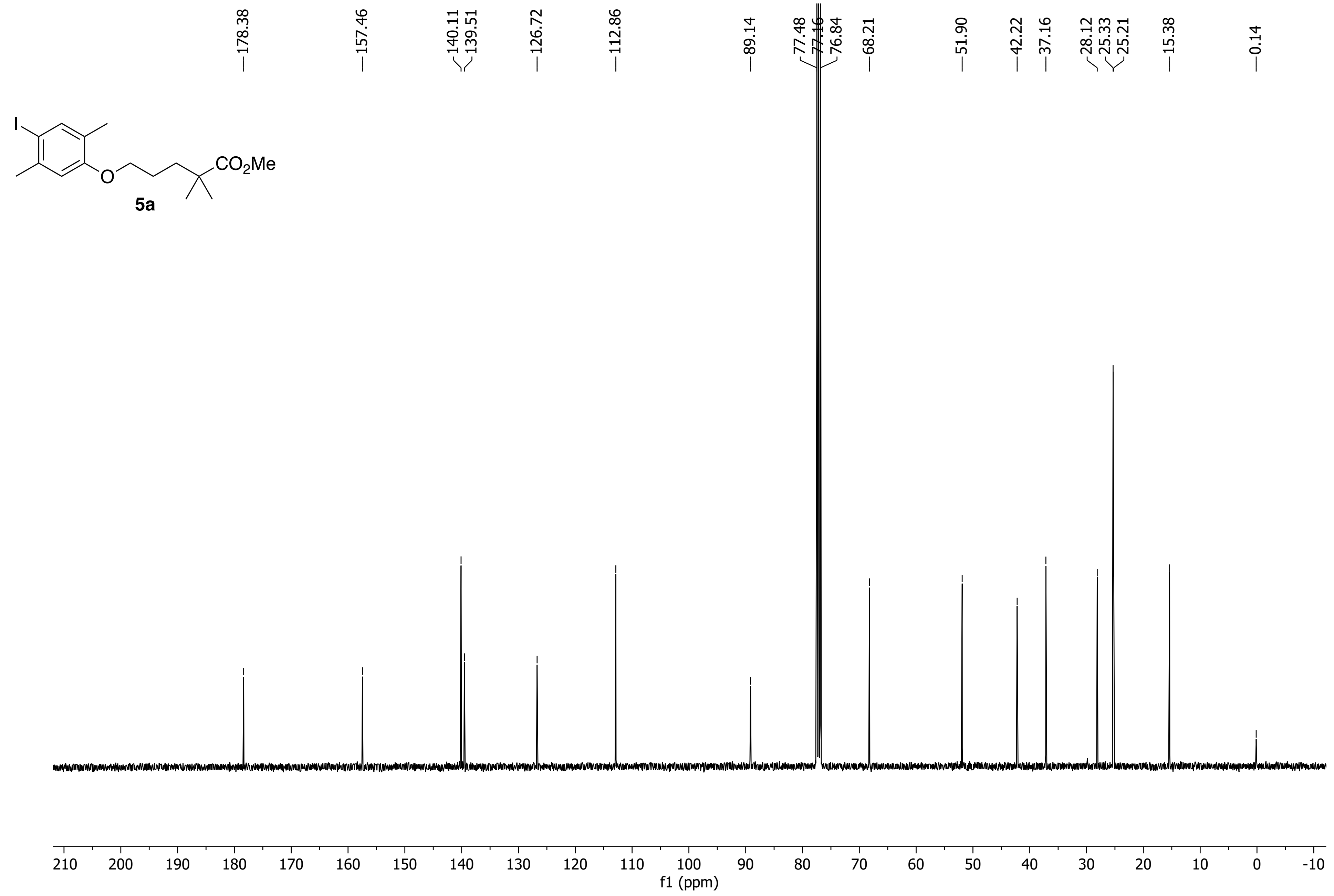

^{1}H NMR (400 MHz, $CDCl_3$)

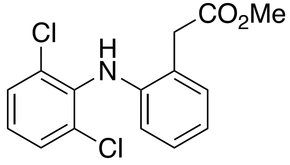

4b

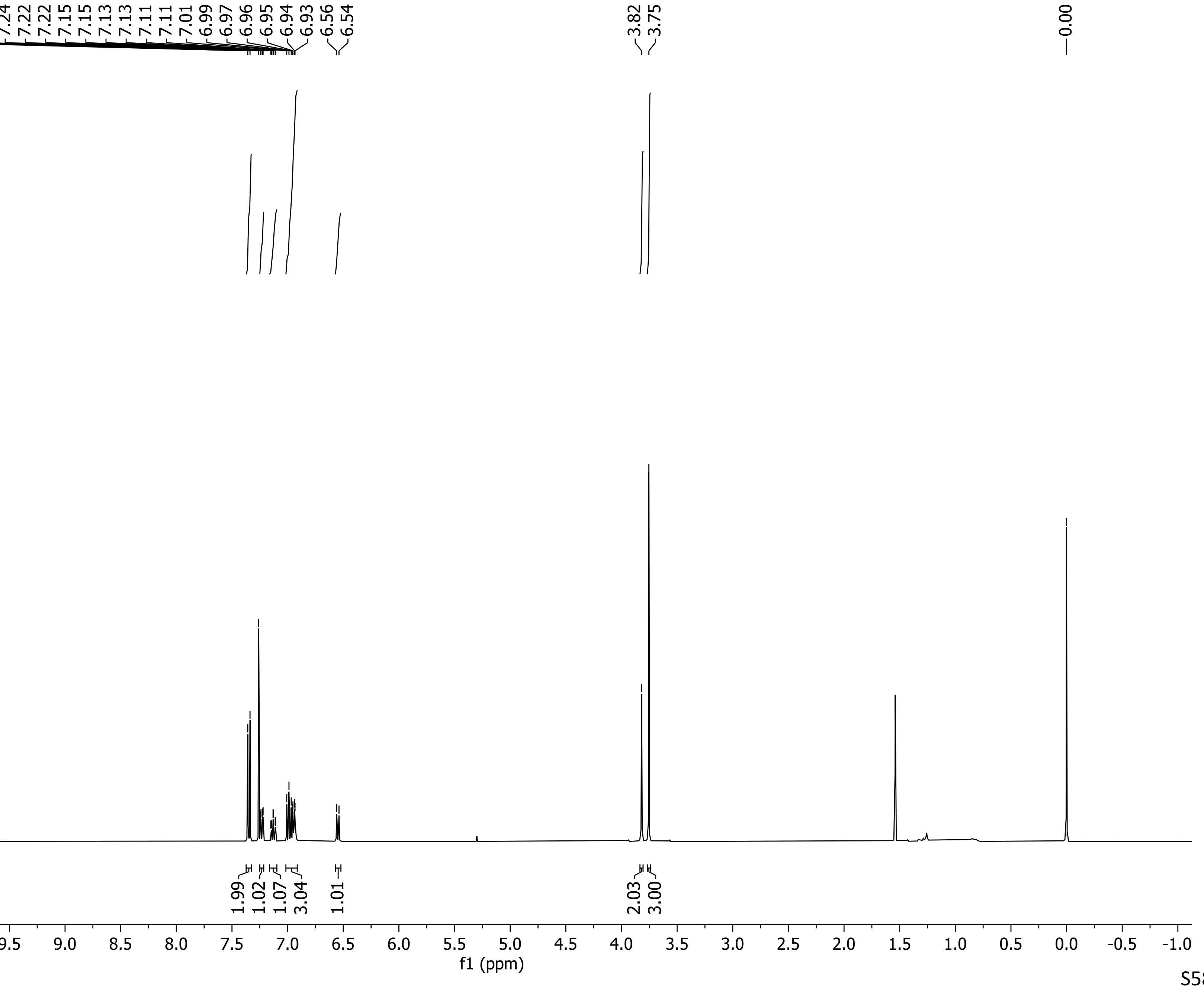

$^{13}C\{^{1}H\}$ NMR (101 MHz, $CDCl_3$)

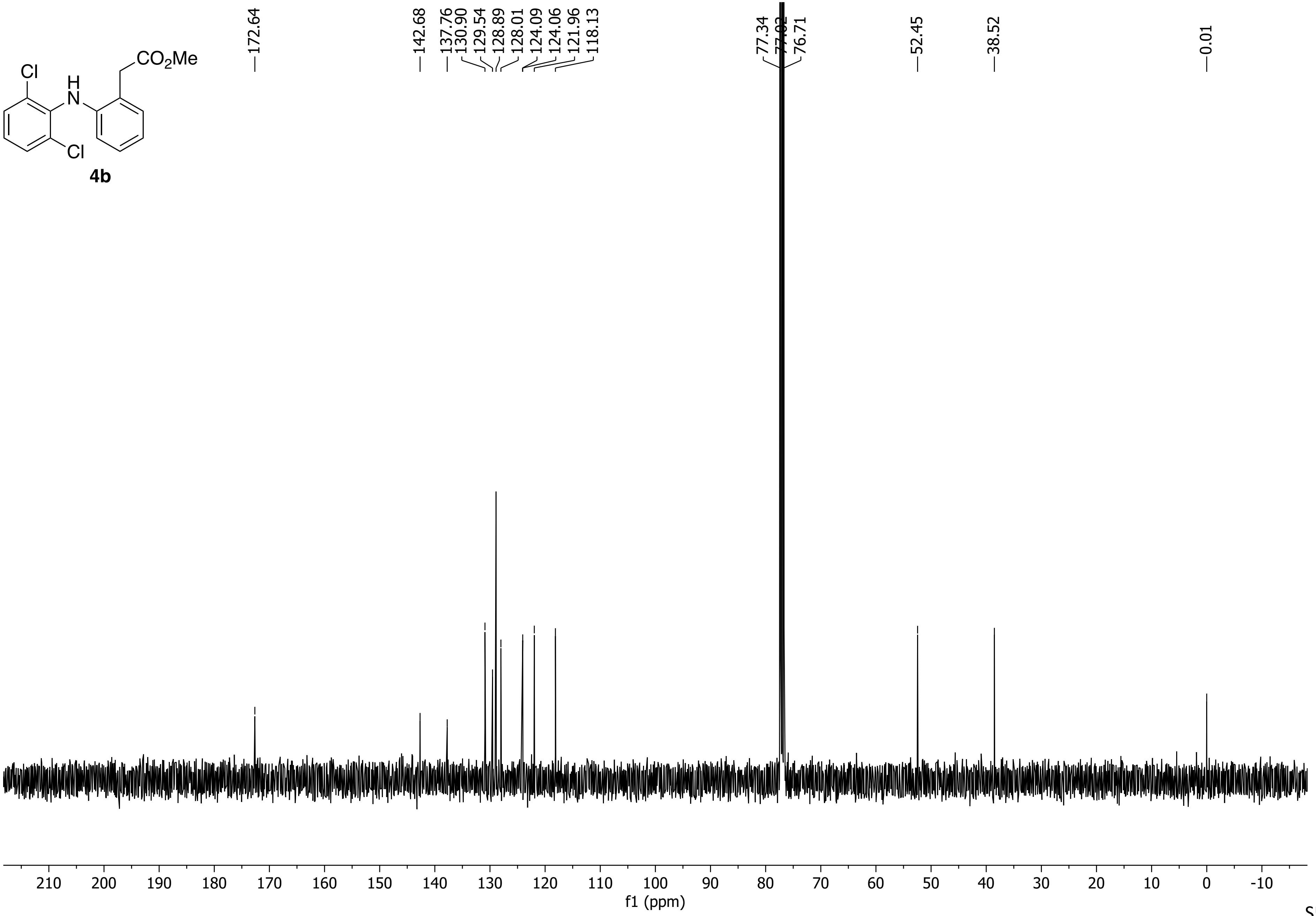

^{1}H NMR (400 MHz, CDCl$_3$)

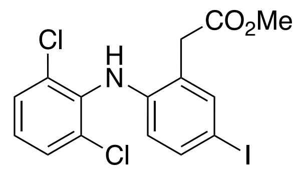

5b

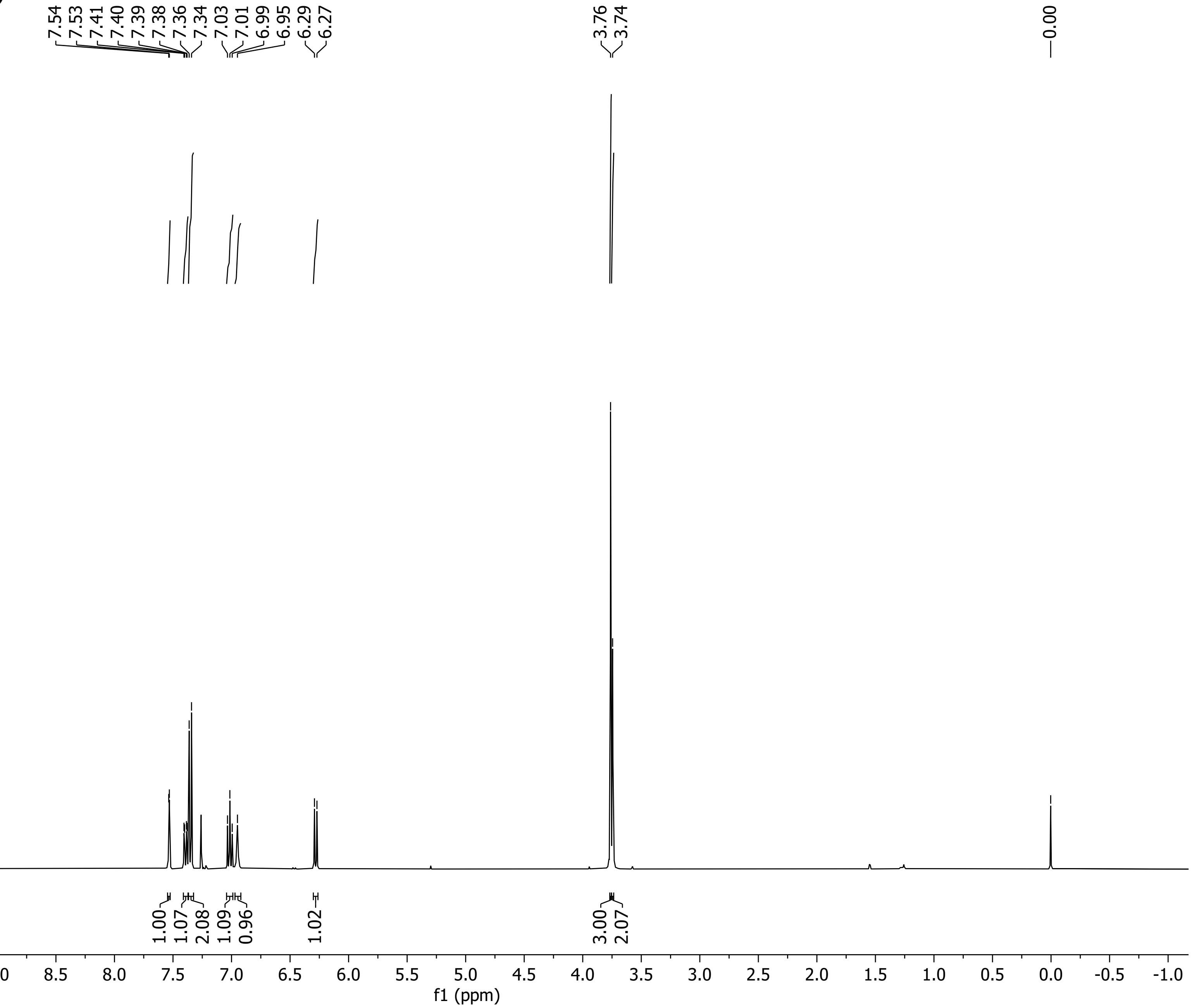

$^{13}C\{^{1}H\}$ NMR (101 MHz, $CDCl_3$)

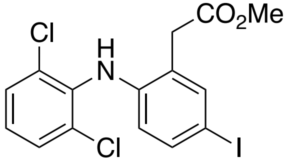

5b

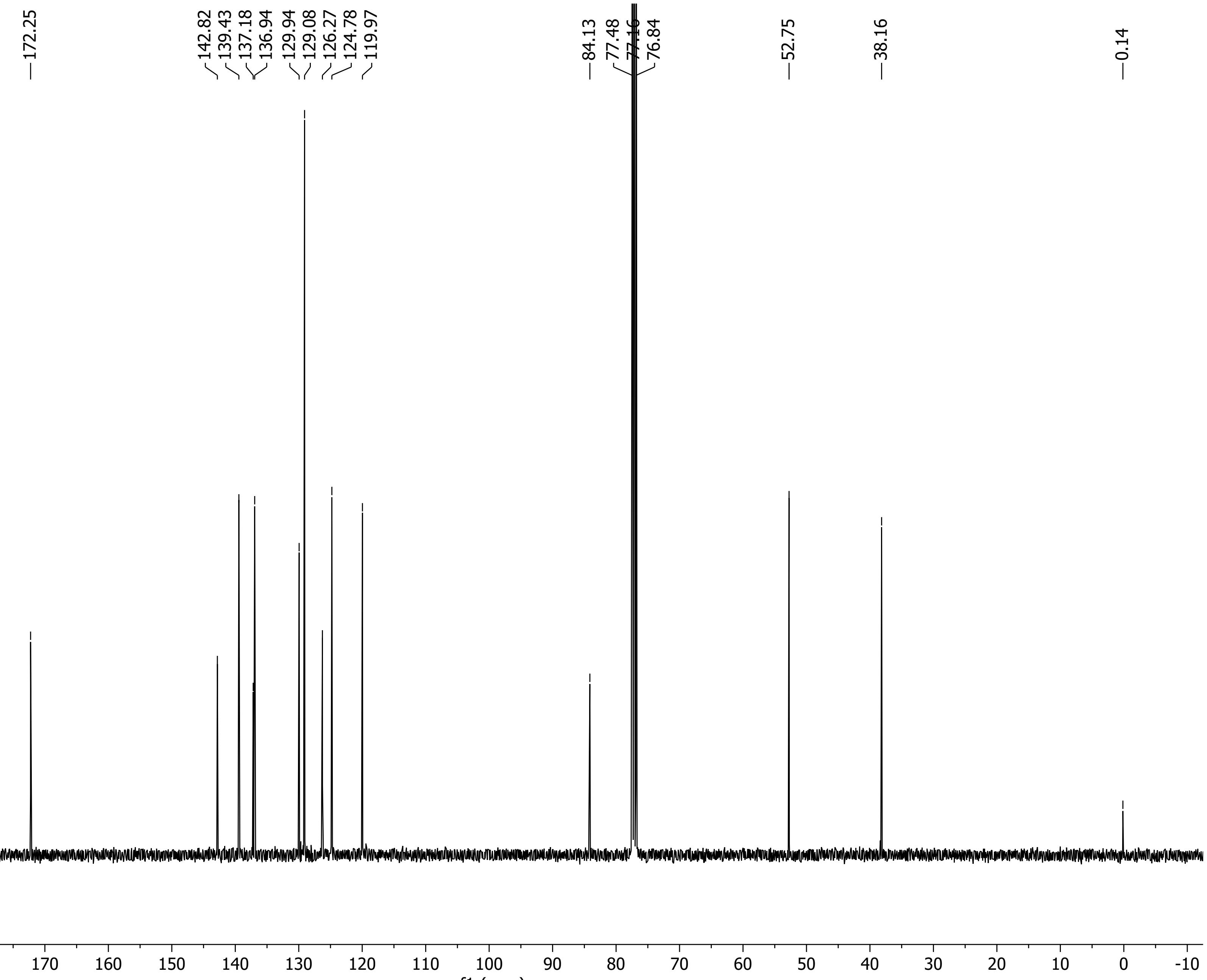

^{1}H NMR (400 MHz, CDCl$_3$)

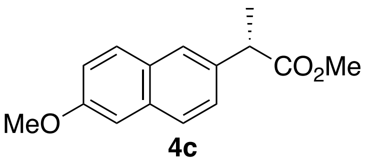

4c

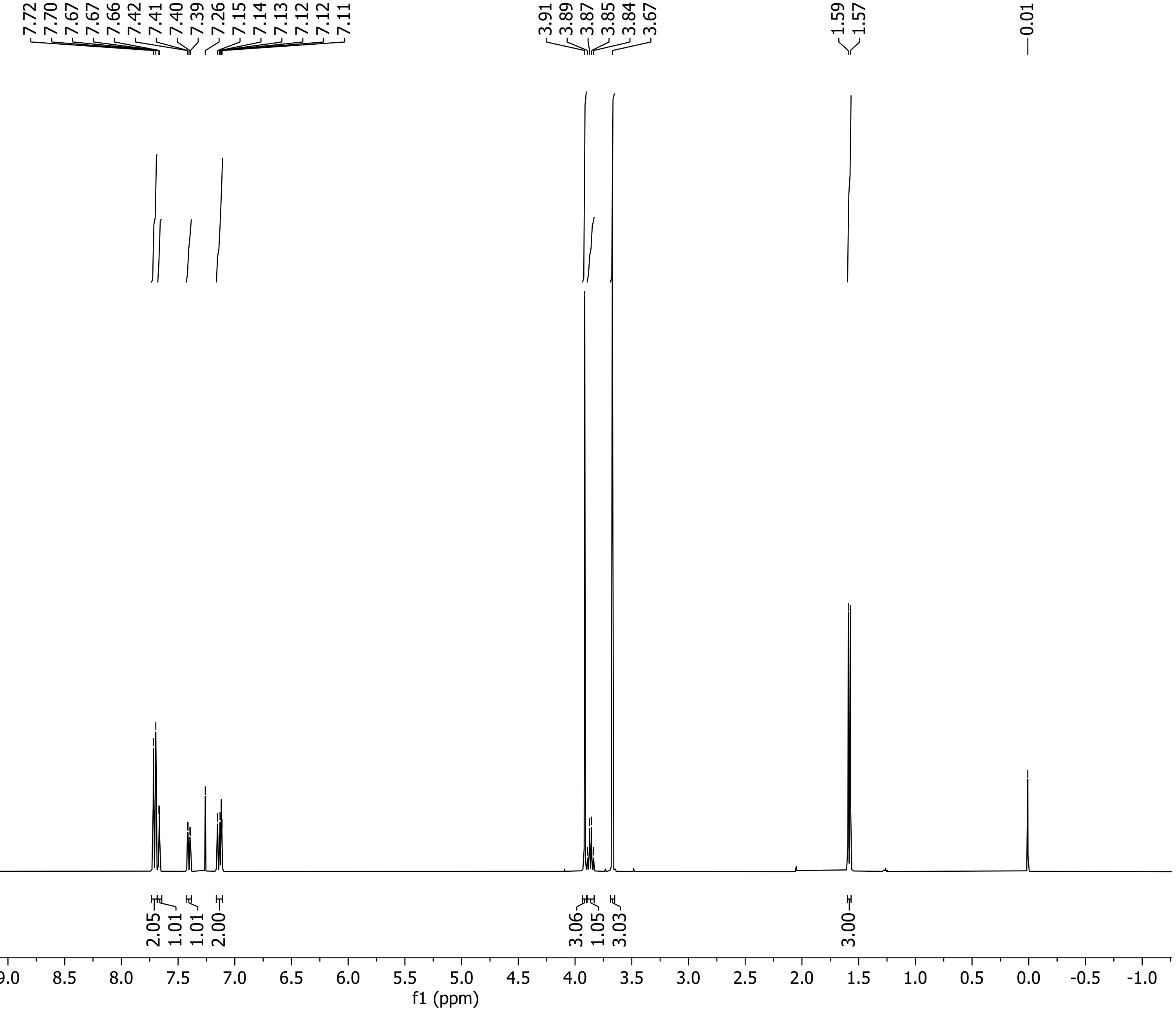

$^{13}C\{^1H\}$ NMR (101 MHz, $CDCl_3$)

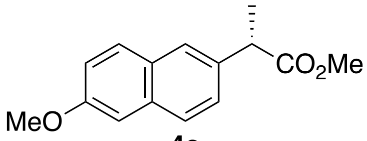

4c

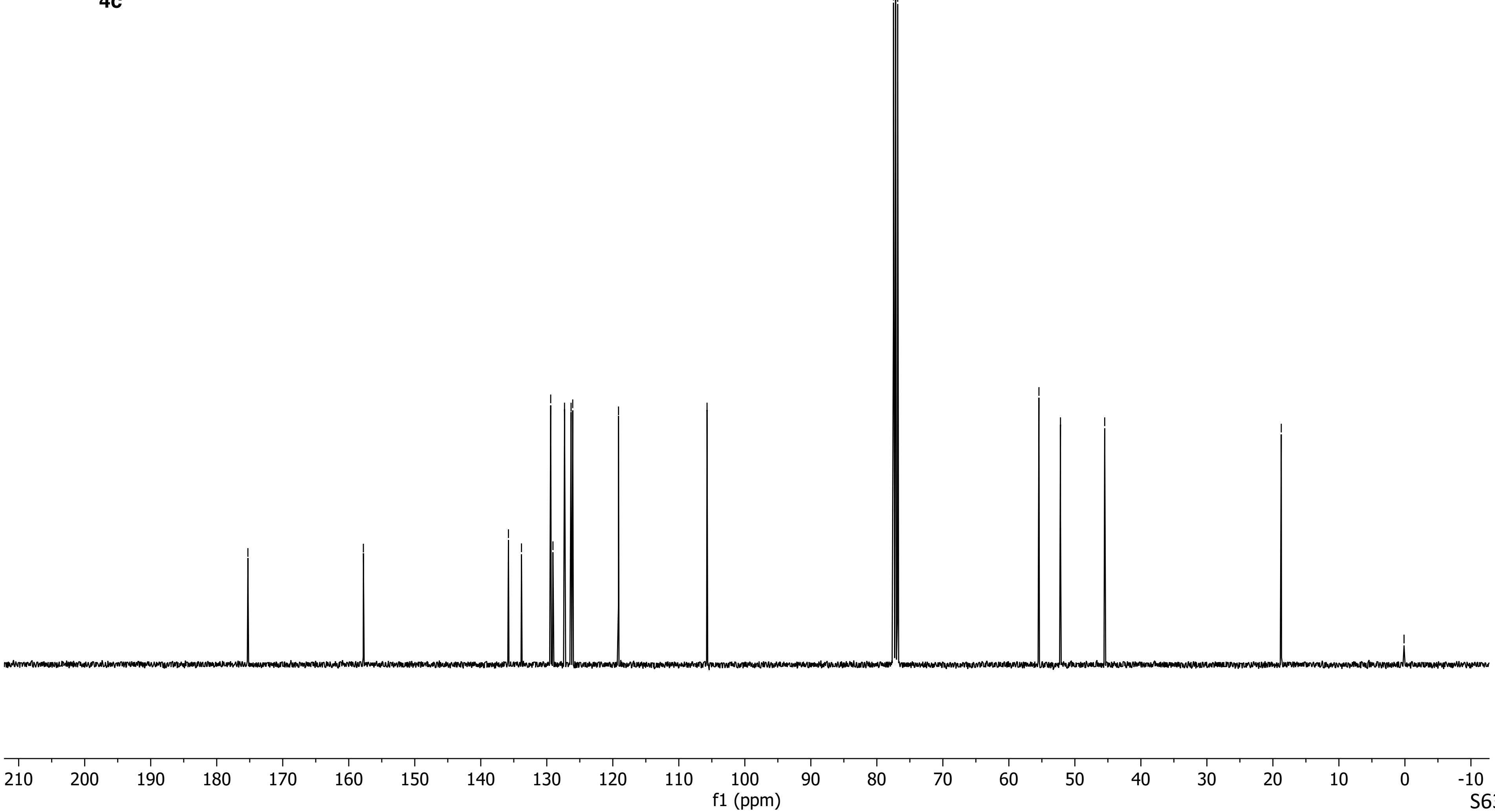

^{1}H NMR (400 MHz, $CDCl_3$)

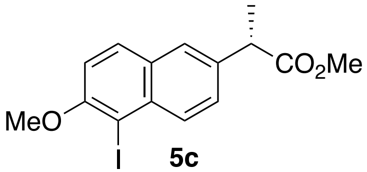

5c

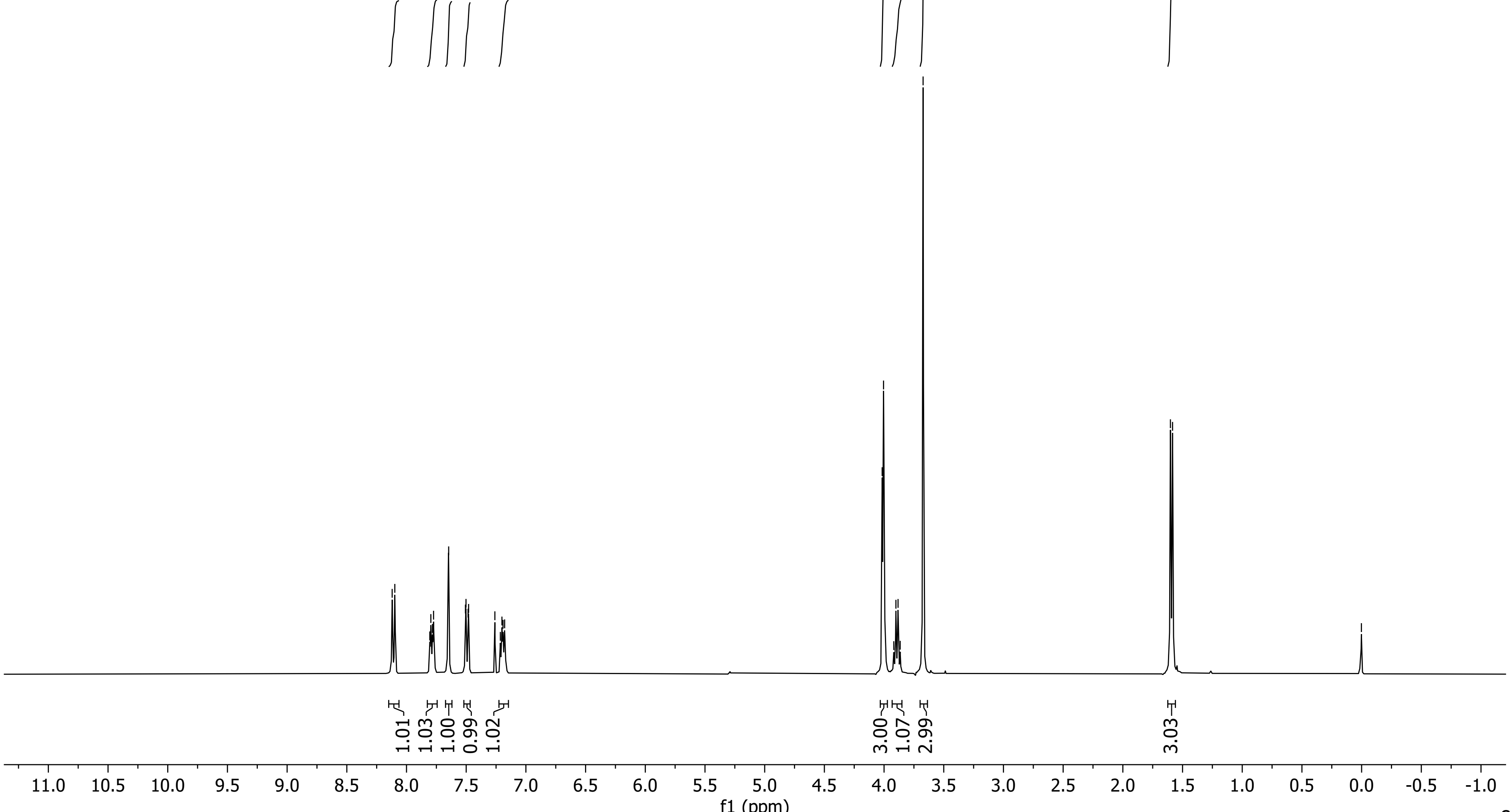

$^{13}C\{^{1}H\}$ NMR (101 MHz, $CDCl_3$)

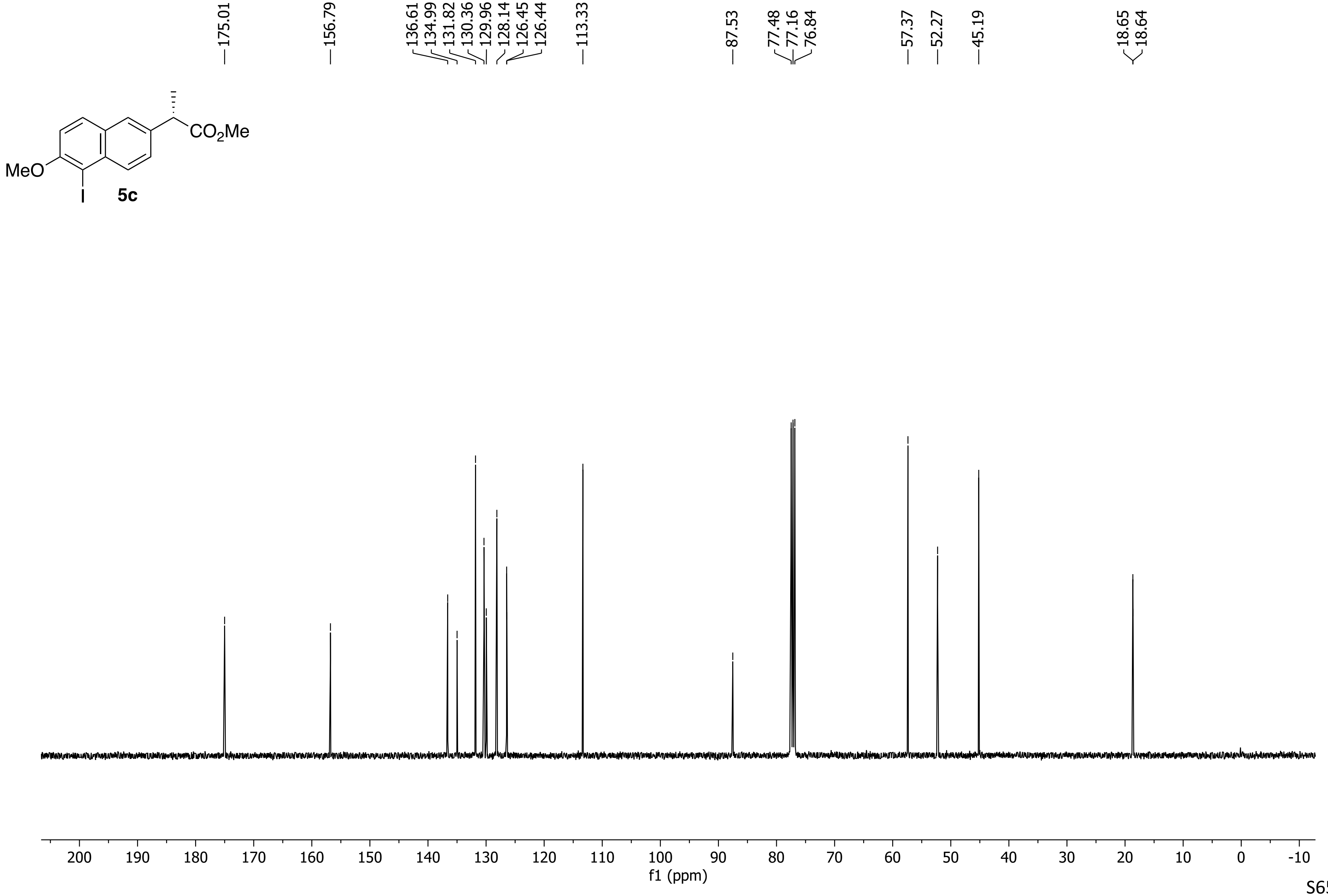

5c

^{1}H NMR (400 MHz, DMSO-d_6)

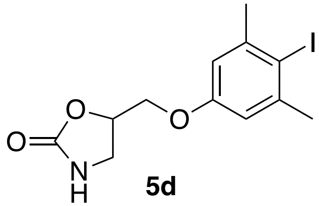

5d

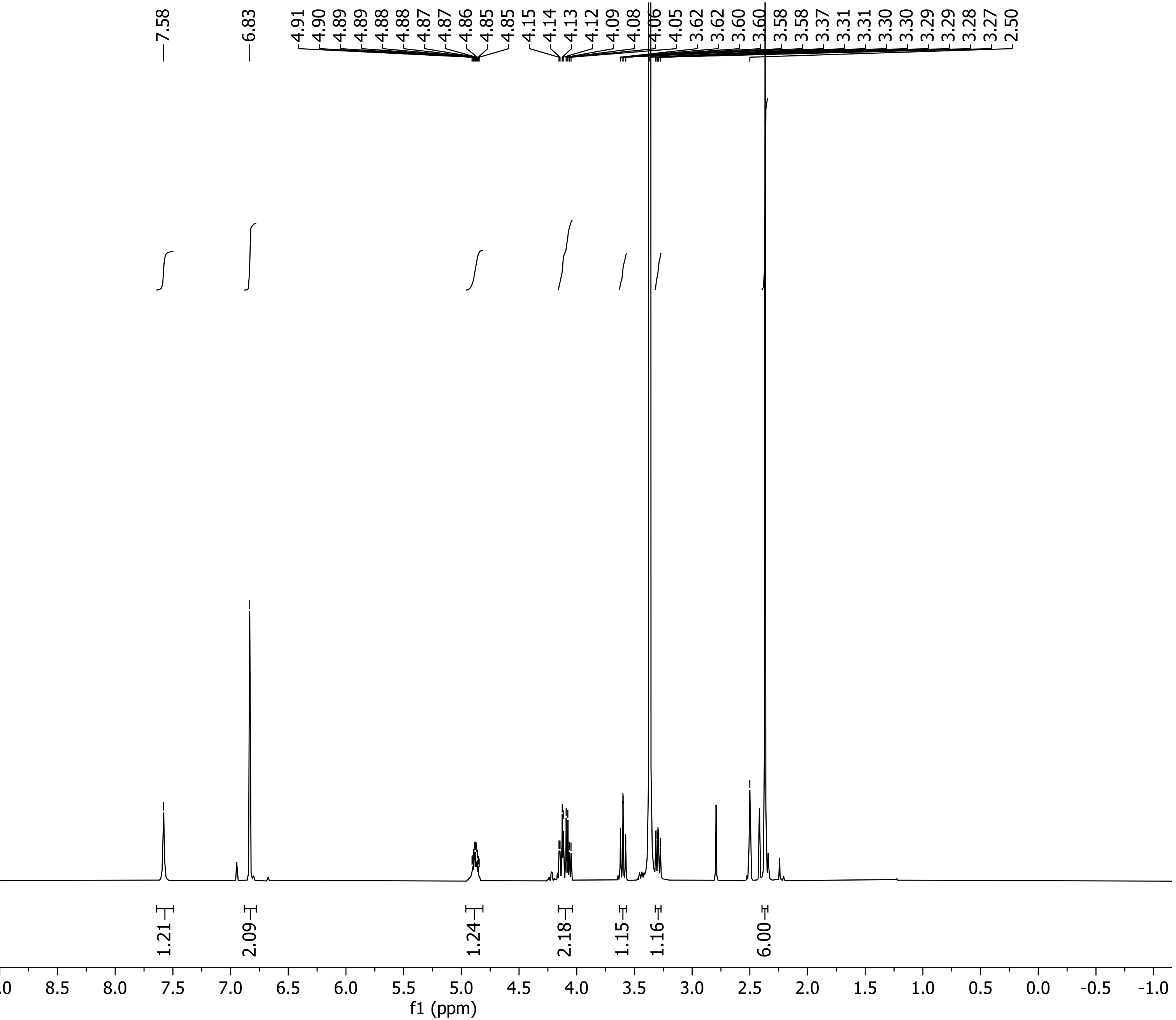

$^{13}C\{^{1}H\}$ NMR (101 MHz, DMSO-d_6)

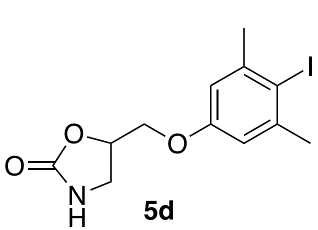

5d

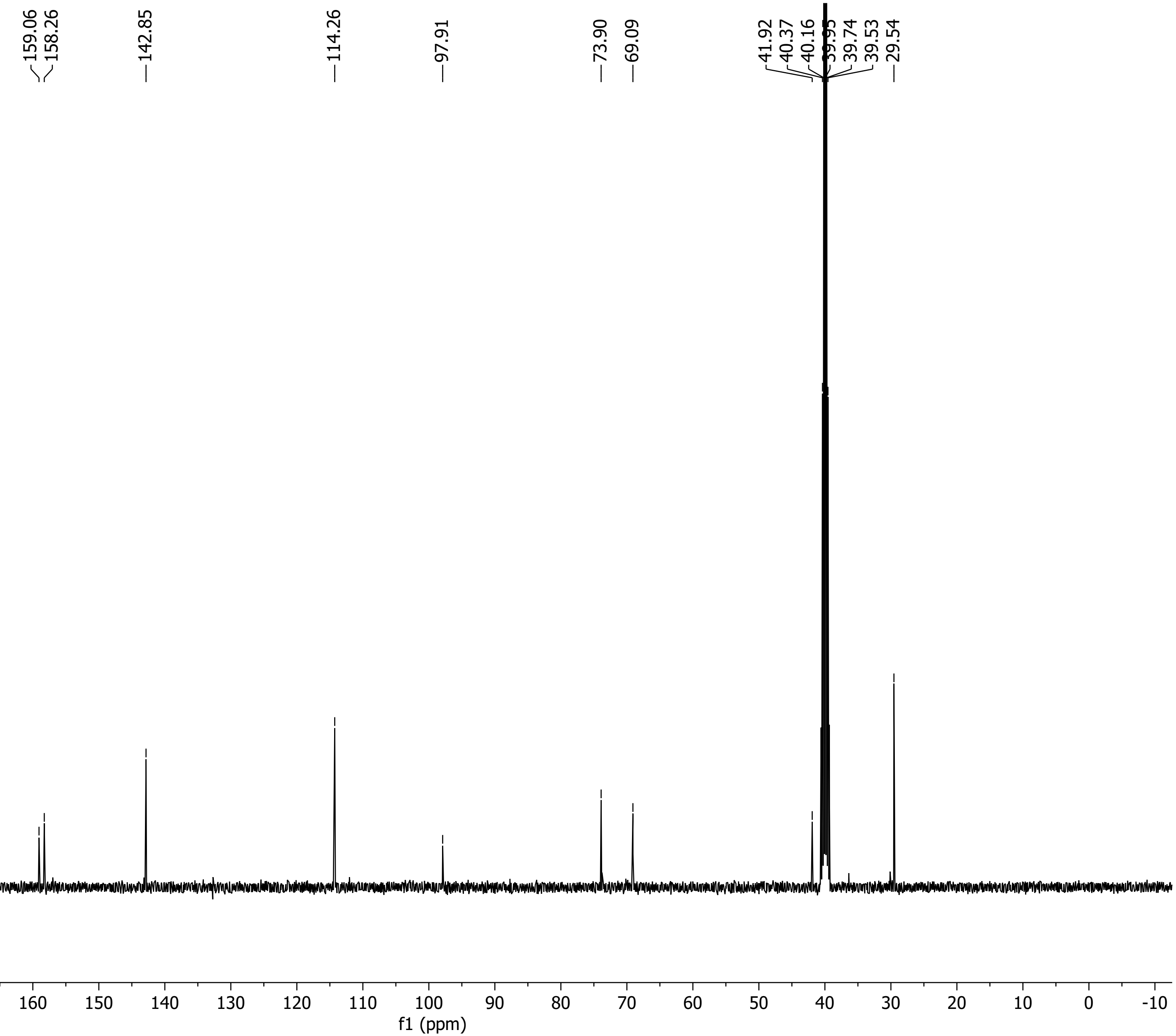

^{1}H NMR (400 MHz, $CDCl_3$)

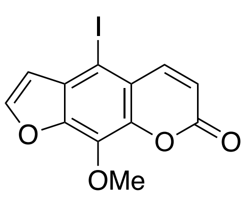

5e

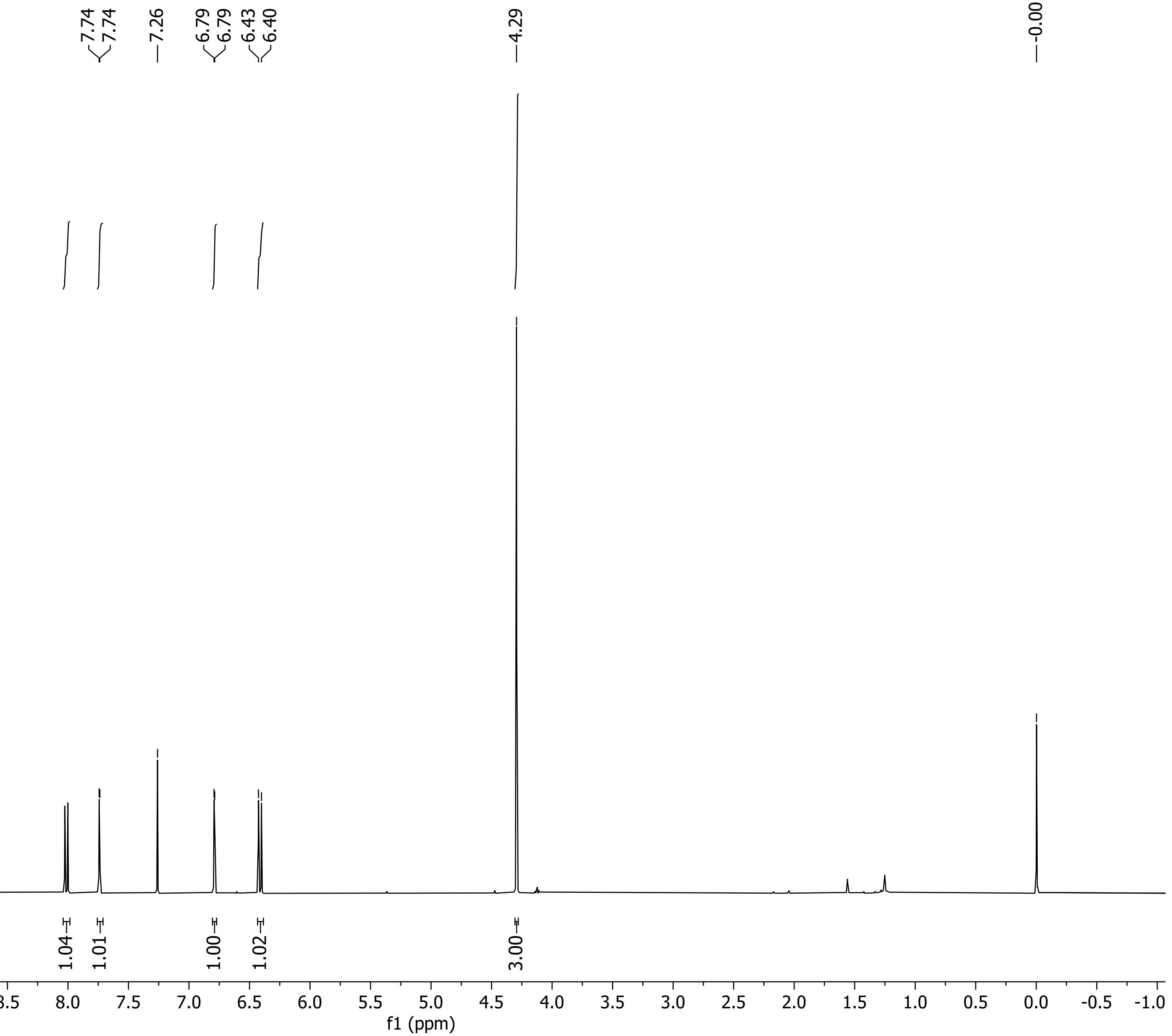

$^{13}C\{^1H\}$ NMR (101 MHz, $CDCl_3$)

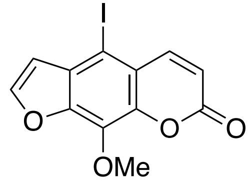

5e

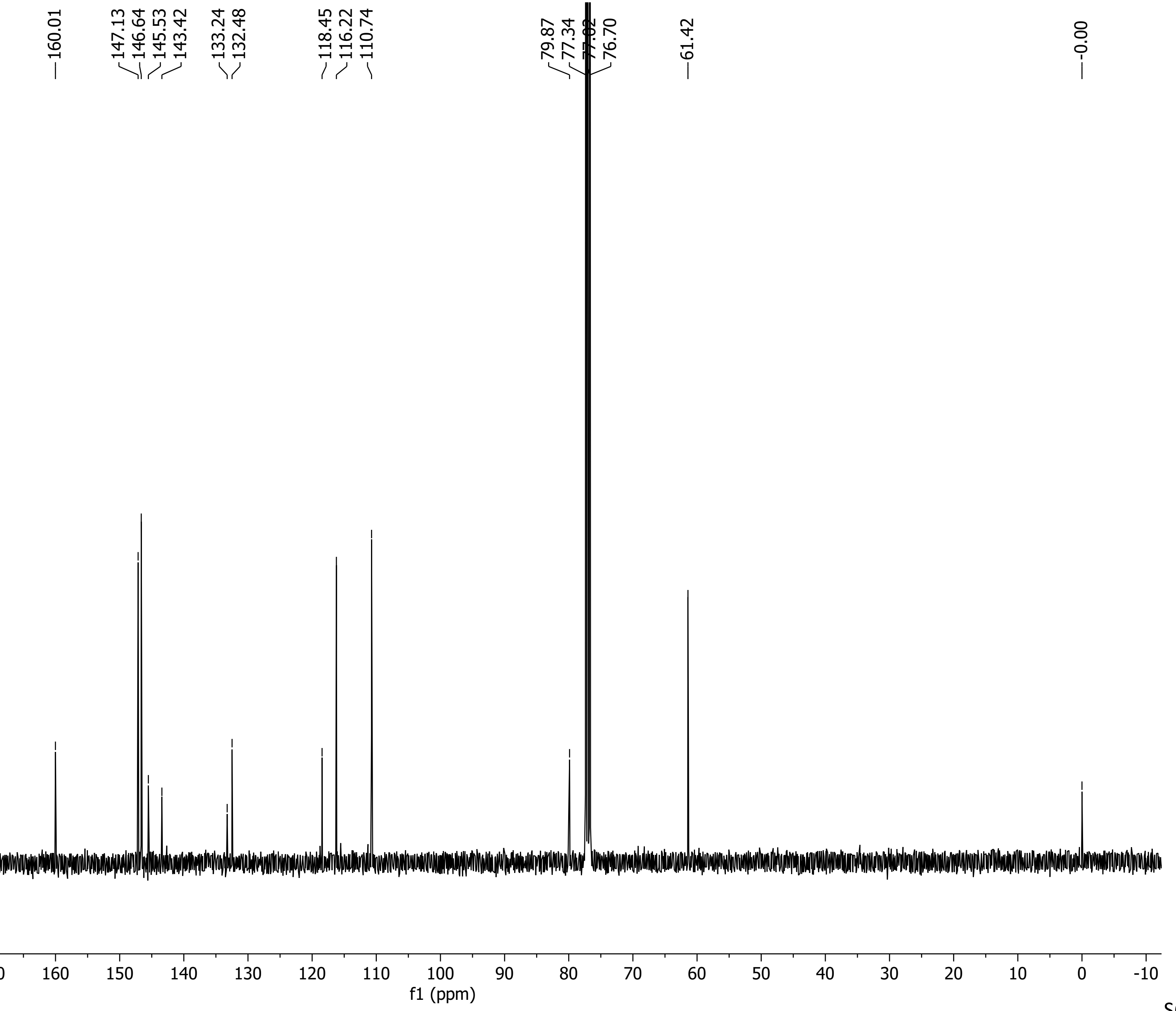

^{1}H NMR (400 MHz, $CDCl_3$)

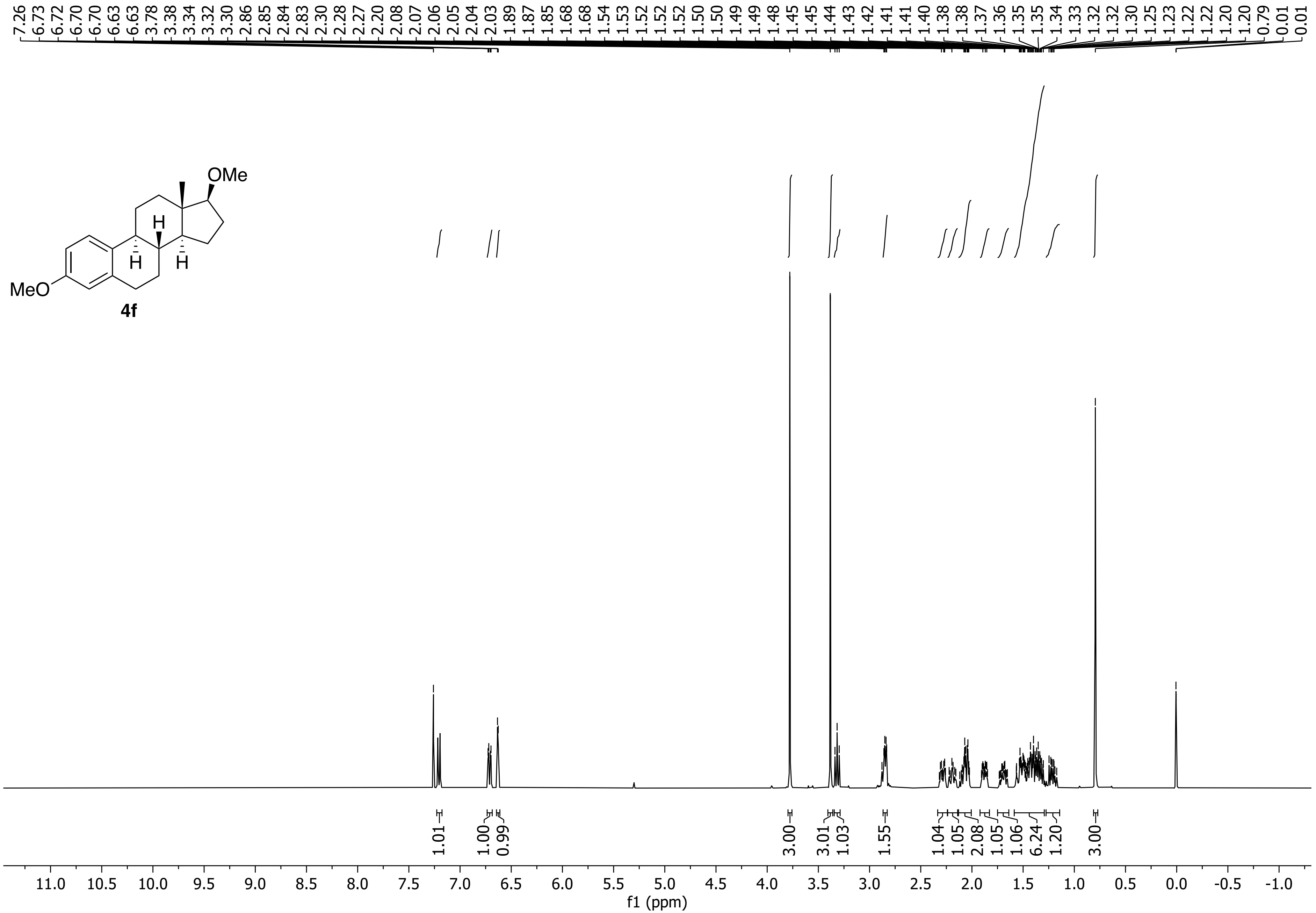

4f

$^{13}C\{^1H\}$ NMR (101 MHz, $CDCl_3$)

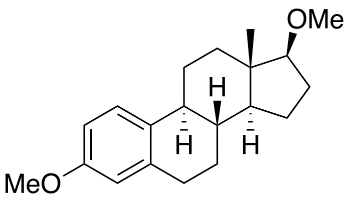

4f

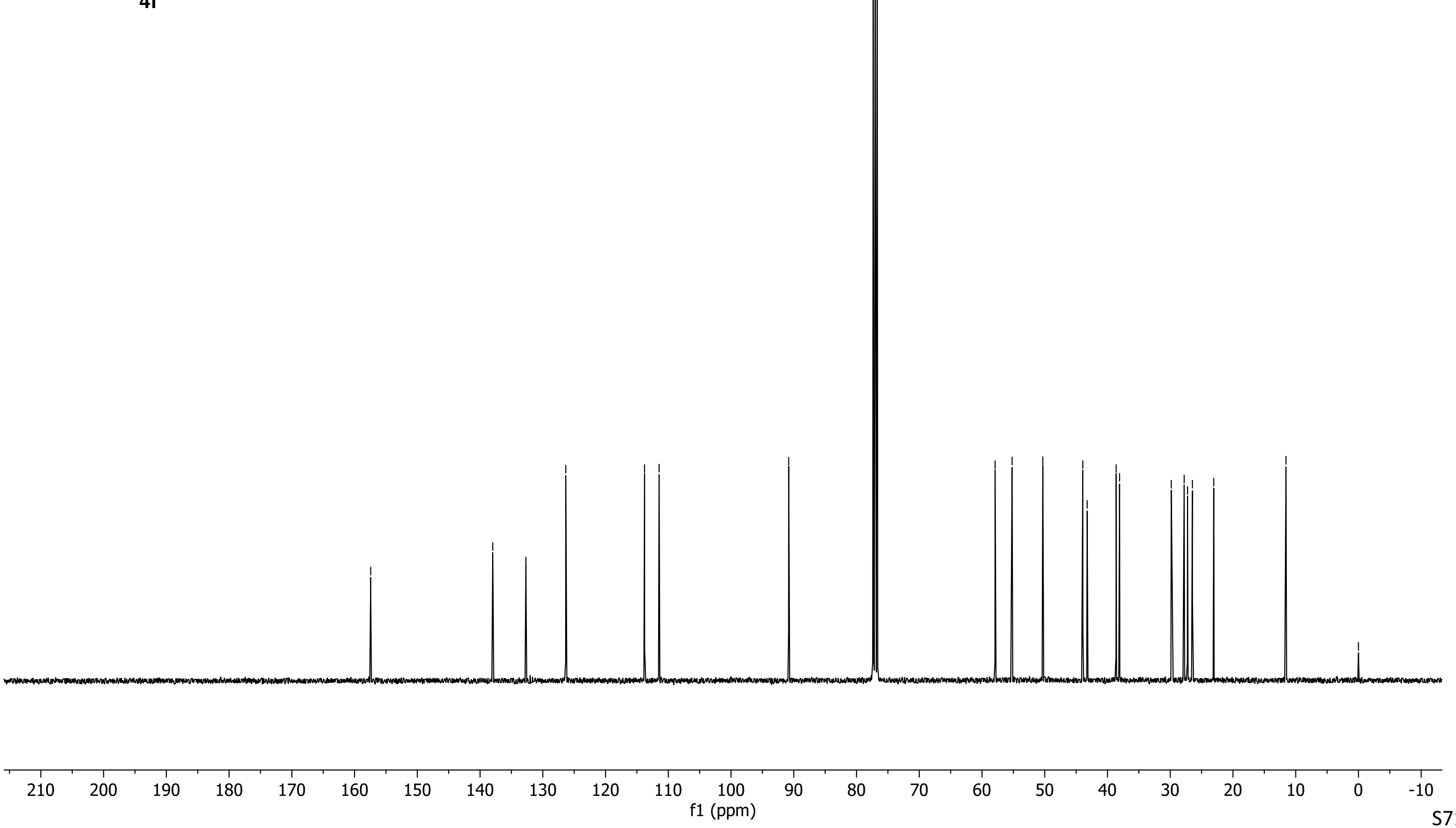

¹H NMR (400 MHz, $CDCl_3$)

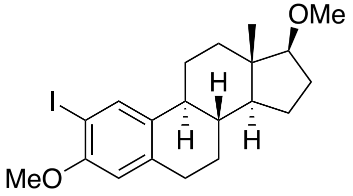

5f

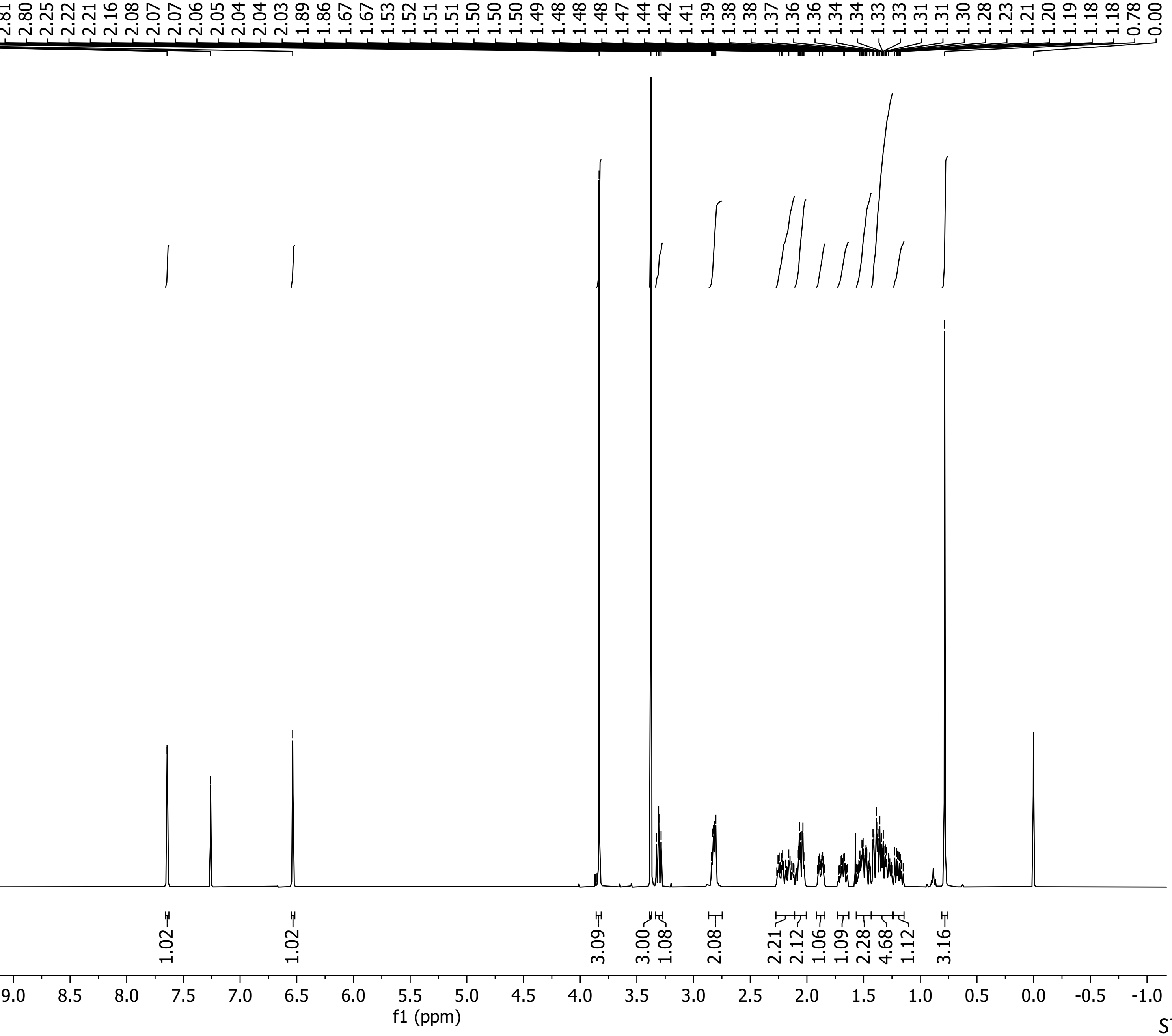

$^{13}C\{^{1}H\}$ NMR (101 MHz, $CDCl_3$)

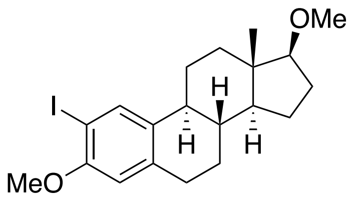

5f

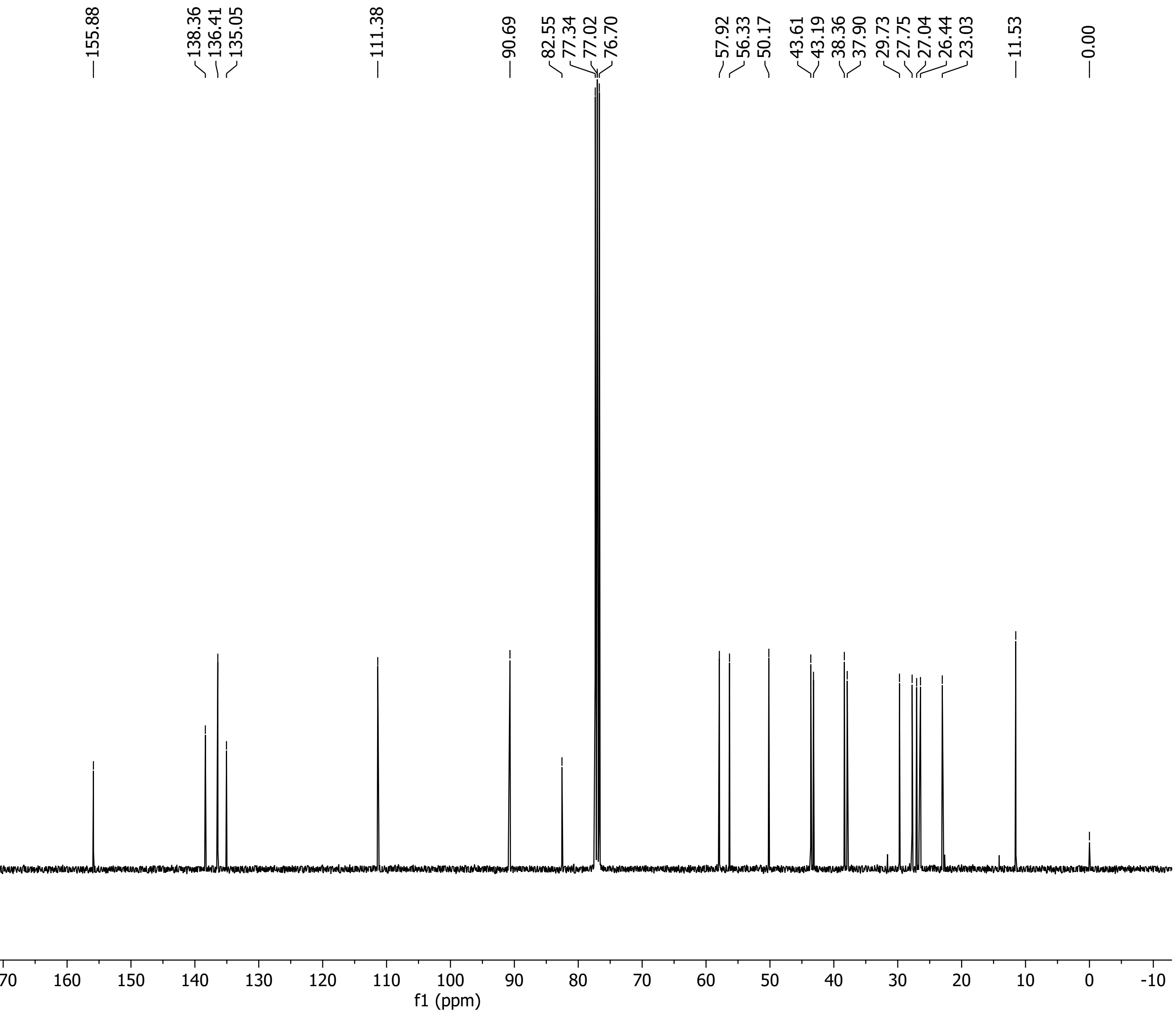

^{1}H NMR (400 MHz, $CDCl_3$)

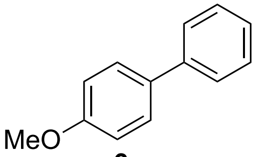

6a

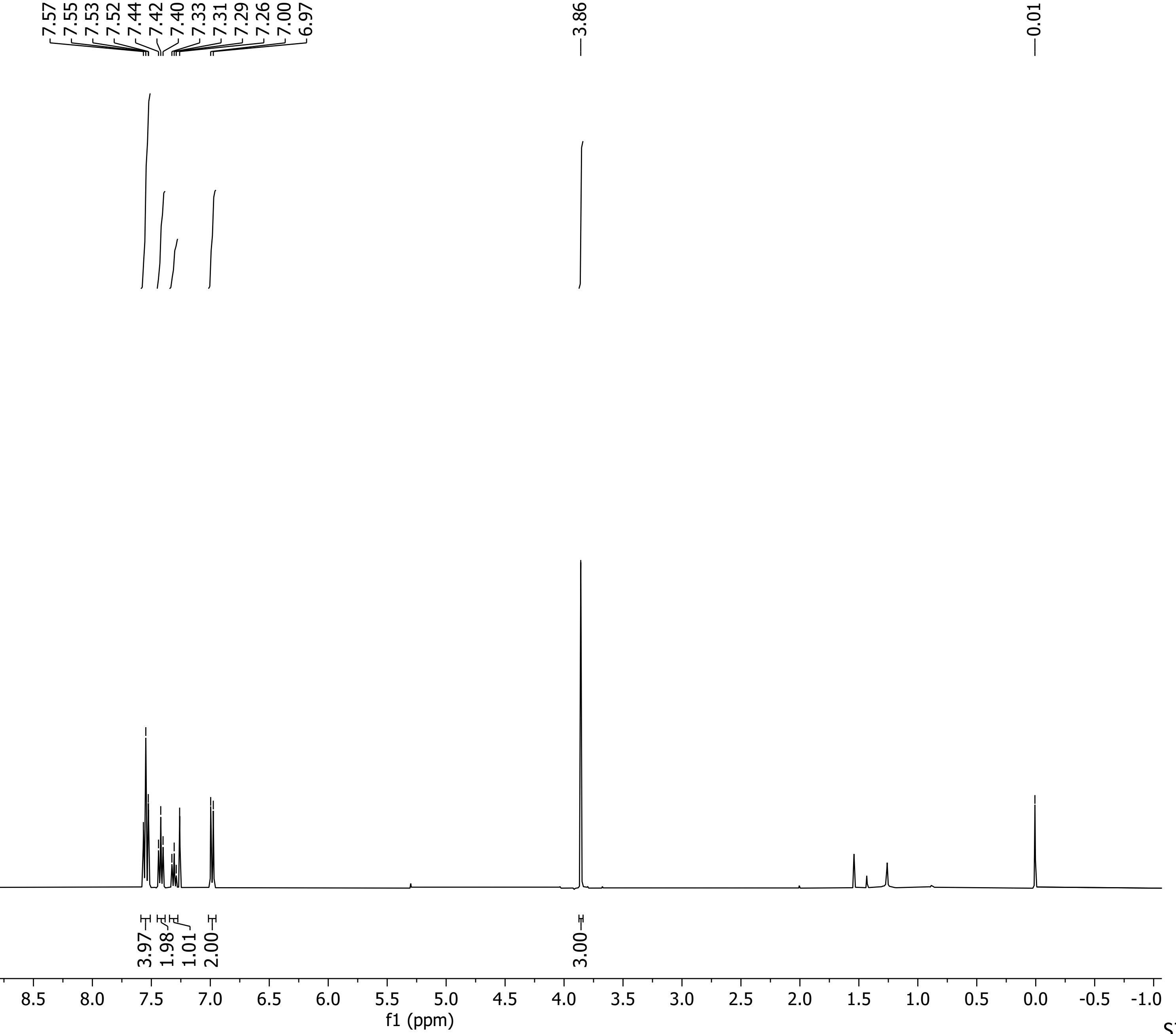

$^{13}C\{^{1}H\}$ **NMR (101 MHz, $CDCl_3$)**

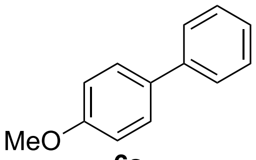

6a

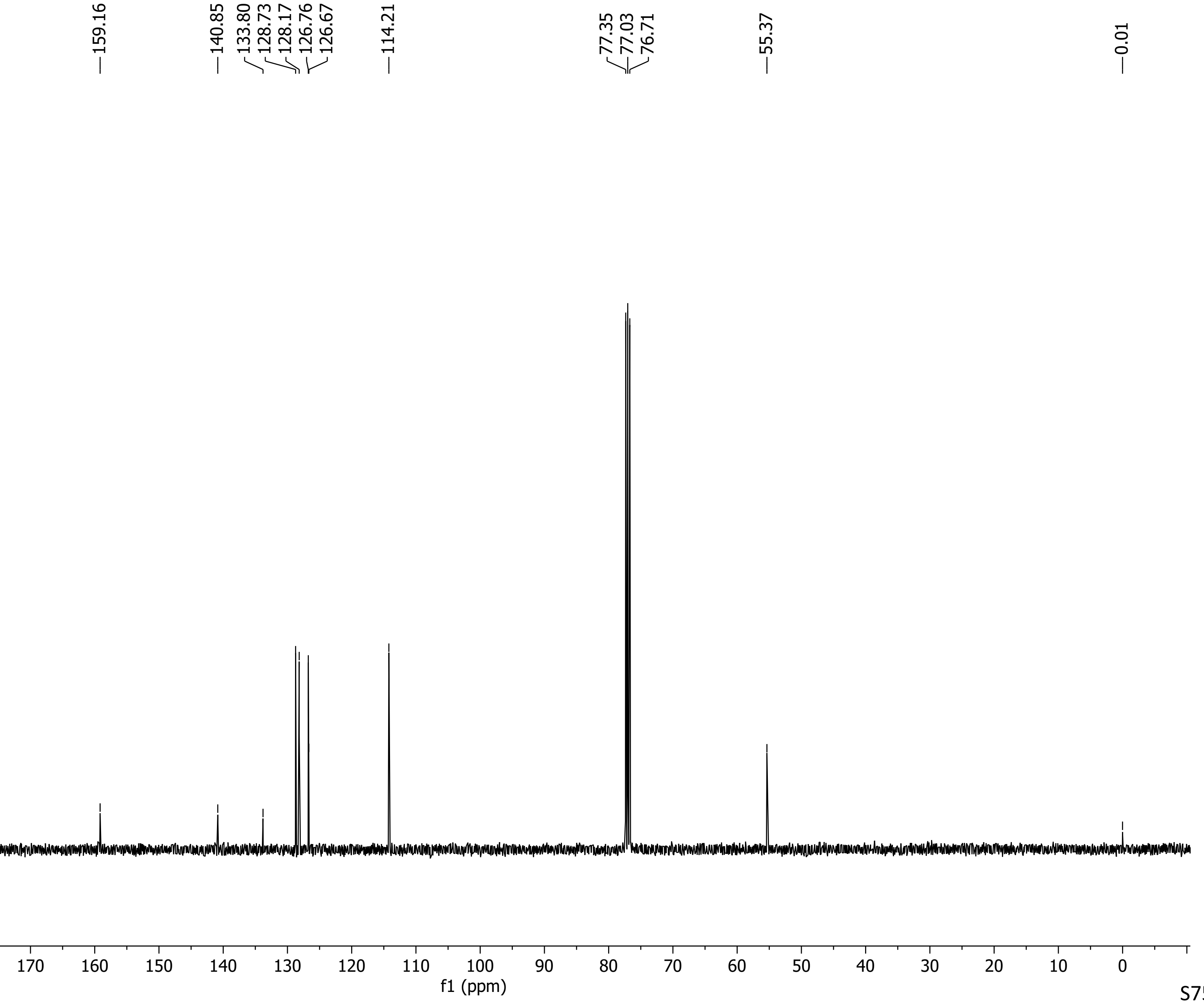

^{1}H NMR (400 MHz, $CDCl_3$)

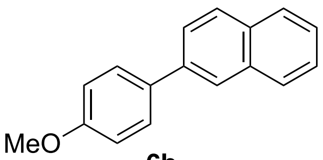

6b

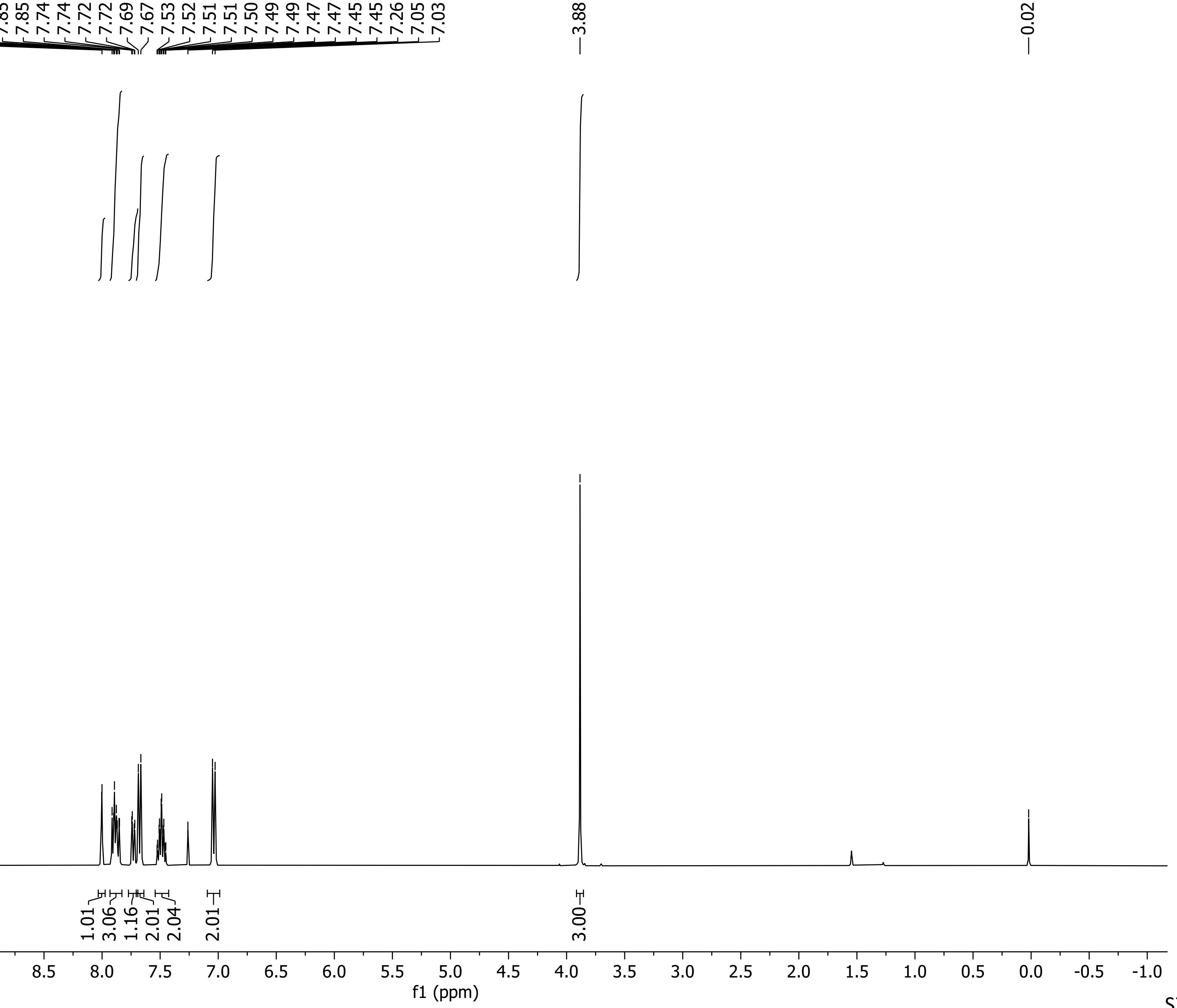

$^{13}C\{^{1}H\}$ NMR (101 MHz, $CDCl_3$)

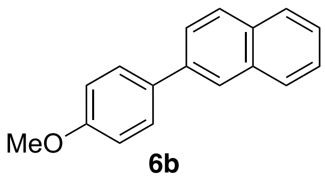

6b

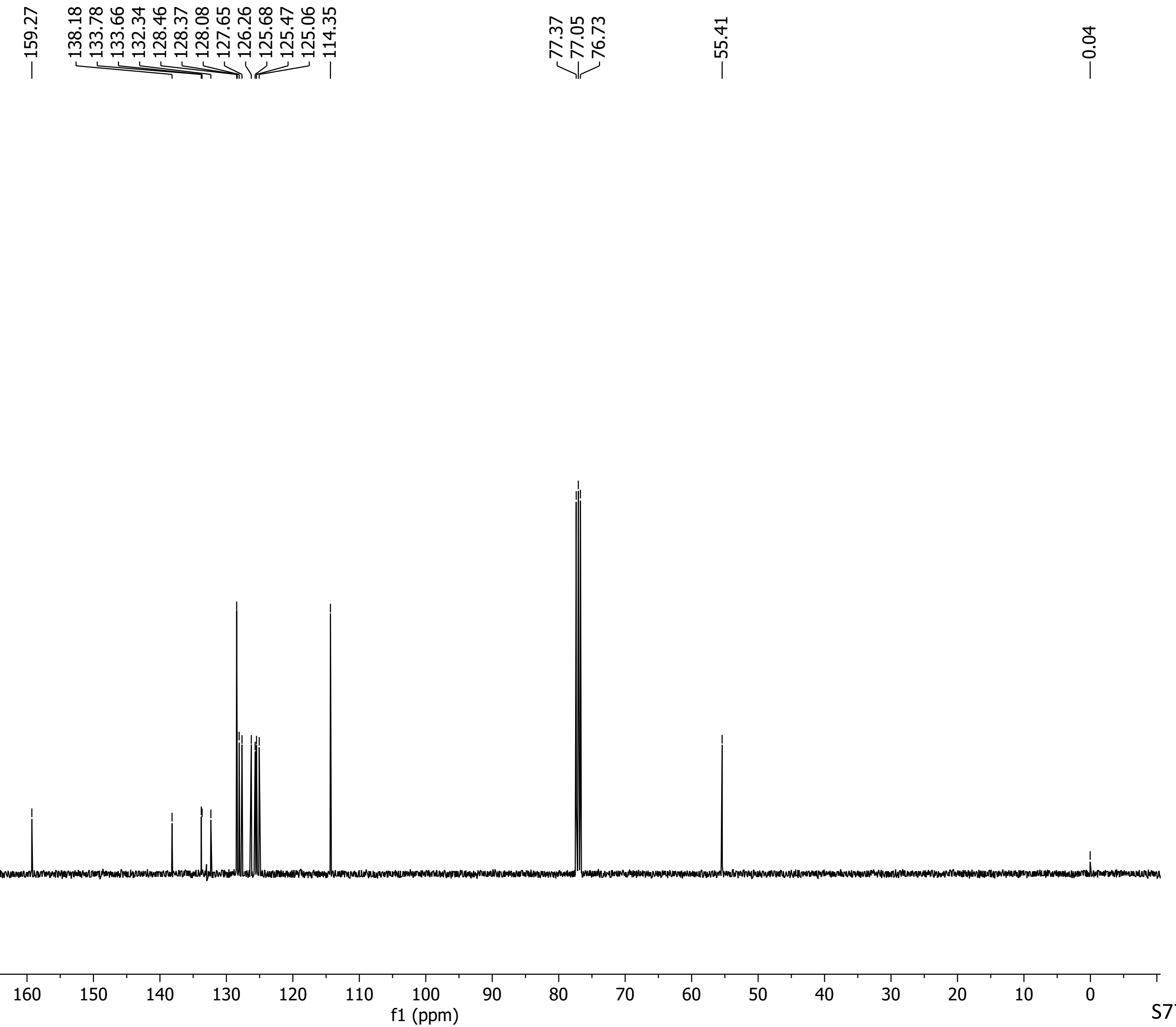

^{1}H NMR (400 MHz, $CDCl_3$)

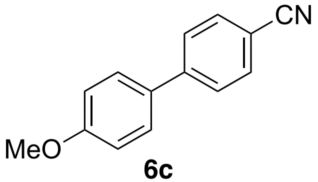

6c

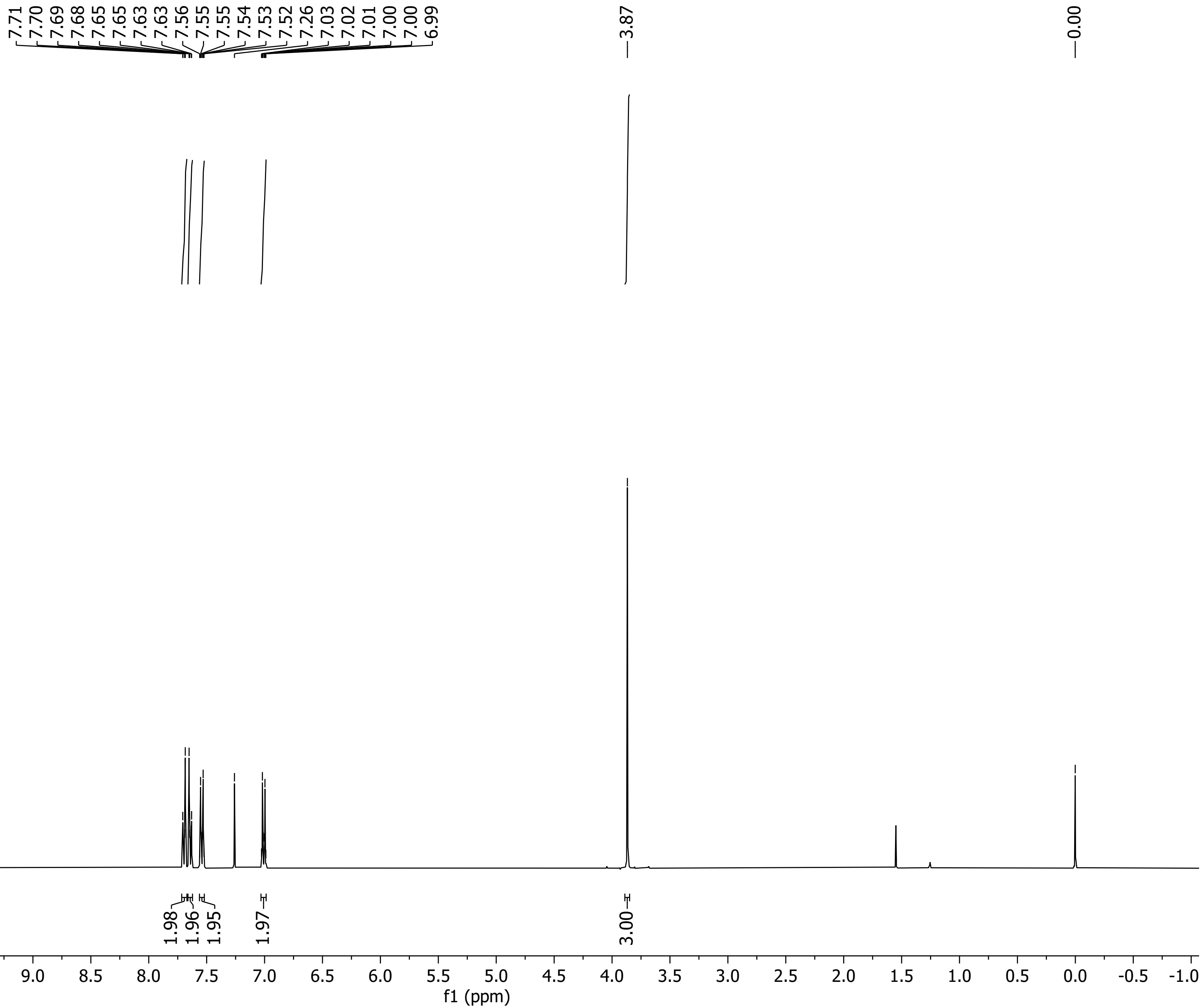

$^{13}C\{^{1}H\}$ NMR (101 MHz, $CDCl_3$)

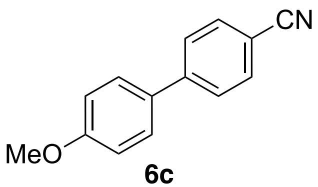

6c

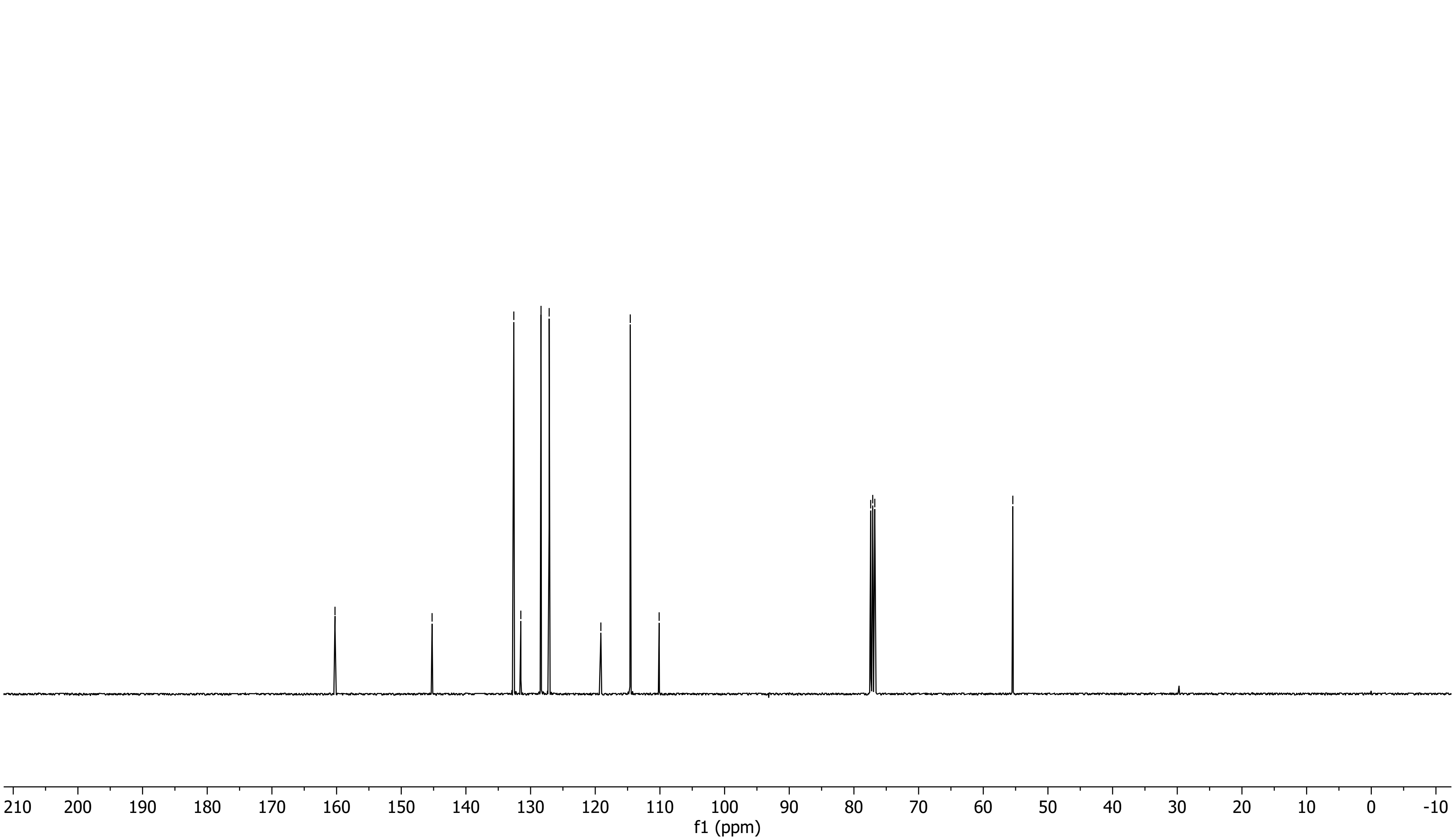

^{1}H NMR (400 MHz, $CDCl_3$)

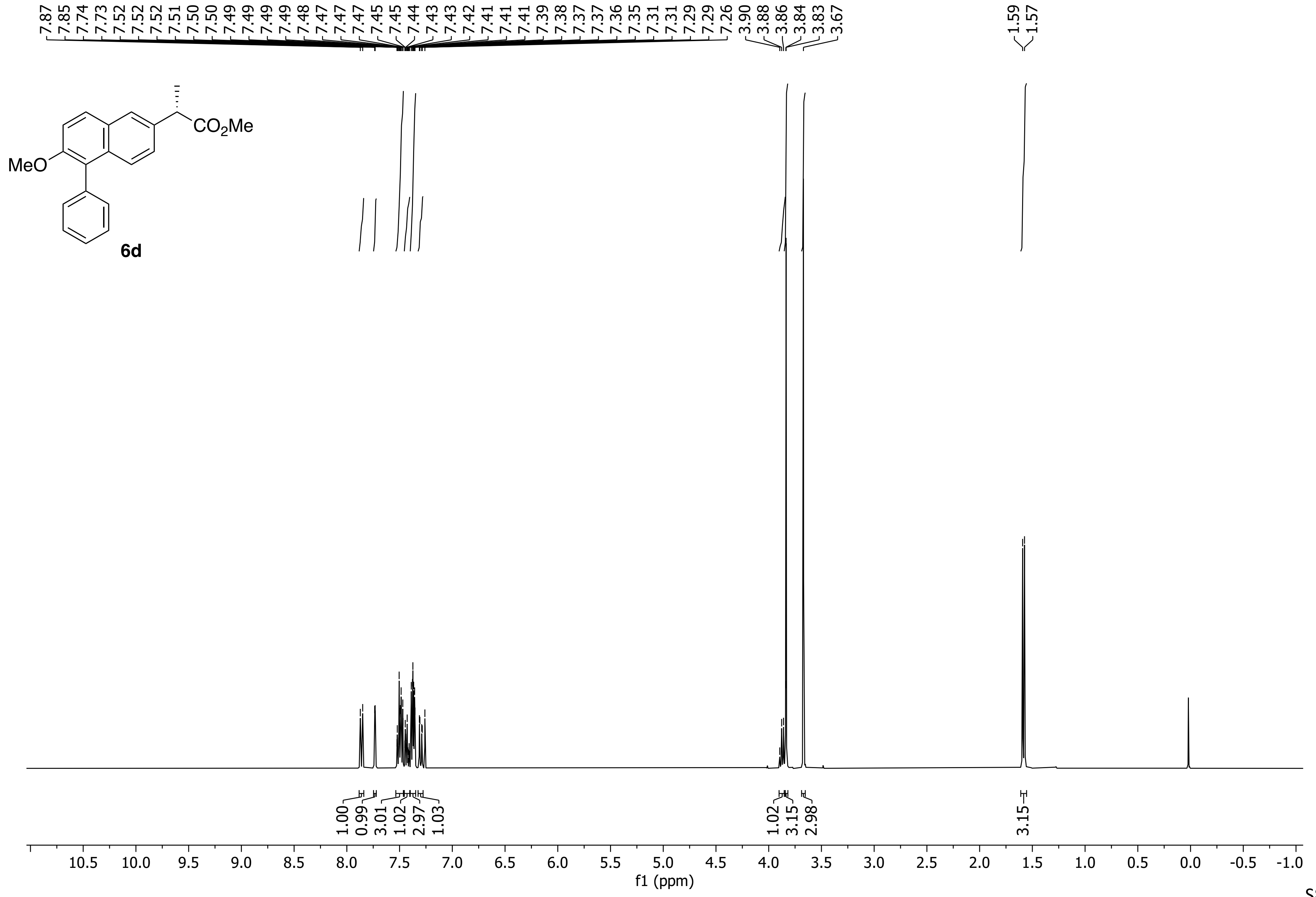

$^{13}C\{^1H\}$ NMR (101 MHz, $CDCl_3$)

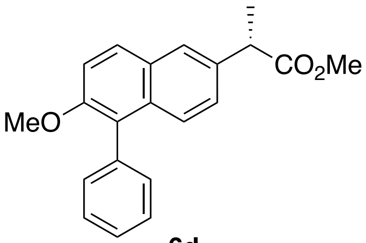

6d

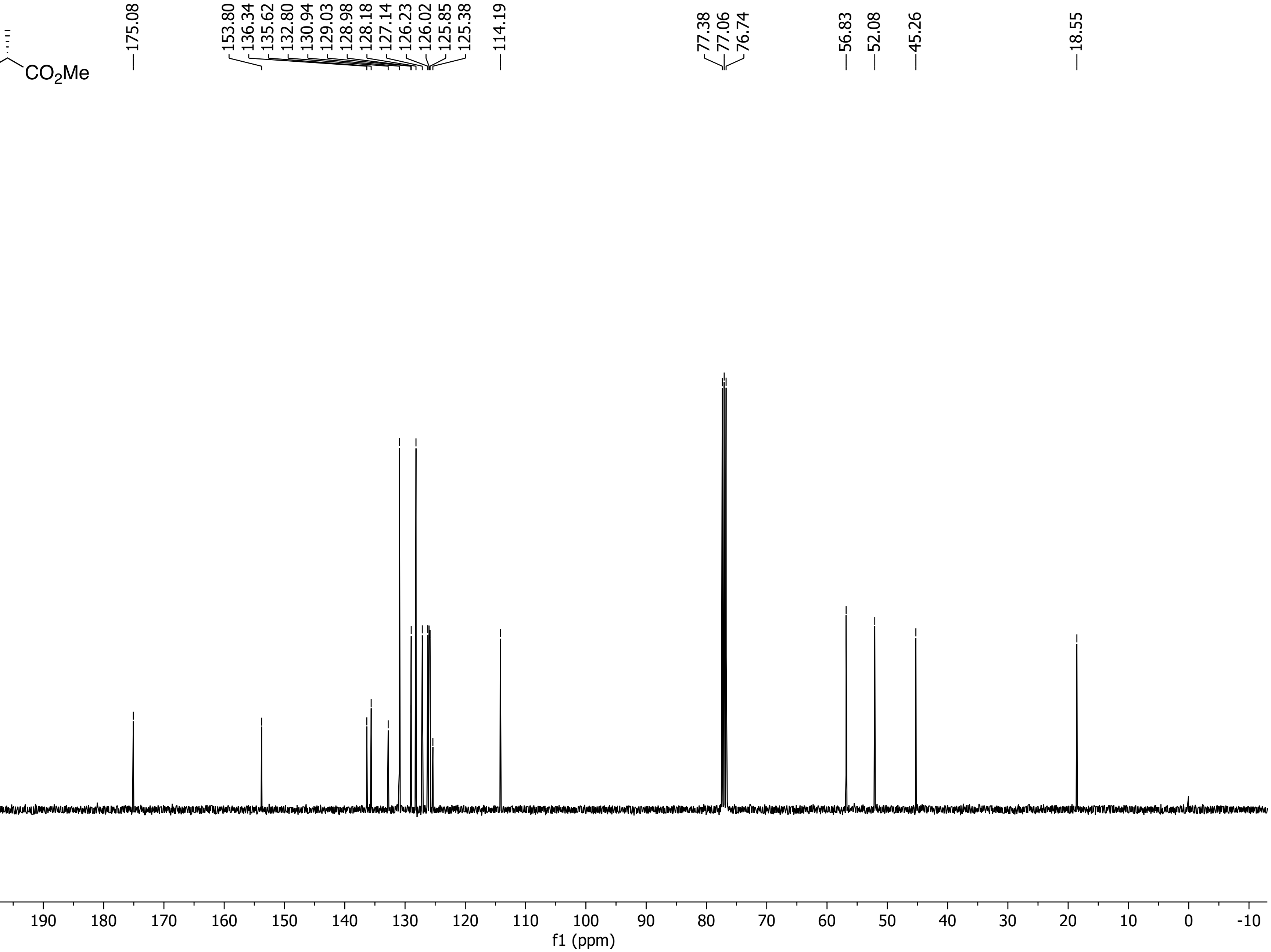

Supplement: Supplementary file 1 [file jo5c03183_si_001.pdf]
